# Group integrated exercise versus recovery class for veterans with posttraumatic stress disorder: a randomized clinical trial

**DOI:** 10.1186/s12888-025-06638-1

**Published:** 2025-02-28

**Authors:** Thomas C. Neylan, Laura A. Muratore, Chanda L. Williams, Martha Schmitz, Courtney V. Valdez, Shira Maguen, Aoife O’Donovan, D. Parker Kelley, Thomas J. Metzler, Beth E. Cohen, Anna C. West, Jordan D. V. Phan, Victor Antonetti, Olga Mayzel, Jennifer A. Hlavin, Margaret A. Chesney, Wolf E. Mehling

**Affiliations:** 1 San Francisco Veterans Affairs Medical Center, San Francisco, CA USA; 2 Department of Psychiatry and Behavioral Sciences, University of California, San Francisco, USA; 3 Northern California Institute for Research and Education, San Francisco, CA USA; 4 Department of Neurology, University of California, San Francisco, USA; 5 Osher Center for Integrative Health, University of California, San Francisco, USA; 6 Department of Medicine, University of California, San Francisco, USA; 7 Department of Family and Community Medicine, University of California, San Francisco, USA; 8 San Francisco VA Health Care System (116-P), University of California, San Francisco, 4150 Clement St., San Francisco, CA 94121 USA

**Keywords:** PTSD, Veteran, Randomized controlled trial, Exercise, Psychoeducation

## Abstract

There are no reported randomized trials testing exercise versus an active comparator for Posttraumatic Stress Disorder (PTSD). This randomized clinical trial assessed the effectiveness of group exercise versus psychoeducation to improve quality of life and reduces symptomatic severity in Veterans with PTSD. Veterans who met criteria for current PTSD (DSM-5) and/or endorsed moderate levels of PTSD symptoms (CAPS 5 score ≥ 23) were randomly assigned to treatment. Integrative Exercise (IE) combines fitness exercises (aerobics, resistance training, stretching) with mindful body/breath awareness versus Recovery Class (REC) psychoeducation control condition. A total of 84 participants were enrolled of which 41 participants were randomized to IE and 43 participants to REC. There were no significant pre-post differences in change in the WHOQOL Psychological Domain in either group. There was a modest reduction in the total CAPS-5 score in both groups (IE: -8.2 (9.9), *p* < .001: REC: -7.8 (2.0), *p* < .001) but no differences across the two conditions. In the IE subsample that was remote, there was a greater improvement in PTSD symptom severity (F[1, 50] = 4.62, *p* = .036) and in in the WHOQOL Psychological Domain (F(1, 47) = 6.46, *p* = .014) in those who attended more sessions.

**Trial registration **ClinicalTrials.gov Identifier: NCT02856412 (registration date: February 27, 2017).

**Supplementary Information:**

The online version contains supplementary material available at 10.1186/s12888-025-06638-1.

## Introduction

There is evidence that aerobic exercise effectively improves many outcomes relevant to Posttraumatic Stress Disorder (PTSD) including fear memory, anxiety, depression, insomnia, cognition, and cardiovascular disease [1–7]. Given that aerobic exercise improves brain health and neurogenesis [8], cognitive function [9], mood [10], sleep [11], and cardiovascular health [12], there is a strong rationale to determine whether exercise may be an effective intervention for Veterans with combat-related PTSD. Individuals with PTSD have lower rates of exercise compared to others without PTSD of the same age and sex [13, 14], suggesting they may particularly benefit from a focus on exercise. Despite the high acceptance of exercise therapy for PTSD [15] and the advantage of a treatment lacking the stigma of conventional mental health practice [16] in this population [17], to date there are no reported randomized trials testing exercise versus an active comparator arm in any population with PTSD.

There is limited published data on the effects of exercise in Veterans with PTSD. There are several pilot studies suggesting that exercise reduces PTSD and associated mood and anxiety symptoms in children [18], adolescents [19], and civilian adults [20–22]. These studies are limited by lack of control conditions [23] and small sample sizes [24]. Nevertheless, these pilot studies indicate acceptability, feasibility, and promise of efficacy [25].

Integrative Exercise (IE) combines traditional fitness exercises (aerobics, resistance training, stretching) with mindfulness, yoga, and mindful body/breath awareness [26, 27]. Group aerobic exercises are a part of daily life of military personnel during their service time. Group exercise is familiar and appealing to Veterans as a self-image boosting and mood enhancing physical practice [23]. In the past decade, while physical activity and aerobic exercise are still emphasized, mindfulness-based practices have increasingly been adopted in military settings [28], and controlled trials of Mind–Body skills [29] and Mindfulness-Based Stress Reduction (MBSR) have demonstrated moderate efficacy for PTSD [30, 31].

Prior pilot testing with a waitlist control demonstrated evidence supporting IE as an effective treatment option for Veterans with symptoms of PTSD [26, 27]. We conducted a randomized controlled trial to evaluate how integrative exercise improves quality of life and reduces PTSD symptoms among Veterans, comparing it to a matched-contact active psychoeducation program typically offered in PTSD clinics. The control condition was a present-focused, Cognitive Behavior Therapy-based coping skills treatment for PTSD, emphasizing recovery strategies and stress management through psychoeducation and skills training [32]. The curriculum includes modules from the Illness Management and Recovery Group manual adapted for PTSD [33, 34], as well as CBT-focused skills targeting the four symptom clusters of PTSD [35]. As an exploratory aim, we investigated differences between the two treatment groups in relation to other health outcomes, including mood, subjective sleep quality, and emotion regulation.

## Methods

### Trial design and procedures

Participants who met eligibility for enrollment were randomly assigned to either 12 weeks of integrative exercise (IE) or PTSD Recovery Class (REC) classes. This research was approved by the Institutional Review Board of the University of California, San Francisco, and the Human Research Protection Program at the San Francisco Veterans Affairs Medical Center. All participants provided written or electronic informed consent. This trial was registered on ClinicalTrials.gov (NCT02856412).

### Participants

**Fig. 1:**
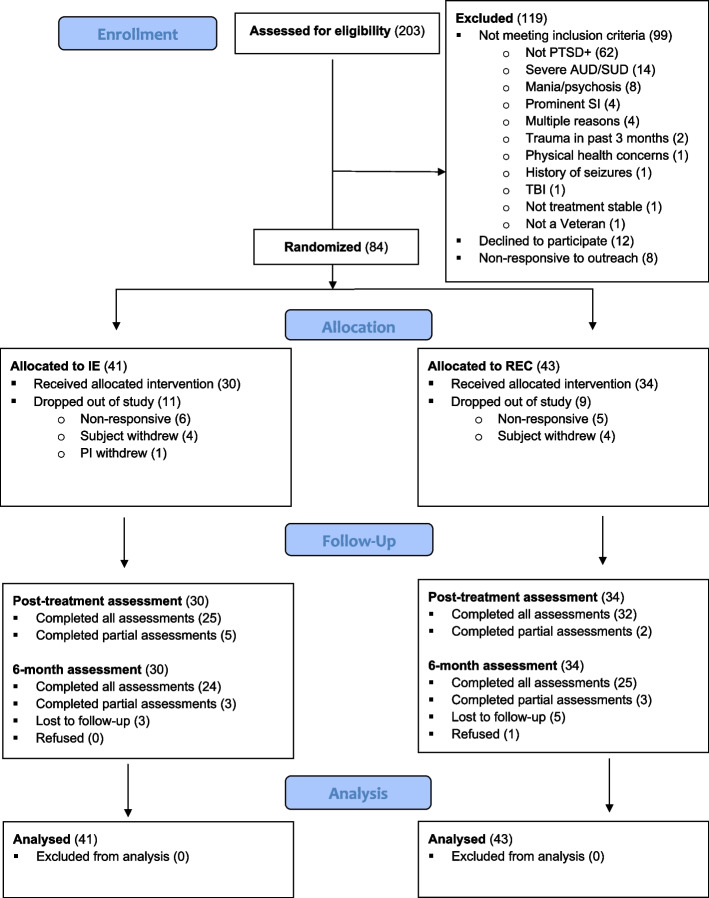
CONSORT flow diagram

A total of 84 Veterans between the ages of 18-75 who met criteria for current PTSD of at least 3-months duration, as defined by the Diagnostic and Statistical Manual of Mental Disorders (DSM-5; American Psychiatric Association [36]) and/or endorsed moderate levels of PTSD symptoms (CAPS 5 score ≥ 23 [37]) were randomly assigned to treatment. Veterans currently receiving mental health treatment (e.g., individual therapy, psychiatric medication) were included if they were treatment stable for at least two months before beginning the trial and maintained this treatment throughout the trial. Exclusion criteria included: exposed to a traumatic event within the past 3 months, psychosis or mania in the past 5 years, severe drug or alcohol use disorder within the past 6 months, clinically significant neurologic disorder, systemic illness affecting CNS function, and/or physical disabilities making it impossible to use exercise equipment. All study participants completed a medical screen to ensure they were physically capable of participating. Medical clearance was determined by examining the participant’s medical history, a physical examination by the study doctor or study nurse, and completion of the Physical Activity Readiness Questionnaire [38]. See Fig. 1 for a detailed diagram of study enrollment and reasons for exclusion.

### Procedures

#### Recruitment, screening, and baseline

Study recruitment and enrollment began in November 2017 and ended in March 2022. Within this enrollment period, study procedures were modified, as described in detail below, due to the COVID-19 pandemic and subsequent restrictions to in-person research. In March 2020, the study transitioned from in-person to a fully remote trial for the duration of enrollment to maintain subject safety.

An extensive outreach plan was implemented to recruit a diverse sample of U.S. Veterans. For Veterans participating locally, in-person, we recruited primarily by mailing letters to Veterans within and surrounding the San Francisco Bay Area, with a smaller number recruited by provider referrals. We expanded recruitment to a national scale when the trial shifted to remote-only procedures in response to the COVID pandemic. For Veterans participating nationwide, remotely, we recruited primarily via TrialFacts, with smaller numbers coming from provider referrals or ResearchMatch. Interested Veterans completed an initial pre-screen interview with study staff to determine whether they met study inclusion (n = 540; see CONSORT diagram, Fig. 1). Potentially eligible Veterans (*n* = 230) provided informed consent and participated in an in-depth clinician-administered diagnostic assessment. Following these assessments, 84 participants were eligible and were randomized. Self-report questionnaires measured subjective sleep quality, PTSD symptoms, mood states, and quality of life.

### Transition from in-person classes to remote delivery

Before COVID, IE group classes were delivered in-person at the San Francisco YMCA. With onset of the epidemic this setting became unsafe for our participants and was changed to online delivery to participants’ homes and their computer or smart phone screens. This limited classes to participants with a minimal amount of electronic equipment and literacy (detailed instructions were delivered as needed by study staff) but at the same time allowed for participation from anywhere in the US with internet connectivity. At the YMCA space, exercise instructors made use of larger equipment such as stepping platforms and wall mounted TRX exercise equipment. Online classes fully relied on yoga mats, elastic resistance bands, dumbbells, and foam rollers that were provided by study staff and sent to participants by mail delivery. Using the zoom platform, participants could see both instructors and all group members in separate screen displays, using either speaker or gallery view. To enable some minimal social group element, which was naturally intrinsic to the in-person interactions before and after classes at the YMCA, participants in online delivered classes spent the last 5 min of each class following the cool-down in group chat on screen.

#### Assessment

Trained master’s level clinical interviewers who were not conducting the treatment and were kept blind to treatment assignment conducted assessments at eligibility baseline, at the post-treatment 12-week assessment, and the post-treatment 6-month follow-up timepoint.

#### Randomization

Eligible participants were randomized (1:1) to either IE or REC treatment classes. A stratified block randomization strategy (i.e., engaged versus non-engaged in current mental health treatment) was employed to ensure conditions did not differ based on current treatment status. Treatment assignments were randomized in a 1:1 ratio in blocks of 6, with separate lists for current treatment strata. Randomization lists were created by the biostatistician (TJM) prior to study start and sent to the study coordinator following completion of eligibility assessment.

#### Treatment and follow-up

Participants assigned to either IE or REC were asked to attend assigned treatment classes 3 times per week for 12 weeks. Each class in IE and REC was 1 h in duration, for a total of 36 treatment hours. Both treatment conditions were group interventions with rolling admission. Treatment adherence was measured by class attendance. Participants completed mid-treatment self-report measures at Weeks 4 and 8 and completed post-treatment assessments immediately following the end of treatment. The post-treatment assessments repeated those at eligibility screening, as described above. Participants were invited to meet with the PI after the 12-week intervention to provide feedback about their experiences. Participants were given the option to electively participate in the alternate arm following the initial 12-week trial.

### Interventions

#### Integrated Exercise (IE)

The IE exercise program was 12 weeks in duration, integrating a combination of aerobic and strength training, stretching and myofascial release exercises with concentration training based on mindful breathing techniques (from yoga and mindfulness approaches recommended by Hoge [39]) aiming at an experiential sense of mind-body integration. Participants exercised in class 3 times weekly, with each total workout being approximately 60 min in length. These sessions initially took place at the Embarcadero YMCA in San Francisco and later in their homes by remote instruction using the Zoom platform. The platform allows for all participants in a class to see each other as well as the instructor(s), attempting to create a group feeling comparable to the in-person classes. All exercise was supervised and documented by two trained professionals to validate adherence and allow for program replications by others as part of an intervention program for PTSD. Exercises were adapted to individual participants’ fitness levels and pre-existing health conditions or injuries.

The IE program was designed to be accessible to veteran from all socio-economic and educational backgrounds, including those who have been in combat, and safe for those with injuries. Specifically, the exercises do not require a gym setting, but can be safely taught indoors as well as outdoors in a group setting and with individuals with a wide range of fitness levels. The exercises do not require machines, but make use of low-cost equipment including yoga mats, hand-held weights (e.g., 2 to 10 pounds) stretch bands of varying elasticity, foam rollers and (only initially in the YMCA setting) stepping platforms and TRX equipment. The IE program is sufficiently different from usual fitness center programs in that it can be conducted in a virtual group setting delivered over an internet platform into a private home [40–43].

Each class followed the same sequence of procedures. Following a moment of welcoming of participants with a brief review of the previous session, every session started and ended with a centering exercise and 3–5 min of mindful breathing. This was followed by the exercise instructor presenting the “password of the week”. In a few sentences they introduced one of the eight attitudes of mindfulness that were reformulated themes based on the weekly topics of the 8-week MBSR program [44]. These included short explanations of the principles of breath awareness, acceptance of the current situation, beginners mind, being non-judgmental about one’s own capacities, etc. Instructors reminded the participants of these attitudes repeatedly as they were to be applied to the exercises in real-time.

The sequence of exercises included a variation of movements and loads for strength training and aerobic activities performed in a variety of ways, including continuous and interval training, as recommended by the American College of Sports Medicine (ACSM) [40–43] and using nonlinear periodization which involves frequent variations in training stimulus and intensity [42]. The aerobic and strength program began with myofascial release exercises, aerobic warm-up exercises, and instructions for postural alignment supporting core engagement. Exercises when feasible engaged all major muscle groups in a systematic fashion that chains body parts together, but varied between sessions, starting with small weights or low-resistance bands, and adding weights, resistance, and aerobic intensity to pre-exhaust levels. Yoga moves and stretches, reminders about breathing and mindfulness are woven into the program. Exercise sessions ended with a 5-min cool-down period that returned to guided mindful breathing, fostering a physically experienced sense of body-based centeredness and relaxed concentration. As described above, props for these exercises include yoga mats, dumbbells, stretch bands, foam rollers, and (initially) stepping platforms.

During the exercises, participants were introduced to deep abdominal breathing and repeatedly reminded to pay attention to their breathing while working out and to dose the intensity of their exercising according to their ability to maintain breathing through his or her nose. Throughout, exercise movements were closely coordinated with breathing phases, e.g., stretches with inhalation and weight pushes or other efforts with exhalation. The mental focus on breathing and exercise-related bodily sensations during all exercises was a key ingredient of this integrative program to provide an embodied sense of mental centeredness and concentration. Mindful breathing is the cornerstone of mindfulness-based intervention for anxiety and depression [45–47].

Generally, classes were led by two instructors, one teaching, the other primarily observing the participants and correcting participants’ exercise form as needed. This became particularly important during the Zoom classes with the relatively small individual zoom frames of the group of participants. Instructors were hired based on long-time experience as both professional fitness and yoga instructors.

#### PTSD recovery class

The REC control condition was a present-focused, CBT, coping skills treatment based on the concept of recovery and predicated on a stress-vulnerability model of mental illness. The curriculum incorporated PTSD psychoeducation, risk management, emotion regulation and distress tolerance skills. It emphasized the practice of coping skills for improving PTSD symptoms [32] and reaching recovery goals. The Recovery group comprises psychoeducation and skills training classes which have been used in the initial or stabilization phase of phased-based treatment prior to trauma-focused evidence-based psychotherapies [32, 35]. Phase-Based care is common in VA settings and there is some limited evidence of modest efficacy of the initial stabilization phase in reducing PTSD symptoms [32, 35]. The curriculum included 6 modules from the Illness Management and Recovery Group manual adapted for PTSD: recovery strategies, reducing stress, coping with persistent symptoms, reducing alcohol and drug use, building social support, and strategies for getting closer to people. The remaining sessions were CBT-focused skills for improving the 4 symptom clusters of PTSD: managing distress related to trauma memories, decreasing avoidance and numbness, reducing hyperarousal and anger, facilitating sleep, reframing negative cognitions, improving trust, communication, and intimacy, addressing moral injury, and increasing self-compassion. Other topics included practical facts about PTSD and complex trauma, stress-vulnerability, interpersonal effectiveness, getting needs met in the VA healthcare system, and living a healthy, values-based lifestyle [34, 48].

### Measures- primary outcomes

#### World Health Organization Quality of Life (WHOQOL-BREF-26 [49, 50])

The 26-item WHOQOL-BREF-26 was used as a self-report measure of quality of life. It comprises four broad domains: physical health (7-items), psychological health (6-items), social relationships (3-items), and environment (8-items). The Psychological Domain, the primary outcome, is derived from 6 items which index body image, negative & positive feelings, self-esteem, spirituality, and cognition. Each of the 6 items have 5 response options with higher scores indicating higher psychological health. The mean score of items within each domain is used to calculate the domain score. The domain score is then linearly transformed to scale of 0-100 to enable comparisons between other domains composed of unequal numbers of items [50]. (Skevington et al. Quality of Life Research 13: 299-310, 2004.)

#### Clinician administered PTSD scale for DSM-5 (CAPS-5 [37])

The CAPS-5 is a clinician-administered measure of PTSD symptom severity that aligns with the DSM-5. The CAPS-5 was used to assess participants current PTSD symptoms (i.e., past 30 days). PTSD diagnoses and total symptom severity scores were used both as a screening assessment for eligibility with CAPS-5 ≥ 23 meeting inclusion criteria, as well as a primary outcome for treatment efficacy.

### Secondary outcomes

#### Secondary measures

##### PTSD checklist for DSM-5 (PCL-5) [51]

The 20-item PCL-5 was used as a self-report measure of PTSD symptoms. The PCL-5 aligns with the DSM-5 descriptions of PTSD symptoms and has been validated in Veteran samples.

##### Godin Leisure-Time Exercise Questionnaire (GLTEQ) [52, 53]

The GLTEQ is a validated brief inventory assessing sedentary, work, recreational, and aerobic activity in a typical week. This metric was used to measure time spent in vigorous activity and was intended to serve multiple purposes: a) It was used to test if randomization effectively balances levels of baseline vigorous activity across the two groups; b) It was used as a manipulation check to ensure that participants randomized to IE engage in more vigorous activity after randomization than participants randomized to REC; c) It was used as a secondary predictor of treatment response.

##### Pittsburgh Sleep Quality Index (PSQI) [54]

The 9-item PSQI was used as our primary measure of subjective sleep quality. Participants were asked to provide self-reported assessments of sleep quality, sleep latency, sleep duration, sleep efficiency, sleep disturbances, use of sedative-hypnotics, and daytime energy over the past week.

##### PSQI Addendum for PTSD (PSQI-A) [55]

The 2-item PSQI-A was used to measure participants’ experiences with disruptive nocturnal behaviors related to PTSD (e.g., hot flashes, nightmares related to traumatic memories) over the past week. The PSQI-A was used as a secondary measure of sleep quality in exploratory analyses.

##### Insomnia Severity Index (ISI) [56]

The 5-item ISI was used as a self-report measure of perceived insomnia severity and was used as a secondary measure of sleep quality in exploratory analyses. Participants responded on a 5-point scale where higher scores indicated greater insomnia severity within the past week.

##### Five Facet Mindfulness Questionnaire (FFMQ) [57, 58]

The FFMQ is a 39-item questionnaire derived from a factor analysis of other mindfulness questionnaires. It assesses five facets of mindfulness: observing, describing, acting with awareness, non-judging and non-reactivity to inner experience which represent elements of mindfulness as it is currently conceptualized. Items are rated on a Likert scale ranging from 1 (never or very rarely true) to 5 (very often or always true). The FFMQ has been shown to have good internal consistency (alpha coefficient range .72 to .92) in several samples and significant relationships in the predicted directions with domains related to mindfulness [57, 58]. The FFMQ allows a detailed assessment of changes as a function of mindfulness and therefore was be used as a secondary outcome.

##### Physical Activity Self-Efficacy scale (PASE) [59]

The PASE was used as an exploratory measure of perceived confidence to continue exercising in the face of competing day-to-day conditions.

##### Difficulties in Emotion Regulation Scale (DERS) [60]

The 36-item DERS was used as a self-report measure of emotion dysregulation. Items were rated on a 5-point scale, 1 (almost never) to 5 (almost always).

##### Emotion Regulation Questionnaire (ERQ) [61]

The 10-item ERQ self-report measure assesses two emotion regulation strategies: cognitive reappraisal and expressive suppression. Both the 6-item cognitive reappraisal scale and the 4-item expressive suppression scale are rated on a 7-point Likert scale from 1 (strongly disagree) to 7 (strongly agree).

##### Multidimensional Assessment of Interoceptive Awareness (MAIA) [62]

The MAIA is a self-report measure recently developed to capture changes in interoception associated with mind–body interventions. The MAIA is a 32-item instrument comprising eight subscales: Noticing, Not-Distracting, Not-Worrying, Attention Regulation, Emotional Awareness, Self-Regulation, Body Listening, and Trusting. Participants rated the items on a 6-point Likert scale, with higher scores indication higher interoceptive awareness. The MAIA assesses regulatory aspects of interoceptive processing and can differentiate between clinically relevant attention styles toward bodily symptoms: anxiety and hypervigilance-driven versus acceptance and mindfulness-based attention [63, 64].

##### Positive States of Mind (PSOM) [65]

The PSOM assesses the capacity for positive states of mind: focused attention, productivity, responsible caretaking, restful repose, sharing, sensuous nonsexual pleasure, and sensuous sexual pleasure. Participants rated the items on a 4-point Likert scale ranging from 0 (unable to have it) to 3 (have it easy). It was found to be internally consistent and sensitive to degrees of life stress.

##### Symptom Check-List-90-Revised (SCL-90-R) [66]

The SCL-90-R is a standard self- report measure of general psychopathology. Scored for nine primary dimensions and three summary indices, the SCL-90-R manual reports extensive reliability and validity data.

##### Feasibility and acceptability questionnaires

This self-report questionnaire was developed for this study completed by IE and REC condition completers at post-treatment. Sixteen items were grouped into three main categories: overall treatment impressions, content of intervention, and length of intervention. (See Supplemental Figs. 1S and 2S) Participants responded to each statement on a 6-point Likert type scale, with 0-2 scores indicating disagreement and 3-5 indicating agreement. Two versions were used so that questions aligned with group assignment (e.g., “exercise” was modified to “topics” for the REC condition when items specified condition content and vice versa). Modified questions included items 5-8 and 13.

### Statistical analysis

We used intent-to-treat analyses, with all participants randomized included in the analyses, including only the stratification variable (concurrent treatment yes/no) and the baseline value of the outcome variable as covariates. All available time point data from any participants lost to follow up were included in the primary analyses, using mixed models (LMM) to accommodate the missing data where possible. The frequency and timing at which outcomes are obtained varies by measure. Some key measures, including the CAPS, were measured at two time points, pre- and post-treatment, while self-report measures such as the WHOQOL, were measured at two additional mid-treatment time points. Measures with more than two measurement occasions make full use of the LMM strategy, whereas LMM’s for pre-post measures reduce to ANCOVA as a special case, except for the added flexibility of modeling heterogeneous group variances.

In analyses with intermediate time points, several modeling choices were considered, including whether to treat the time variable as continuous or categorical, the form of the within-subjects correlation matrix, and whether to allow for heterogeneity of variance across groups and/or time points. For each outcome, the best fitting model was selected according to likelihood ratio tests (for nested models) or the Akaike Information Criterion (AIC; for non-nested models) before examining any coefficients or test statistics. In each case, the best fitting model was one that treated time points as a categorical variable, included participants as the only random effect, and included no additional parameters to accommodate within-participant correlation or heterogeneous group variances. Models were fit using the “mixed” command in Stata v. 16.1 [67]. Residuals were examined to screen for outliers and to ensure that model assumptions were met. No influential outliers were observed, and residual distributions were approximately normal. Secondary analyses focused on effects of attendance and site of delivery (in-person versus remote) on clinical outcomes.

#### Sample size

Power calculations were conducted a priori to determine the adequate sample size needed to detect clinically meaningful between-group effects on our primary variables at pre- and post-treatment time points. Results indicated a sample size of 80 completers (40 per group) was sufficient to yield power of 80% (α = 0.05) to detect standardized effects of d = 0.5, assuming 0.7 within-participant correlations. Obtained sample sizes per group were 30 (IE) and 34 (REC); observed within-participant correlations were *r* = 0.67 for WHOQOL and *r* = 0.45 for CAPS. Actual power to detect an effect of d = 0.50 was 0.64 for the primary WHOQOL outcome and .45 for CAPS scores.

## Results

A total of 84 participants were enrolled of which 41 participants were randomized to IE and 43 participants to REC (see Fig. 1 for consort diagram**)**. Participants assigned to IE had a mean age of 52.7 (SD = 11.6) and were 73% male. Participants assigned to REC had a mean age of 50.8 (SD = 12) and were 65% male. Clinical and demographic characteristics are summarized in Table 1.

**Table 1:** Demographic and clinical characteristics

	REC	IE	*p*-value
	*N*=43	*N*=41	
Age	50.8 (12.0)	52.7 (11.6)	0.47
Sex			0.42
Male	28 (65%)	30 (73%)	
Female	15 (35%)	11 (27%)	
Race			0.11
American Indian/Alaska Native	2 (5%)	2 (5%)	
Asian	5 (12%)	2 (5%)	
Black or African American	9 (21%)	12 (29%)	
White	26 (60%)	18 (44%)	
Other	1 (2%)	7 (17%)	
Ethnicity			0.45
Hispanic or Latino	5 (12%)	9 (22%)	
Non Hispanic or Latino	32 (74%)	27 (66%)	
Unknown	6 (14%)	5 (12%)	
Education			0.65
High School Graduate / GED	2 (5%)	2 (5%)	
Some College	17 (40%)	12 (31%)	
Associates Degree	4 (9%)	5 (13%)	
Bachelors Degree	8 (19%)	6 (15%)	
Some Graduate School	4 (9%)	1 (3%)	
Masters Degree	7 (16%)	12 (31%)	
Doctoral Degree	1 (2%)	1 (3%)	
VA Service Connected Disability			0.85
No	10 (23%)	10 (25%)	
Yes	33 (77%)	30 (75%)	
ASI Alcohol	0.3 (0.3)	0.4 (0.4)	0.10
Smoking History			0.73
Current Smoker	7 (17%)	7 (18%)	
Former Smoker	13 (31%)	9 (23%)	
Never Smoked	22 (52%)	23 (59%)	
CAPS_Total_baseline	29.8 (7.7)	33.1 (8.5)	0.07
PCL5 Total	40.3 (12.9)	43.5 (15.4)	0.31
WHOQOL Physical Health	51.9 (16.0)	51.2 (13.9)	0.82
WHOQOL Psychological	44.4 (14.9)	41.1 (18.5)	0.38
WHOQOL Social Relationships	44.2 (21.6)	45.0 (26.3)	0.88
WHOQOL Environment	59.3 (15.7)	55.9 (16.4)	0.33
WHOQOL Overall Heatlth (Q. 2)	41.9 (25.4)	41.3 (26.3)	0.91
FFMQ Observing	24.6 (7.6)	24.9 (9.6)	0.88
FFMQ Describing	22.8 (8.2)	23.4 (8.7)	0.72
FFMQ Acting with Awareness	23.9 (7.7)	22.8 (8.6)	0.52
FFMQ Nonjudging of Inner Experience	25.0 (9.2)	22.3 (9.4)	0.18
FFMQ Nonreactivity to Inner Experience	19.2 (5.6)	18.9 (6.9)	0.80
GLTEQ Activity Score	27.5 (23.7)	35.2 (35.8)	0.25
Physical Activity Self-Efficacy	2.6 (0.7)	2.9 (1.0)	0.15
DERS Total Score	87.2 (17.4)	88.3 (24.8)	0.81
Emotion Regulation - Cognitive Reappraisal	4.8 (1.0)	4.8 (1.2)	0.87
Emotion Regulation - Expressive Suppression	4.1 (1.4)	4.3 (1.3)	0.46
MAIA Noticing	3.1 (1.2)	3.3 (1.0)	0.31
MAIA Not-Distracting	1.9 (0.8)	1.8 (0.8)	0.55
MAIA Not-Worrying	2.8 (0.6)	2.7 (0.9)	0.48
MAIA Attention Regulation	2.7 (0.9)	3.1 (1.0)	0.08
MAIA Emotional Awareness	3.2 (1.0)	3.4 (1.0)	0.22
MAIA Self Regulation	2.8 (1.0)	3.0 (1.1)	0.43
MAIA Body Listening	2.3 (1.1)	2.5 (1.3)	0.39
MAIA Trusting	2.9 (1.1)	3.0 (1.0)	0.77
Positive States of Mind	10.6 (3.7)	9.4 (4.5)	0.17
SCL90 Global Severity Index	1.1 (0.4)	1.4 (0.7)	0.02
PSQI_base	11.3 (3.4)	12.0 (4.2)	0.39
PSQI PTSD Addendum Global Score	8.0 (4.0)	8.1 (4.1)	0.91
Insomnia Severity Index	14.7 (5.5)	16.8 (6.4)	0.12
Physical Activity Self-Efficacy	2.6 (0.7)	2.9 (1.0)	0.15

Data are presented as mean (SD) for continuous measures, and n (%) for categorical measures. Acronyms: *REC *Recovery Class, *IE *Integrated Exercise, *ASI *Addiction Severity Index, *WHOQOL *World Health Organization Quality of Life, *CAPS *Clinician Administered PTSD Scale, *PCL-5 *PTSD Checklist for DSM5, *SCL-90 *Symptom Check-List-90-Revised, *FFMQ *Five Facet Mindfulness Questionnaire, *GLTEQ *Godin Leisure-Time Exercise Questionnaire, *PASE *Physical Activity Self-Efficacy scale, *DERS *Difficulties in Emotion Regulation Scale, *ERQ *Emotion Regulation Questionnaire, *MAIA *Multidimensional Assessment of Interoceptive Awareness, *PSOM *Positive States of Mind, *PSQI *Pittsburgh Sleep Quality

The sample included 60% and 44% (REC and IE respectively) of white, 21% and 29% black, and 12% and 22% Latin participants. The two groups demonstrated comparable baseline scores on the WHOQOL, CAPS-5, and PCL-5. Table 1 also shows comparable distribution of baseline values on our secondary clinical outcomes. Participants who participated in the trial in person prior to the COVID pandemic were older and more likely to be male, compared to those who participated remotely during the pandemic. Baseline clinical characteristics were otherwise similar in the in person versus remote cohorts. See Supplemental Table 1S.

Table 2 provides information about treatment characteristics in the 2 groups. IE participants who completed the study attended a mean of 20.3 sessions (SD = 10.2) out of the 36 possible. REC participants who completed the study attended a mean of 27.5 sessions (SD = 8.7). Eleven of 41 IE participants dropped out which is similar in ratio to the 9 out of 43 REC participants (X^2^ (1) = 0.40, *p* = 0.53).

**Table 2:** Treatment characteristics

	REC	IE	Contrast
Completion Status			c^2^ (1) = 0.40, *p* = .53
Completed Treatment	34 (79%)	30 (73%)	
Dropped Out	9 (21%)	11 (27%)	
Treatment Location			Fisher Exact *p* = .69
In-Person	15 (37%)	20 (47%)	
Remote	25 (61%)	22 (51%)	
Hybrid In-Person/Remote	1 (2%)	1 (2%)	
Sessions Attended			
Completed, mean (sd) [range]	27.5 (8.7) [6 – 37]	20.3 (10.2) [3 – 37]	
Dropped out, mean (sd) [range]	4.9 (4.4) [0 – 13]	2.7 (3.2) [0 – 9]	
Feasibility and Acceptance Rating (0-6 Scale)	3.9 (0.8) [1.9 – 5.0]	3.7 (1.0) [1.3 – 4.9]	*t(*58) = 0.76, *p *= .45

Dropouts did not differ significantly on the primary outcome measures at baseline, nor on any demographics except for sex. Dropouts were 95% male (19/20) compared to 61% (39/64) for completers, X^2^ (1) = 8.27, *p* = 0.004. The single female dropout was from the REC group. Twenty of the 41 IE participants (47%) and 15 of the 43 REC participants (37%) completed the intervention in person prior to the COVID pandemic. Two participants, one in each group, completed the intervention in a hybrid of in-person and remote participation. Feasibility and acceptability ratings were mixed, but overall satisfaction with both interventions was high. See Supplemental Figs. 1S and 2S.

### Primary outcome analyses

**Fig. 2:**
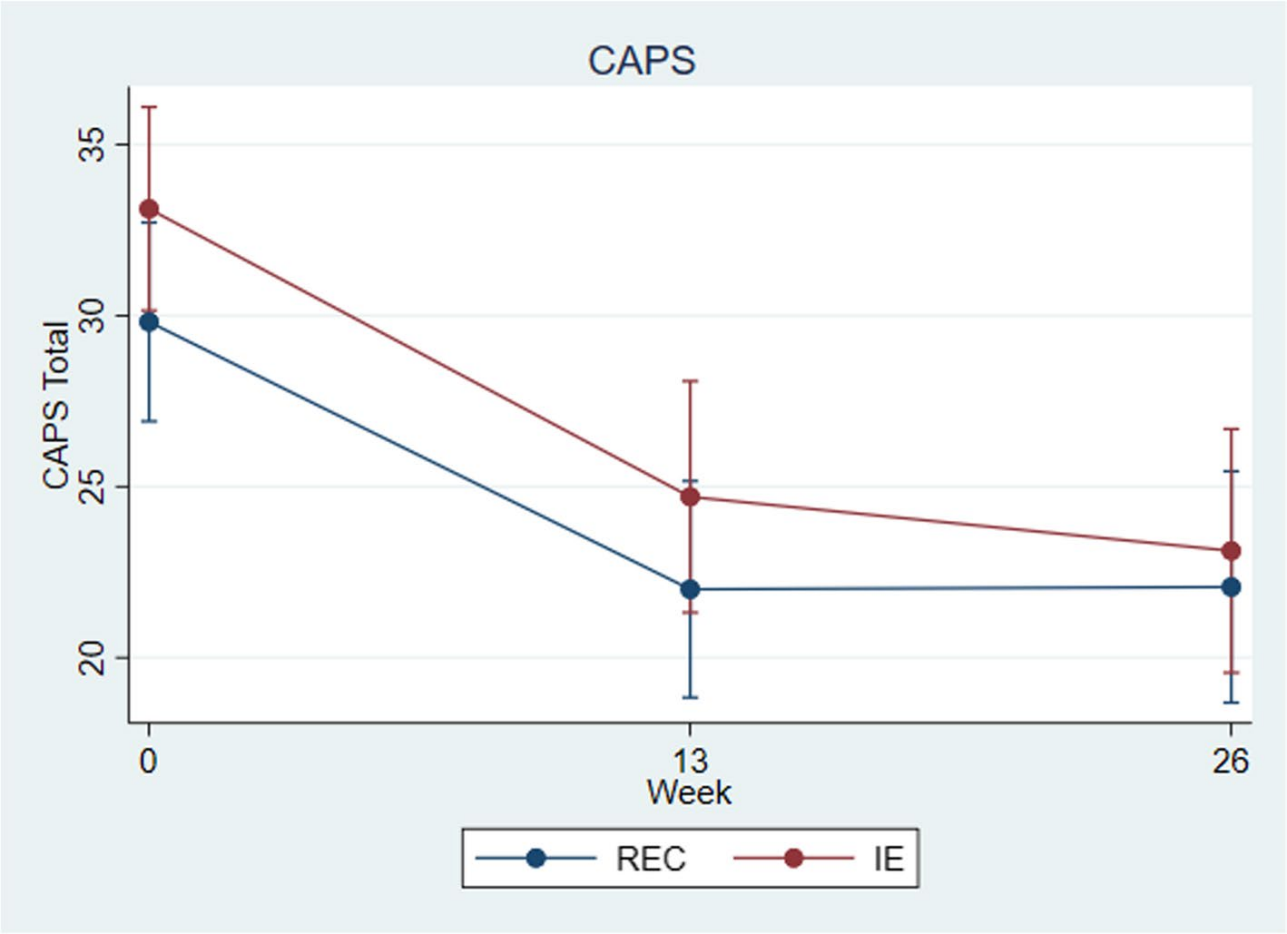
Clinician administered PTSD scale

**Fig. 3:**
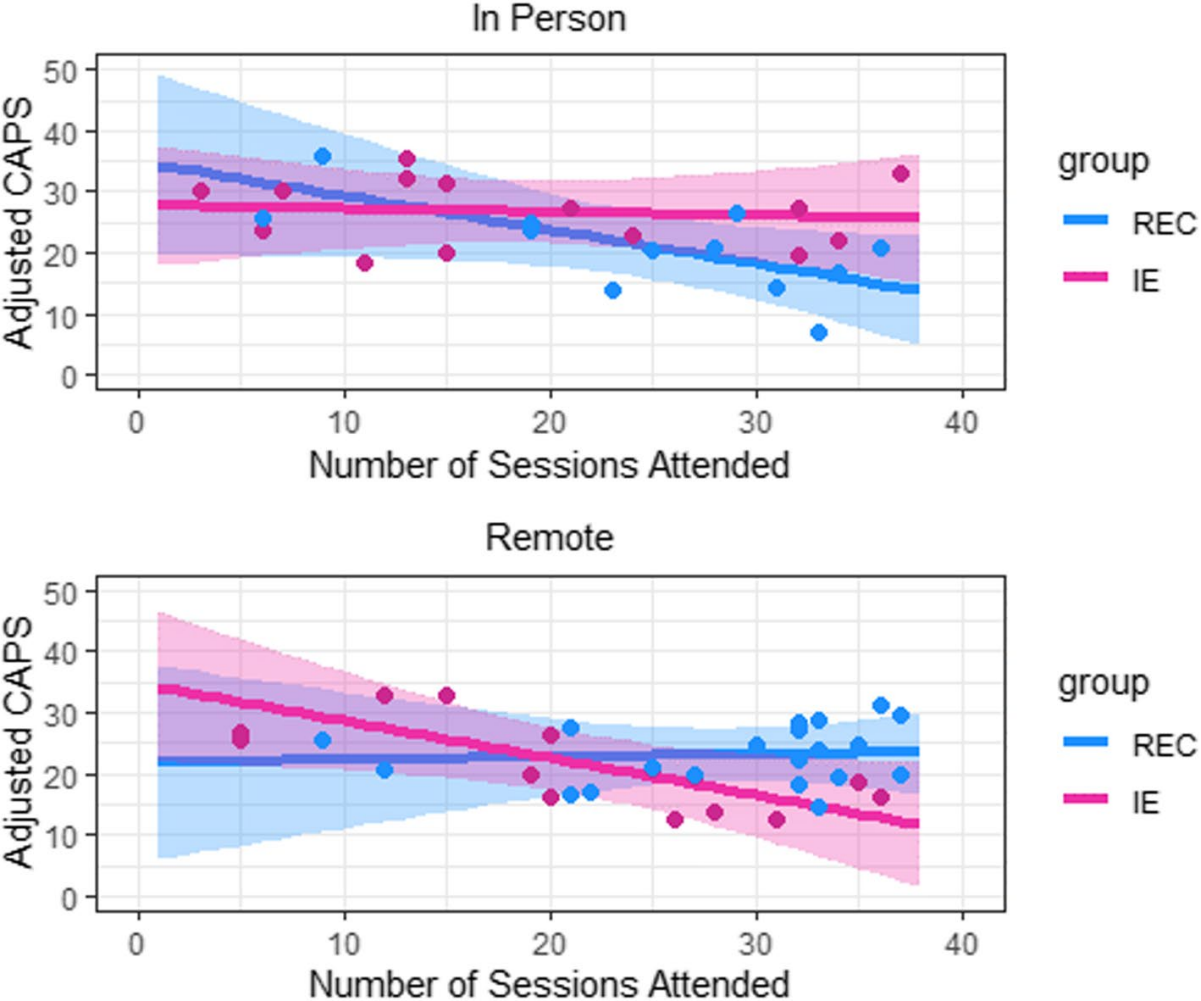
Change in CAPS 5 in IE and REC conditions by setting and number of attended sessions

**Fig. 4:**
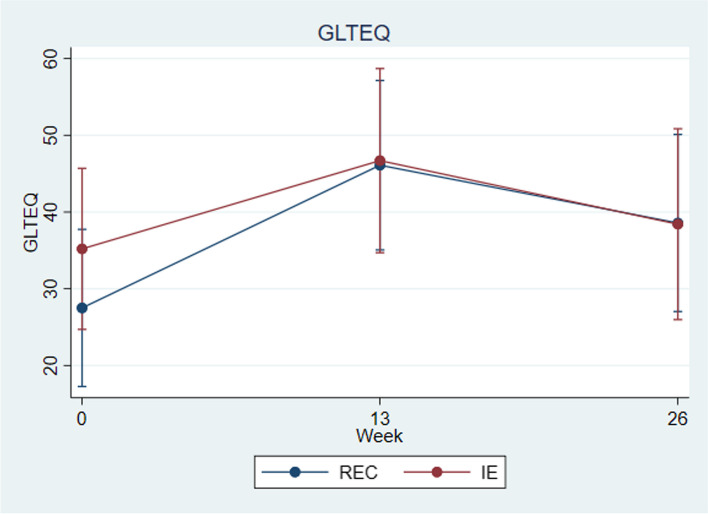
Godin leisure-time exercise questionnaire

There were no differences in IE versus REC in change in the WHOQOL Psychological Domain (see Table 3 and Supplemental Fig. 3S). Further, there were no significant pre-post changes in WHOQOL Psychological Domain in the combined sample. There were no significant differences in changes in the CAPS-5 total score in IE versus REC (see Table 3 and Fig. 2). There was a modest reduction in the total CAPS-5 score in both groups (IE: −8.2 (9.9), p < 0.001: REC: −7.8 (2.0), p < 0.001). In the IE subsample that was remote, there was a significantly greater improvement in PTSD symptom severity (F[1, 50] = 4.62, *p* = 0.036; Fig. 3) and in in the WHOQOL Psychological Domain (F(1, 47) = 6.46, *p* = 0.014; Fig. 4S) in those who attended a greater number of sessions.

**Table 3:** Major outcomes by treatment group

	PTSD Recovery Group (Control)			Integrative Exercise Group (Treatment)			Between-Group Contrast^a^
Outcome	Baseline	Post Tx	Pre-Post Change	Baseline	Post Tx	Pre-Post Change	
WHOQOL-Psychological	44.1 (15.1)	45.2 (16.8)	1.1 (12.0), *p* = .59	39.4 (16.8)	41.5 (19.4)	2.2 (16.2), *p* = .49	t (55) = 0.01, *p* = .989
WHOQOL-Physical	52.2 (16.1)	54.8 (15.8)	2.6 (12.5), *p* = .24	50.5 (14.1)	54.5 (15.9)	4.0 (14.1), p = .16	t (55) = 0.52, *p* = .602
CAPS Total	29.8 (7.9)	22.0 (11.3)	-7.8 (2.0), *p* < .001	32.7 (7.9)	24.4 (11.1)	-8.2 (9.9), *p* < .001	t (58) = 0.39, *p* = .699
PCL5	38.9 (13.3)	39.9 (15.7)	1.0 (13.4), *p* = .67	40.8 (15.6)	36.3 (17.5)	-4.4 (12.5), *p* = .08	t (55) = 1.45, *p* = .152
SCL-90 Global Severity	1.09 (0.43)	1.13 (0.57)	0.04 (0.43), *p* = 59	1.27 (0.66)	1.14 (0.68)	-0.16 (0.40). *p* = .06	t (54) = 1.53, *p* = .133
FFMQ - Obs	25.2 (7.2)	26.4 (7.1)	1.2 (4.4), *p* = .12	25.5 (7.5)	22.2 (11.4)	-3.2 (11.0), *p* = .12	t (59) = 2.16, p = .035
FFMQ - Desc	22.6 (7.6)	24.4 (7.6)	0.8 (4.1), *p* = .26	24.4 (6.7)	21.8 (11.3)	-2.6 (11.6), *p* = .23	t (59) = 1.46, p = .149
FFMQ - Aware	23.6 (6.9)	24.0 ( 7.1)	0.4 (3.3), *p* = .47	23.8 (6.9)	20.1 (10.1)	-3.7 (9.8), *p* = .04	t (59) = 2.26, *p* = .028
FFMQ - Nonjudge	25.4 (8.6)	25.5 (7.8)	0.2 (5.4), *p* = .85	25.0 ( 8.2)	21.2 (11.5)	-3.8 (7.9), *p* = .01	t (59) = 2.31, *p* = .024
FFMQ - Nonreact	19.2 (4.7)	19.9 (5.3)	0.7 (2.7), *p* = .12	19.9 (5.7)	17.7 (8.9)	-2.2 (8.6), *p* = .16	t (59) = 1.80, *p* = .077
GLTQ	25.7 (20.8)	43.8 (36.0)	18.1 (29.7), *p* < .01	30.6 (29.2)	44.2 (40.2)	13.6 (27.2), *p* = .02	t (53) = 0.59, *p* = .559
PASE	2.4 (0.7)	2.6 (0.7)	0.2 (0.5), *p* = .08	2.7 (0.8)	2.7 (0.9)	0.0 (0.0), *p* = .99	t (53) = 0.21, *p* = .834
DERS Total	89.1 (16.7)	91.3 (20.2)	2.2 (14.3), *p* = .39	85.9 (17.7)	85.7 (19.4)	-0.2 (17.1), *p* = .94	t (53) = 0.84, *p* = .407
ERQ - Reappraisal	4.7 (0.9)	4.7 (1.1)	0.0 (1.0), *p* = .84	4.7 (1.1)	4.7 (1.5)	0.1 (1.0), *p* = .99	t (52) = .070, *p* = .947
ERQ – Suppression	4.0 (1.5)	3.7 (1.3)	-0.3 (0.9), *p* = .12	4.1 (1.1)	4.1 (1.4)	0.0 (0.9), *p* = .87	t (52) = 1.18, *p* = .245
MAIA – Emot. Awareness	3.1 (1.1)	3.5 (0.8)	0.4 (1.0), *p* = .03	3.3 (1.0)	3.3 (0.8)	0.1 (0.8), *p* = .64	t (53) = 1.16, *p* = .25
MAIA – Attention Reg.	2.7 (0.9)	2.9 (0.9)	0.2 (0.8), *p* = .09	2.8 (0.9)	3.0 (0.8)	0.2 (0.8), *p* = .29	t (53) = 0.18, *p* = .860
MAIA - Noticing	3.0 (1.1)	3.2 (1.0)	0.2 (1.0), *p* = .32	3.3 (1.0)	3.3 (1.0)	0.0 (0.9), *p* = .87	t (53) = 0.13, *p* = .896
MAIA – Not Distracted	2.0 (0.8)	1.6 (0.7)	-0.4 (0.6), *p* <.01	1.8 (0.9)	1.7 (0.9)	-0.1 (0.6), *p* = .65	t (53) = 1.55, *p* = .126
MAIA – Not Worry	2.7 (0.6)	2.7 (0.7)	-0.1 (0.5), *p* = .44	2.8 (0.8)	2.9 (0.6)	0.2 (0.6), *p* = .22	t (53) = 0.26, *p* = .055
MAIA – Self Regulation	2.7 (1.0)	3.0 (0.7)	0.3 (0.8), *p* = .06	2.9 (1.1)	3.1 (1.0)	0.2 (1.1), p = .42	t (53) = 0.03, *p* = .975
MAIA – Body Listening	2.3 (1.1)	2.7 (1.0)	0.4 (1.0), *p* = .023	2.4 (1.3)	2.7 (1.1)	0.3 (1.1), p = .12	t(56) = 0.01, *p* = .993
MAIA - Trust	2.9 (1.2)	3.1 (1.1)	0.2 (0.9), *P* = .23	2.9 (1.0)	3.1 (0.9)	0.2 (1.0), *P* = .28	t(55) = 0.22, *p* = .830
PSOM	10.9 (3.6)	11.3 (3.9)	0.4 (2.4), *p* = .33	9.7 (3.9)	8.9 (6.9)	-0.8 (4.9), *p* = .38	t (59) = 1.55, *p* = .127
PSQI	11.1 (3.7)	10.7 (4.2)	0.4 (4.0), *p* = .61	11.9 (4.5)	11.4 (5.2)	0.4 (4.1), *p* = .58	t (55) = 0.18, *p* = .858
ISI	15.0 (5.9)	13.2 (5.4)	-1.8 (5.0), *p* = .04	16.0 (6.2)	13.6 (6.8)	-2.4 (5.3), *p* = .029	t (54) = 0.24, *p* = .810

Data are presented as mean (SD) for all measures. Acronyms: *WHOQOL *World Health Organization Quality of Life, *CAPS *Clinician Administered PTSD Scale, *PCL-5 *PTSD Checklist for DSM5, *SCL-90 *Symptom Check-List-90-Revised, *FFMQ *Five Facet Mindfulness Questionnaire, *GLTEQ *Godin Leisure-Time Exercise Questionnaire, *PASE *Physical Activity Self-Efficacy scale, *DERS *Difficulties in Emotion Regulation Scale, *ERQ *Emotion Regulation Questionnaire, *MAIA *Multidimensional Assessment of Interoceptive Awareness, *PSOM *Positive States of Mind, *PSQI *Pittsburgh Sleep Quality Index, *ISI *Insomnia Severity Index

^a^Group difference in post-treatment scores adjusted for baseline

### Responder analyses

Table 4 shows that there were no differences in response rate (10-point drop in CAPS-5: IE = 41%, REC = 44%), loss of diagnosis, or remission status on the CAPS-5 outcome for the 2 groups.

**Table 4:** Response rate^a^, loss of diagnosis, remission^b^ across treatment groups

	Number (%) Posttreatment IE *n* = 29	REC *n* = 34
Response	12 (41%)	15 (44%)
Loss of Diagnosis	10 (32%)	10 (29%)
Remission	4 (13%)	5 (15%)

^a^Defined as an improvement of at least 10 points on CAPS-5

^b^Defined as loss of diagnosis and CAPS-5 < 12

### Secondary outcomes

Self-reported physical activity as indexed by the Godin Leisure Time Questionnaire showed comparable levels of physical activity at baseline (See Table 1 and Fig. 4). However, post-randomization scores on this measure did not demonstrate that IE participants became more physically active than REC participants. Both groups showed an increase in vigorous activity from pre- to post- treatment (See Fig. 4).

Six-month follow-up data showed some regression toward baseline levels of activity. Self-report PTSD symptoms on the PCL-5 did not change significantly over the trial (See Table 3). Mindfulness as indexed on the FFMQ showed that in the condition (IE) where they were taught mindfulness attitudes, participants self-reported feeling less skilled in this domain over the course of the trial. The REC group reported some modest improvement in interoceptive awareness on several items of the Multidimensional Assessment of Interoceptive Awareness (MAIA) scale. Both groups had small reductions in self-reported insomnia. Other secondary outcomes as described in the Supplement did not show significant changes over the trial (See Table 3).

### Participant feedback

The PI interacted with 46 participants who accepted the invitation to meet after the 12-week intervention either in person or by phone. He encouraged candid feedback and asked for suggestions to improve the IE or REC programs. Participants commented on the class instructors, scheduling issues, class timing such as 3-h later evening hours on the East Coast, logistical difficulties with in-person classes, and limited peer interaction with the Zoom version of IE. The staggered entry into ongoing classes due to rolling enrollment was challenging for some participants, and peer interaction was limited when group sizes went beyond five or six. The great majority commented very positively about enjoying the classes, the instructors, the class content, and the ability to participate remotely from their home. Several participants also described greater physical fitness after completing the classes.

## Discussion

Contrary to our hypotheses, we did not show that participation in either group IE or REC resulted in a significant improvement in psychological quality of life as indexed by the WHOQOL. Participation did result in a modest decline in PTSD symptom severity as indexed by the CAPS-5. Response rates in both groups were below 50% and remission from treatment was uncommon (13-15%). Surprisingly, both groups reported an increase in physical activity over the course of the 12-week intervention. Both treatment arms were associated with mixed responses on some aspects of feasibility and acceptability, though there was overall high satisfaction with both treatment arms.

The conduct of this study was impacted by the COVID pandemic and both arms were converted from an in person to a remote telehealth format. Preliminary data suggest that higher attendance was associated with greater reduction in the CAPS-5 total score in the remote condition for the IE condition. This may have been due to the convenience offered by the telehealth format, in contrast to Veterans having to drive considerable distances to attend classes at the location where the IE condition was taught.

A notable contrast between the two study interventions is that the IE groups were led by exercise instructors who were not clinically trained. In contrast, the REC condition was led by experienced PTSD psychologists who delivered the same intervention focused on education and skills training commonly used as a present-focused treatment in VA PTSD treatment clinics. Although both study arms were highly rated by participants for treatment satisfaction, the magnitude of symptom reduction was small in the full sample, but substantial in those who engaged in greater than 20 sessions in the remote IE condition.

There was an unexpected difference across the two treatment arms on the self-report measure of mindfulness. The IE participants who were specifically taught initial mindfulness techniques or attitudes, rated themselves as less mindful at the end of the 12-week condition. Experience with conducting mindfulness trials has shown that untrained participants may initially overestimate and post-intervention rate themselves lower on specific skills as they become more aware of different aspects of mindfulness [68, 69]. This suggests that the amount of mindfulness training incorporated into the IE intervention is insufficient to produce durable improvements in mindfulness skills.

The modest reduction in the CAPS-5 score in the IE condition was considerably smaller than the magnitude of improvement in the pilot trial using the same intervention [26]. Overall, there was a compression of change scores in both arms compared to both arms of the pilot trial, which had a waitlist control. There are several differences in treatment delivery across the two trials. First, the IE condition was delivered in evening hours in the pilot study, compared to afternoon hours in the current study. Second, the mean age of participants in the IE arm was older in the current trial (mean age = 52.7) versus the IE condition of the pilot study (mean age = 47.4). Third, the pilot was conducted prior to the pandemic. Results from current study show a possible impact of attendance on clinical outcomes and it is notable that participants randomized to IE attended fewer sessions (mean = 20.3) than REC participants (mean = 27.5). It is possible that the IE condition involves more frustration and possibility for self-critical responses for some participants when engaged in vigorous exercise. Finally, compared to the in-person pilot study, opportunities for social interaction during or around the remote classes were more limited in the IE condition.

## Conclusion

The study results do not demonstrate that an exercise condition that integrates mindful breathing, aerobic and strengthening activity, produces a greater change in self-reported psychological quality of life or reduction in PTSD symptoms relative to an active intervention that is comparable to present-focused treatments offered in many PTSD specialty clinics in the VA. Both current standard treatment by experienced psychologists and an integrative exercise program led by fitness and yoga instructors had similar effects. Despite high overall satisfaction of study participants, the magnitude of change in PTSD symptom severity is modest and response rates are below 50%. Higher attendance in the remote IE condition was associated with greater symptom improvement. Future work should attempt to incentivize more consistent engagement in exercise sessions to promote more clinically meaningful symptom reduction.

## Supplementary Information

Supplementary Material 1.

Supplementary Material 2.

Supplementary Material 3: Supplemental Figure 1S. Individual Responses to Feasibility and Acceptability Questionnaire- Integrated Exercise Group. Participants responded to each statement on a 6-point Likert type scale, with 0-2 scores indicating disagreement (tan colors) and 3-5 (green colors) indicating agreement. Supplemental Figure 2S. Individual Responses to Feasibility and Acceptability Questionnaire- Recovery Group. Participants responded to each statement on a 6-point Likert type scale, with 0-2 scores indicating disagreement (tan colors) and 3-5 (green colors) indicating agreement. Supplemental Figure 3S: WHOQOL Psychological Domain. Supplemental Figure 4S. Change in WHOQOL Psych in IE and REC conditions by setting and number of sessions attended. Supplemental Table 1S Demographic and Clinical Characteristics of In-Person and Remote Samples.

## Data Availability

The datasets used and/or analysed during the current study are available from the corresponding author on reasonable request.

## Abbreviations

AIC: Akaike Information Criterion
ASI: Addiction Severity Index
ANCOVA: Analysis of Covariance
ACSM: American College of Sports Medicine
CAPS: Clinician Administered PTSD Scale
CBT: Cognitive Behavioral Therapy
DERS: Difficulties in Emotion Regulation Scale
DSM: Diagnostic and Statistical Manual of Mental Disorders
ERQ: Emotion Regulation Questionnaire
FFMQ: Five Facet Mindfulness Questionnaire
GLTEQ: Godin Leisure-Time Exercise Questionnaire
IE: Integrative Exercise
ISI: Insomnia Severity Index
LMM: Linear Mixed Model
MAIA: Multidimensional Assessment of Interoceptive Awareness
MBSR: Mindfulness-Based Stress Reduction
PASE: Physical Activity Self-Efficacy scale
PCL: PTSD Checklist
PSOM: Positive States of Mind
PSQI: Pittsburgh Sleep Quality Index
PSQI-A: Pittsburgh Sleep Quality Index Addendum for PTSD
PTSD: Posttraumatic Stress Disorder
REC: Recovery Class
SCL-90-R: Symptom Check-List-90-Revised
SD: Standard Deviation
VA: Veterans Affairs
WHOQOL: World Health Organization Quality of Life
YMCA: Young Men's Christian Association
