# Supplementary material for: Group integrated exercise versus recovery class for veterans with posttraumatic stress disorder: a randomized clinical trial
Source: BMC Psychiatry. 2025 Feb 28;25:185. doi: 10.1186/s12888-025-06638-1 (PMC11871828; doi:10.1186/s12888-025-06638-1)
Supplement: Supplementary file 2 — Supplementary Material 2. [file 12888_2025_6638_MOESM2_ESM.pdf]

Study Protocol and Statistical Analysis Plan

Title: Improving Mind/Body Health and Functioning with Integrative Exercise

Date: August 7, 2023

NCT ID: NCT02856412

Study Application (Version 1.30)

1.0 General Information

***Enter the full title of your study:**

Improving Mind/Body Health and Functioning with Integrative Exercise

***Enter the study alias:**

VGX RR&D
* This field allows you to enter an abbreviated version of the Study Title to quickly identify this study.

2.0 Add departments

2.1 and Specify Research Location:

Is Primary?	Department Name
◆	**UCSF** - *133100* - M_Psychiatry

3.0 List the key study personnel: (Note: external and affiliated collaborators who are not in the UCSF directory can be identified later in the Qualifications of Key Study Personnel section at the end of the form)

3.1 *Please add a Principal Investigator for the study:

Neylan, Thomas MD

Select if applicable

☐ Department Chair ☐ Resident
☐ Fellow

If the Principal Investigator is a Fellow, the name of the Faculty Advisor must be supplied below.

3.2 If applicable, please select the Research Staff personnel

A) Additional Investigators

Boyd, Jennifer E
Other Investigator
Chesney, Margaret A, PhD
Other Investigator
Cohen, Beth, MD, MA
Other Investigator
Gasper, Warren J
Other Investigator
Mehling, Wolf MD, MD

Other Investigator
O'Donovan, Aoife PhD, PhD
Other Investigator
Palyo, Sarah A
Other Investigator
Richards, Anne
Other Investigator
Strigo, Irina PhD, PhD
Other Investigator

B) Research Support Staff

Antonetti, Victor
Clinical Research Associate
Bertenthal, Daniel S
Data Manager
Cheng, David
Technician
Garcia Guerra, Sergio R
Clinical Research Associate
Goldstein, Lizabeth
Clinical Research Associate
Hlavin, Jennifer
Study Coordinator
Mayzel, Olga
Data Manager
Metzler, Thomas J
Biostatistician
Muratore, Laura
Study Coordinator
Phan, Jordan Dominique
Study Recruiter
Shumaker, Erik
Clinical Research Associate
Stenson, Emily P
Study Coordinator
West, Anna
Clinical Research Associate
Williams, Chanda
Clinical Research Associate

3.3 *Please add a Study Contact

Hlavin, Jennifer
Muratore, Laura
Neylan, Thomas MD

The Study Contact(s) will receive all important system notifications along with the Principal Investigator. (e.g. The project contact(s) are typically either the Study Coordinator or the Principal Investigator themselves).

3.4 If applicable, please add a Faculty Advisor/Mentor:

3.5 If applicable, please select the Designated Department Approval(s)

Add the name of the individual authorized to approve and sign off on this protocol from your Department (e.g. the Department Chair or Dean).

4.0 Initial Screening Questions

Updated April 2020 - Revised Common Rule (January 2018) Compliant / COVID-19 - v94

4.1 * PROJECT SUMMARY: (REQUIRED) Give a brief overview of this project (250 words or less). Tell us what this study is about, who is being studied, and what it aims to achieve. If you have an NIH Abstract, paste it here (Click on the orange question mark to the right for more detailed instructions):

Despite the considerable efforts of the VA to improve awareness of mental health problems and access to care, many returning veterans still report substantial barriers to seeking traditional mental health care. There is a large body of evidence demonstrating that aerobic exercise effectively improves many outcomes relevant to Posttraumatic Stress Disorder (PTSD) including; anxiety, depression, insomnia, cognition, and cardiovascular disease. In addition, there is a rapidly growing evidence base showing that aerobic exercise produces an increase in the growth of new neurons (e.g., neurogenesis) and increases the volume of the hippocampus which underscores the potential value of exercise for producing broad benefits to psychological health. Recognizing the promise that exercise might hold for attracting more veterans into care and improving overall health in veterans with PTSD, a team of investigators at the San Francisco Veterans Administration Medical Center (SFVAMC) developed a treatment protocol and completed a pilot study of Integrative Exercise (Aerobic exercise and Breath Training 3 weekly sessions over 12 weeks) versus a waitlist control condition (CHR Protocol #12-09594).

Promising results from this trial have led us to the next step which is to conduct a definitive efficacy study of Integrative Exercise versus an active health education control condition: Illness Management and Recovery (IMR), also referred to as PTSD Recovery. The control condition will be matched on contact hours with treatment personnel. The goal of this revised proposal is to test if Integrative Exercise improves overall quality of life, PTSD symptoms, sleep quality, and measures of cardiovascular health in combat Veterans with chronic PTSD relative to the IMR/PTSD Recovery condition. Another goal is to test if improvements in quality of life are predicted by improvements in cardiovascular fitness as measured by exercise capacity on treadmill testing. Finally, the proposal will test if Integrative Exercise versus IMR/PTSD Recovery will produce greater improvements in additional health outcomes, including mood, subjective sleep quality, and PTSD symptoms.

4.2 * HUD DEVICE: (REQUIRED) Does this application involve a Humanitarian Use Device (HUD):

- (●) No
- ( ) Yes, and it includes a research component
- ( ) Yes, and it involves clinical care ONLY

4.3 * TYPE OF RESEARCH: (REQUIRED) Select the option that best fits your project (Click the orange question mark to the right for definitions and guidance):

- ○ Biomedical research (including medical records review, biospecimen collection and/or use, other healthcare or health outcomes related activities, research database, biospecimen bank, or recruitment registry)
- ○ Social, behavioral, educational, and/or public policy research
- ◉ Hybrid - includes aspects of BOTH types of research (check this option if your research is mainly social/behavioral but also involves specimen collection or blood draws to look at biological measures)

4.4 * SUBJECT CONTACT: (REQUIRED) Does this study involve ANY contact or interactions with participants:

- ◉ Yes (including phone, email or web contact)
- ○ No (limited to medical records review, biological specimen analysis, and/or data analysis)

4.5 * RISK LEVEL: (REQUIRED) What is your estimation of the risk level, including all screening procedures and study activities:

- ◉ Minimal risk
- ○ Greater than minimal risk

4.6 * REVIEW LEVEL: (REQUIRED) Requested review level (Click on the orange question mark to the right for definitions and guidance):

- ○ Full Committee
- ◉ Expedited
- ○ Exempt

4.7 * EXPEDITED REVIEW CATEGORIES: (REQUIRED) If you think this study qualifies for expedited review, select the regulatory categories that the research falls under: (check all that apply)

- ☐ Category 1: Research using approved drugs or devices being used for their approved indications
- ☑ Category 2: Collection of blood samples by finger stick, heel stick, ear stick, or venipuncture in certain populations and within certain amounts
- ☐ Category 3: Prospective collection of biological specimens for research purposes by noninvasive means (e.g. buccal swabs, urine, hair and nail clippings, etc.)
- ☑ Category 4: Collection of data through noninvasive, routine clinical procedures (e.g. physical sensors such as pulse oximeters, MRI, EKG, EEG, ultrasound, moderate exercise testing, etc. - no sedation, general anesthesia, x-rays or microwaves)
- ☐ Category 5: Research involving materials (data, documents, records, or specimens) that have been or will be collected solely for nonresearch purposes
- ☑ Category 6: Collection of data from voice, video, digital, or image recordings made for research purposes
- ☑ Category 7: Research on individual or group characteristics or behavior or research employing survey, interview, oral history, focus group, program evaluation, human factorsevaluation, or quality assurance methodologies

* Does the collection of blood samples meet requirements outlined by HHS Office for Human Research Protections for **Expedited Review Research Category 2**: **(REQUIRED)**

- For healthy, nonpregnant adults who weigh at least 110 pounds the amounts drawn may not exceed 550 ml in an 8 week period and collection may not occur more frequently than 2 times per week;
- From other adults and children, considering the age, weight, and health of the subjects, the collection procedure, the amount of blood to be collected, and the frequency with which it will be collected, the

amount drawn may not exceed the lesser of 50 ml or 3 ml per kg in an 8 week period and collection may not occur more frequently than 2 times per week

(•) Yes ( ) No

4.9 *** DATA/SPECIMEN ANALYSIS ONLY: (REQUIRED) Does this study ONLY involve records review and /or biospecimen analysis (do not check 'Yes' if this is a registry, research or recruitment database, or biospecimen repository):**

( ) Yes (•) No

4.10 *** CLINICAL TRIAL: (REQUIRED)**
Is this a clinical trial:

According to The World Health Organization (WHO) and the International Committee of Medical Journal Editors (ICMJE) a clinical trial is:

- Any research study that prospectively assigns human participants or groups of humans to one or more health-related interventions to evaluate the effects on health outcomes.

ICMJE requires registration of a clinical trial in a public database (such as ClinicalTrials.gov) prior to enrollment, for eventual publication of results in member biomedical journals.
Guidance: Public Law 110-85 requires that all investigators who perform an *applicable clinical trial* must ensure that the trial is registered on a government web site called **ClinicalTrials.gov**.

The FDA requires registration for 'applicable clinical trials,' defined as follows:

- For any trials of drugs and biologics: controlled clinical investigations, other than Phase 1 investigations, of a product subject to FDA regulation.
- For trials of biomedical devices: controlled trials with health outcomes of devices subject to FDA regulation, other than small feasibility studies, and pediatric post-market surveillance.

For additional information on the **ClinicalTrials.gov** registration process at UCSF and the definition of a clinical trial for purposes of registration, visit the **ClinicalTrials.gov section of the UCSF Clinical Research Resource HUB**.

(•) Yes ( ) No

Clinical Trial Registration - 'NCT' number for this trial:

NCT02856412

4.11 *** CLINICAL TRIAL PHASE: (REQUIRED) Check the applicable phase(s):**

☐ Phase 0
☐ Phase 1
☐ Phase 1/2
☐ Phase 2
☐ Phase 2/3
☑ Phase 3
☐ Phase 4
☐ Not Applicable

4.12 * INVESTIGATOR-INITIATED: (REQUIRED) Is this an investigator-initiated study:

◉ Yes ○ No

The UCSF IRB recommends use of the Virtual Regulatory Binder to manage your study.

4.13 * CORONAVIRUS RESEARCH: (REQUIRED) Does this study involve research on coronaviruses (COVID-19, SARS, MERS or other):

○ Yes ◉ No

4.15 * CANCER: (REQUIRED) Does this study involve cancer (e.g., the study involves patients with cancer or at risk for cancer, including behavioral research, epidemiological research, public policy research, specimen analysis, and chart reviews):

○ Yes ◉ No

4.16 * RADIATION EXPOSURE: (REQUIRED) Does your protocol involve any radiation exposure to patients /subjects EITHER from standard care OR for research purposes (e.g., x-rays, CT-scans, DEXA, CT-guided biopsy, radiation therapy, or nuclear medicine including PET, MUGA or bone scans):

○ Yes ◉ No

4.17 SCIENTIFIC REVIEW: If this study has undergone scientific or scholarly review, please indicate which entity performed the review (check all that apply):

☐ Cancer Center Protocol Review Committee (PRC) (Full approval is required prior to final IRB approval for cancer-related protocols.)
☐ CTSI Clinical Research Services (CRS) Advisory Committee
☐ CTSI Consultation Services
☐ Departmental scientific review
☑ Other:

* Specify **Other: (REQUIRED)**

SFVAMC

4.18 * STEM CELLS: (REQUIRED) Does this study involve human stem cells (including iPS cells and adult stem cells), gametes or embryos:

◉ No
○ Yes, and requires IRB and GESCR review
○ Yes, and requires GESCR review, but NOT IRB review

4.19 * **FINANCIAL INTERESTS: (REQUIRED) Do you or any other responsible personnel (or the spouse, registered domestic partner and/or dependent children thereof) have financial interests related to this study:**

○ Yes ◉ No

5.0 Funding

5.1 * **FEDERAL FUNDING: (REQUIRED) Is this study currently supported in whole or in part by Federal funding, *even by a subcontract*, OR has it received ANY Federal funding in the past:**

◉ Yes ○ No

5.2 * **DoD INVOLVEMENT: Is this project linked in any way to the Department of Defense (DoD): (REQUIRED)**

○ Yes ◉ No

5.3 **SPONSORS: Identify all sponsors and provide the funding details. If funding comes from a Subcontract, please list only the Prime Sponsor:**

External Sponsors:

View Details	Sponsor Name	Sponsor Type	Awardee Institution:	Contract Type:	Project Number	UCSF RAS System Award Number ("A" + 6 digits)
⊟	US Dept of Veterans Affairs	01	SF VAMC Research Office	Grant		

Sponsor Name:	US Dept of Veterans Affairs
Sponsor Type:	01
Sponsor Role:	Funding
**CFDA Number:**	
**Grant/Contract Number:**	
Awardee Institution::	SF VAMC Research Office
**Is Institution the Primary Grant Holder:**	Yes
Contract Type:	Grant
Project Number:	
UCSF RAS System Award Number ("A" + 6 digits):	
Grant Number for Studies Not Funded thru UCSF:	
Grant Title:	
PI Name:<br>(If PI is not the same as identified on the study.)	

Explain Any Significant Discrepancy:	

Other Funding Sources and Unfunded Research - Gift, Program, Departmental or other Internal Funding (check all that apply):

- ☐ Funded by gift (specify source below)
- ☐ Funded by UCSF or UC-wide program (specify source below)
- ☐ Specific departmental funding (specify source below)
- ☐ Unfunded (miscellaneous departmental funding)
- ☐ Unfunded student project

6.0 Sites, Programs, Resources, and External IRB Review

6.1 * UCSF AND AFFILIATED SITES (check all that apply): (REQUIRED)

- ☐ UCSF Benioff Children's Hospital Oakland (BCHO)
- ☐ UCSF Cancer Center Berkeley
- ☐ UCSF Cancer Center San Mateo
- ☐ UCSF China Basin clinics and facilities
- ☐ UCSF Helen Diller Family Comprehensive Cancer Center
- ☐ UCSF Langley Porter Psychiatric Institute (LPPI)
- ☐ UCSF Medical Center at Mission Bay (Benioff Children's Hospital, the Betty Irene Moore Women's Hospital, Bakar Cancer Hospital, or outpatient clinics)
- ☐ UCSF Mount Zion
- ☐ UCSF Parnassus (Moffitt-Long hospital, dental clinics or other outpatient clinics)
- ☐ UCSF Other Sites (including Laurel Heights and all the other sites outside the main hospitals and clinics)
- ☐ Fresno - UCSF Fresno OR Community Medical Center (CMC)
- ☐ Gladstone Institutes
- ☐ Institute on Aging (IOA)
- ☐ Jewish Home
- ☐ SF Dept of Public Health (DPH)
- ☑ SF VA Medical Center (SF VAMC)
- ☐ Vitalant (formerly Blood Centers of the Pacific and Blood Systems Research Institute)
- ☐ Zuckerberg San Francisco General (ZSFG)

Research involving the SF VAMC: Please thoroughly review the Working with the SF VAMC webpage and/or consult the VA Research Office (V21SFCHRPP@va.gov or (415) 221-4810 x6425) prior to submitting your application to the IRB and:

- **If this study involves both UCSF and the VA, identify who is serving as the VA PI under 'Descriptions of Study Responsibilities' in the 'Qualifications of Investigators' section at the end of this form**
- **Include the additional required VA forms in the Study Documents section of the Initial Review Submission Packet form**

6.2 LOCATIONS: At what locations will study visits and activities occur:

IMR/PTSD Recovery and Integrative Exercise Groups:

Subjects can participate in *all* assessments and treatment classes from their own homes. Informed consent, diagnostic interview/assessments, and medical screen will take place remotely and are conducted over the phone or via videoconferencing. Self-report questionnaires can be completed from the participant's own device via Qualtrics or on paper through the mail or secure messaging (e.g., Azure RMS, Myhealthevet). Neurocogntive testing will be completed remotely from the participant's own device via Millisecond Inquisit. Treatment classes will be held remotely. Integrative Exercise classes are held using videoconferencing. PTSD Recovery classes are held primarily over videoconferencing; however, if technological issues arise, participants may attend class using VANTS phone lines.

For our clinical trial, no participant will be required to complete any assessment in-person at the SFVA Medical Center; however, if circumstances permit, local participants may be given the option to complete pre-treatment, post-treatment, and 6 month follow up assessments in-person at the SFVA Medical Center (e.g, self-reports, clinical interivew, neurocognitive testing). They may also be given the option to complete the blood draw, urine screen, and FMD procedures in-person at the SFVAMC for additional compensation.

6.3 OFF-SITE PROCEDURES: Will any study procedures or tests be conducted off-site by non-UCSF personnel:

(•) Yes ( ) No

Please identify which procedures may be done off-site:

For our fully-remote clinical trial, subjects can participate in *all* assessments and treatment classes from their own homes. Informed consent, diagnostic interview/assessments, and medical screen will take place remotely and are conducted over the phone or via videoconferencing. Self-report questionnaires can be completed from the participant's own device via Qualtrics or on paper through the mail or secure messaging (e.g., Azure RMS, Myhealthevet). Neurocogntive testing will be completed remotely from the participant's own device via Millisecond Inquisit. Treatment classes will be held remotely. Integrative Exercise classes are held remotely using videoconferencing. PTSD Recovery classes are held remotely over videoconferencing; however, if technological issues arise, PTSD Recovery participants may attend class using VANTS lines.

6.4 RESEARCH PROGRAMS: Check any UCSF research programs this study is associated with:

- [ ] Cancer Center
- [ ] Center for AIDS Prevention Sciences (CAPS)
- [ ] Global Health Sciences
- [ ] Immune Tolerance Network (ITN)
- [ ] Neurosciences Clinical Research Unit (NCRU)
- [x] Osher Center
- [ ] Positive Health Program

6.5 * CTSI CRS SERVICES: (REQUIRED) Will this study be carried out at one of the UCSF Clinical Research Services (CRS) units or utilize CRS services:

(•) Yes ( ) No

The CRS budget request form can be found at: https://crs.ucsf.edu /sites/g/files/tkssra726/f/CRS%20Budget%20Request% 20Form_Final_8.14.20%20Restricted%20Version.docx . Follow the instructions on the form to submit.

6.6 * MULTI-CENTER TRIAL: (REQUIRED) Is this a multi-center or multi-site research trial:

By '**multi-center trial**' we mean a study where the protocol is developed by an lead investigator, an industry sponsor, consortium, a disease-group, etc.,and multiple sites across the nation or in different countries participate in the trial. The local sites do not have any control over the design of the protocol.

○ Yes ◉ No

6.8 OTHER SITE TYPES: Check all the other types of sites not affiliated with UCSF with which you are cooperating or collaborating on this project:

Do NOT check any boxes below if this is a multi-center clinical trial, UCSF is just one of the sites, and neither UCSF nor one of its faculty-linked affiliates (SF VAMC, Gladstone, ZSFG) are the coordinating center.

☐ Other UC Campus

☐ Other institution

☐ Other community-based site

☐ Foreign Country

☐ Sovereign Native American nation (e.g. Navajo Nation, Oglala Sioux Tribe, Havasupai, etc.)

6.14 * RELYING ON AN EXTERNAL IRB: (REQUIRED) Does this application include a request to rely on an external IRB (a central IRB (other than the NCI CIRB) or an external IRB (other UC campus, commercial, or institutional):

○ Yes ◉ No

7.0 Research Plan and Procedures

7.1 HYPOTHESIS: Describe the hypothesis or what the study hopes to prove:

Hypothesis 1a: Subjects randomized to Integrative Exercise will have a greater improvement in quality of life as measured by the WHOQOL-BREF psychological domain compared to subjects randomized to IMR/PTSD Recovery.
Secondary Hypothesis 1b: Subjects randomized to Integrative Exercise versus IMR/PTSD Recovery will have greater improvements in cardiovascular health as measured by exercise capacity on the treadmill test.
Exploratory Hypotheses: Subjects randomized to Integrative Exercise versus IMR/PTSD Recovery will have greater improvements in additional health outcomes, including mood, subjective measures of sleep quality, and brachial artery flow mediated vasodilation. These outcomes will be used to examine the internal consistency of the effects of treatment on the primary WHOQOL-BREF outcome.

Hypothesis 2a: Participants randomized to Integrative Exercise will demonstrate greater improvements in PTSD symptoms as measured by the CAPS score compared to those in the IMR/PTSD Recovery control group.

7.2 AIMS: List the specific aims:

Aim 1: To determine whether a 12-week course of Integrative Exercise therapy produces significant pre-post improvements in quality of life as measured by the World Health Organization Quality of Life (WHOQOL-BREF) and associated psychological and physical health outcomes relative to the Illness Management and Recovery (IMR)/PTSD Recovery condition.

Aim 2: To determine whether Integrative Exercise produces significant pre-post improvements in PTSD symptoms as measured by the CAPS score relative to the IMR/PTSD Recovery condition.

7.3 DESIGN: Briefly describe the study design (e.g., observational, interventional, randomized, placebo-controlled, blinded, cross-over, cross-sectional, longitudinal, pharmacokinetic, etc.):

Subjects with full PTSD will be recruited to participate in a parallel-groups trial. 104 subjects will be randomly assigned to one of two groups: a 12-week course of Integrative Exercise [IE] or 12-week Illness Management and Recovery (IMR)/PTSD Recovery class.

A stratified randomization strategy will be deployed to help ensure that the 2 conditions do not differ for subjects who are or are not engaged in current treatment for PTSD. Drs. Mehling and Boyd, who have extensive experience with delivering IE and IMR/PTSD Recovery respectively, will rate recorded sessions and independently rate adherence.

Participants will complete baseline assessments prior to randomization. Subjects will them be randomized to either the IE or IMR/PTSD Recovery classes.

Integrative Exercise treatment involves a combination of aerobic and strength training exercises as well as concentration training based on mindful breathing techniques. Subjects will exercise 3 times weekly, with each total workout being approximately 60 minutes in length.

The control condition will involve 36 hours of health education using the Illness and Management and Recovery (IMR)/PTSD Recovery program which is mandated by the Uniform Mental Health Services directive to be a component of PRRCs at each VA medical center. The IMR/PTSD Recovery control condition, sometimes referred to as Wellness Management and Recovery, is an educational curriculum focused on helping clients more effectively manage their illnesses to pursue their personal recovery goals.

After the 12-week intervention, participants will repeat the basline assessments. Measures of subjective sleep quality, PTSD symptoms, mood states – particularly depression, and quality of life (QOL), will be obtained in all subjects (Integrative Exercise and IMR/PTSD Recovery) at baseline, weeks 4 & 8, and again following the end of 12 weeks. Subjects randomized to Integrative Exercise will be asked to rate their satisfaction with the intervention (i.e., acceptability and feasibility and comparison with other PTSD treatments that participants may have experienced in the past) at the end of 12 weeks.

Subjects in both conditions will have repeat clinical assessments at 6 months to examine durability of treatment gains.

7.4 BACKGROUND AND SIGNIFICANCE: Briefly provide the background and significance of this study (e.g. why is this study needed) (space limit: one half page):

If this is a first in humans study, please summarize the safety data from the animal studies. For pediatric drug or device studies, please identify if this is the first study in pediatric populations.

Background and Significance:
Given that aerobic exercise has been found to improve brain health and neurogenesis[10], cognitive function[11], mood[12], sleep[13], and cardiovascular health[14], there is a strong rationale to determine if exercise may be an effective rehabilitative intervention for Veterans with combat related PTSD. Our group has also demonstrated that individuals with PTSD have lower rates of exercise compared to others without PTSD of the same age and sex[15], suggesting they may particularly benefit from a focus on exercise. Despite the high acceptance of exercise therapy for PTSD found in one study[16], and the considerable advantage of a treatment lacking stigma, to date there are no reported controlled trials for exercise in any population with PTSD.

7.5 PRELIMINARY STUDIES: Briefly summarize any preliminary studies relevant to your proposed research (space limit: one half page):

In our pilot study (CHR Protocol #12-09594), we enrolled a total of 46 (9 women) veteran participants. Twenty-one subjects were assigned to exercise and 25 to the wait list. Note, randomization was conducted from block randomization lists from 4 strata defined by gender and age. We had a total of 9 dropouts: 5 from the Integrative Exercise (IE) group and 4 from the Wait List (WL) group. All 5 subjects from the IE group and 2 from the WL group dropped out in the first week after randomization and were lost to follow-up. One of the subjects who dropped from WL immediately following randomization indicated they elected to enroll in a fitness program outside of the research setting. We have 38 total completers (21 WL and 16 IE).

There are strong converging lines of evidence that exercise can have positive effects on multiple functional domains relevant to PTSD: psychological and physical heath, sleep, and cardiovascular health. We have developed a novel protocol integrating the best elements of aerobics, strength training, and mindfulness-based breath training. We currently have a dedicated study team in place and with our success at recruitment and engagement, we are poised to complete a new definitive efficacy trial with an active control condition that will have sufficient power to properly evaluate whether an exercise intervention improves quality of life and broad measures of psychological and physical health in combat Veterans with PTSD. Because of the strengths of our team in PTSD-related comorbidities and clinical trials, we are in a unique position to test if exercise has positive effects on quality of life, psychological health, sleep, and cardiovascular fitness. It is our position that positive results in all of these domains would greatly enhance the attractiveness of this intervention to returning warfighters with PTSD and attract more Veterans into treatment. Further, the empiric data obtained from this project will help inform policy regarding therapeutics that serve the rehabilitation mission of RR&D.

7.6 * TREATMENT PROTOCOL: Is this a treatment study, i.e. does this study intend to provide treatment to individuals with a medical or psychological condition: (REQUIRED)

(●) Yes ( ) No

7.7 * BILLABLE PROCEDURES: Does this study involve any procedures, lab tests or imaging studies that have a CPT code and could be billable to patients, their insurance, Medi-Cal, Medicare, or any other entity (answer 'Yes' even if the study is going to pay for all the procedures): (REQUIRED)

( ) Yes (●) No

If you are not sure if your study involves billable procedures, send an email to the UCSF Office of Clinical Research (OCR) for help answering this question.

7.8 * COMMON RESEARCH ACTIVITIES: Types of research activities that will be carried out. Check all that apply and describe in more detail in the 'Procedures / Methods' section: (REQUIRED)

- [x] Interviews, questionnaires, surveys
- [x] Educational or cognitive tests

- [ ] Focus groups
- [ ] Social media-based research activities
- [ ] Observation
- [x] Fitness tests or other exertion activities
- [ ] Use of mobile health apps or other apps
- [ ] Collection of data from wearable tech such as Fitbit, Apple Watch, Garmin, motion actigraphs, etc.)
- [x] Non-invasive imaging or testing (MRI, EEG, pulse oximetry, etc.)
- [ ] Imaging procedures or treatment procedures that involve radiation (x-rays, CT scans, CT-guided biopsies, DEXA scans, MUGA or PET scan)
- [ ] Administration of contrast agent
- [ ] Randomization to one intervention versus another
- [x] Use of placebo
- [ ] Biopsy conducted solely for research purposes
- [ ] Sham surgical procedure
- [ ] None of the above

7.9 * PROCEDURES / METHODS: (REQUIRED)

Describe the research methods and study activities taking place at each site (e.g. what will participants be asked to do and what will members of the study team do?). If there will be multiple participant groups or study sites, explain what will happen with each group or study sites.

If some of the activities would occur even if the person were not in the study, as in the case of treatment or tests performed for diagnostic purposes, **clearly differentiate between those activities that will be done solely for research purposes and those that are happening as part of routine care.**

Please call our office at 415-476-1814 and ask to speak to someone on the Expedited Review team if you need help differentiating between what parts are research and what parts aren't.

Informed Consent
Written or electronic informed consent will be obtained by study personnel prior to the first diagnostic screening appointment and prior to the start of any study related procedures.

Screening and Eligibility Assessments
The following procedures will be started after informed consent is obtained:

- **Clinicial Assessment & Questionnaires:** To determine study eligibility and to obtain baseline assessment, a diagnostic interview will be conducted. A trained clinical interviewer will conduct a diagnostic assessment using the scales listed in the Clinician Administered Assessments section below. The diagnostic assessments will be audio recorded.
- **Medical Evaluation:** All potential participants will meet with the study doctor who will review their medical and surgical history as well as concurrent medications. Participants will be asked about knee symptoms and/or other relevant previous injuries that could be exacerbated by exercise. All participants will complete the Physical Activity Readiness Questionnaire (American College of Sports Medicine [ACSM guidelines] to be cleared to exercise. If needed, each subject will perform a graded exercise test (ASCM guidelines) to assess functional capacity.

Optional Screening and Eligibility Assessment

- **Blood Draw:** Subjects will NOT be required to complete a blood draw for inclusion in this 100% remote clinical trial; however, if circumstances permit, local participants may be given the option to have their blood drawn for additional compensation. Blood will be collected at the CTSI Clinical Research Center at the SF VA Medical Center. No more than 61.5 ml (or 12 teaspoons) of blood will be drawn.

- Routine lab tests: This will include a serum chemistry panel, liver function tests, thyroid functions, complete blood count, and differential, total cholesterol, HDL-cholesterol, LDL-cholesterol, triglycerides, glucose, hemoglobin A1c, a urine toxicology screen, and potentially other markers.
- Research analyses: The blood will also be used to study chemicals, enzymes, and genes related to PTSD. The blood will be stored at the SFVAMC in Dr. Thomas Neylan's -80 freezer (building 8; room 4) for future analysis.

Pre-treatment Assessments

Participants who are eligible following all Screening Assessments will be asked to complete Pre-treatment Assessments.

- **Self-Report Questionnaires:** All Questionnaires listed in the Instruments section will be completed via Qualtrics or on paper through the mail or secure email messaging (e.g., Azure RMS, Myhealthevet). If circumstances permit, participants may be given the option to complete questionnaires on-site at the SFVAMC.
- **Neurocognitive Testing**: All neurocognitive tests listed in the Instruments section will be completed remotely via Millisecond Inquisit. If circumstances permit, participants may be given the option to complete neurocognitive testing on-site at the SFVAMC.

Optional Pre-treatment Assessment

- **Brachial flow-mediated dilation (FMD) test:** Subjects will NOT be required to complete the FMD test for inclusion in this 100% remote clinical trial; however, if circumstances permit, local participants may be given the option to complete this test for additional compensation.
  - Brachial Artery Flow-Mediated Dilation will be performed by a trained vascular technician. Flow mediated dilation (FMD) will be measured with standard procedures using an occlusion cuff and doppler ultrasound. Staff will attach the occlusion cuff to the participants' upper arm, initiate supra-systolic cuff occlusion for five minutes, and then terminate occlusion and measure brachial artery response with ultrasound for 60 seconds post occlusion. Subjects will be asked to rate the blood pressure cuff on : a) its intensity (by indicating the maximum sensation of pain on a scale from –10/"Neutral" to +10/"Extremely Painful" with 0/"Pain threshold" as the midpoint); and b) its affect (on a scale from –10/"Extremely Pleasant" to +10/"Extremely Unpleasant" with 0/"Unpleasantness Threshold" as the midpoint) using a visual analogue scale [182].

  Note: we will be asking participants who take blood pressure medications (including alpha 1 blockers, nitrates, or other blood pressure medications) to hold their medications until after they complete the FMD test. They will also be instructed to fast the morning of the procedure and to avoid nicotine and caffeine at least 4 hours prior to the test. Breakfast will be provided immediately after this fasting procedure.
  Note: we will be asking female participants to report any known pregnancy.

Randomization

Participants will be randomized to either 12 weeks of Integrative Exercise or 12 weeks of IMR /PTSD Recovery. Participants will complete the Commitment to Attend Exercise/PTSD Recovery Classes prior to starting remote classes.

12-week Integrative Exercise (IE) Therapy condition: The exercise program will be 12 weeks in duration, involving a combination of aerobic and strength training exercises as well as concentration training based on mindful breathing techniques from yoga and mindfulness approaches recommended by Dr. Hoge (Hoge, 2010). Subjects will exercise 3 times weekly, with each total workout being approximately 60 minutes in length. All exercise will be supervised and documented by a trained professional in order to validate adherence and allow for program replications by others as part of an intervention program for PTSD.

The exercise program is designed with the following overall characteristics: it can be reproduced indoors as well as outdoors; it can be done in a group setting with participants at various fitness levels; it can also be performed alone; it does not require a club membership or machines; it is accessible to combat veterans of all socio-economic and educational levels; it is safe to veterans with injuries; it is sufficiently different from usual fitness center programs to be attractive to combat veterans; it can be recorded on an MP3 recorder for use in a private home.

While the overall goals of the exercises are to increase strength and cardiovascular fitness, participants will practice and refine the following exercise skills/techniques: nose breathing, mental focus/mindfulness, body scanning, rhythmic limbering, dynamic constant resistance, plyometics, and isometric resistance.

12-week Illness Management and Recovery (IMR)/PTSD Recovery condition: The control condition will involve 36 hours of health education using the Illness and Management and Recovery (IMR)/PTSD Recovery program[89] which is mandated by the Uniform Mental Health Services directive to be a component of PRRCs at each VA medical center. The IMR/PTSD Recovery control condition, sometimes referred to as Wellness Management and Recovery, is an educational curriculum focused on helping clients more effectively manage their illnesses to pursue their personal recovery goals. The IMR/PTSD Recovery workbook has detailed adherence checklists which filled out by research staff at the end of each session. The 11 modules include the following topic areas which have been adapted for use in PTSD: recovery, practical facts about PTSD, stress-vulnerability, building social support, medications for PTSD, drug and alcohol use, reducing relapse, coping with stress, coping with persistent symptoms, getting needs met in the VA healthcare system, and living a healthy lifestyle[61, 89].

4 and 8-week Self-Report Assessments
All participants will complete the following self-report assessments at 4 weeks and 8 weeks after beginning the study intervention: PTSD Checklist (PCL), Insomnia Severity Index (ISI), Pittsburgh Sleep Quality Index (PSQI), The Pittsburgh Sleep Quality Index Addendum for PTSD (PSQI-A), The Symptom Checklist-90-R (SCL-90-R), Godin Leisure-Time Exercise Questionnaire, Five Facet Mindfulness Questionnaire (FFMQ), Multidimensional Assessment of Interoceptive Awareness (MAIA), and The World Health Organization Quality of Life (WHOQOL-BREF).

Post-treatment 12-week assessments
Several of the procedures completed during Screening and Eligibility and pre-treatment will be repeated:

- **Clinician Assessment & Questionnaires:** *See above for description*
- **Self-Report Questionnaires:** *See above for description*
- **Neurocognitive Testing***: See above for description*

Optional Post-treatment 12-week assessments
If circumstances permit, local participants may be given the option to complete the below procedures for additional compensation:

- **Blood Draw:** *See above for description. The same amount of blood may be collected as at baseline: 61.5 ml (or 12 teaspoons)*
- **Brachial flow-mediated dilation (FMD) test:** *See above for description*

Post-treatment 6 month assessments:
All pariticipants will complete the following assessments 6 months after completing the study intervention:

- **Clinician Assessment & Questionnaires:** *See above for description.*
- **Self-Report Questionnaires:** *See above for description.*
- **Neurocognitive Testing***: See above for description*

7.10 STANDARD CLINICAL PRACTICE: To what extent, if any, do the planned research procedures differ from the care that people would otherwise receive at this institution or the study site if not being done locally:

Integrative Exercise is not a standard treatment for PTSD, but there is emerging evidence that it is helpful. The other condition, Illness Management and Recovery/PTSD Recovery has been used in PTSD, but also is not a standard treatment.

7.11 INSTRUMENTS: List all questionnaires, surveys, interview, or focus group guides that will be used for this study:

If the instruments are not complete or not available because they will be developed as part of this study, describe the basic content or include an outline and submit the final versions to the IRB with a modification for approval prior to use.

The following assessments will be utilized in all subjects at baseline and after the 12-week treatment period, and at the 6-month follow-up period. All of the instruments involving a clinical rater will be conducted by an evaluator who will not be conducting the treatment and will be kept blind to treatment assignment. Subjects will be instructed prior to the assessment at 12 weeks and 6 months not to disclose their treatment assignment to the interviewer.

1. The World Health Organization Quality of Life (WHOQOL-BREF)[51, 52]: The WHOQOL-BREF instrument comprises 26 items, which measure the following broad domains: physical health, psychological health, social relationships, and environment. The WHOQOL-BREF is a shorter version of the original WHOQOL-100 instrument and is more convenient for use in large research studies or clinical trials. The Psychological Domain, our primary outcome, is derived from 6 items which index body image, negative & positive feelings, self-esteem, spirituality, and cognition. Each item has 5 response options with higher scores denoting higher psychological health. The mean score of items within each domain is used to calculate the domain score. Mean scores are then multiplied by 4 in order to make transformed domain scores to a range of 4-20 comparable with the scores used in the WHOQOL-100.
2. Structured Clinical Interview for DSM-5-Research Version (SCID-5-RV,[64]). The SCID will be repeated after the 12-week treatment period.
3. Clinician Administered PTSD Scale for DSM-5 (CAPS-5,[65]). The CAPS-5 is a 30 item scale that provides both a dimensional and categorical measure of PTSD. The CAPS-5 will determine lifetime and current PTSD. The CAPS-5 items are rated with a single severity score that incorporates both frequency and intensity PTSD-related symptoms. In addition to assessing the 20 DSM-5 PTSD symptoms, questions target the onset and duration of symptoms, subjective distress, impact of symptoms on social and occupational functioning, improvement in symptoms since a previous CAPS administration, overall response validity, overall PTSD severity, and specifications for the dissociative subtype (depersonalization and derealization)[65].
4. Life Stressor Checklist-Revised (LSC-R): Structured clinical interview for lifetime exposure to stressful life events [66]. This structured clinical interview for lifetime exposure to stressful life events will be used to characterize the type of trauma exposure and age of occurrence(s) of different traumas in all subjects.
5. Addiction Severity Index Lite (ASI-Lite)[67]: The ASI-Lite Composite Score for Alcohol Use will provide a secondary measure of past month alcohol use severity, and the ASI-Lite number of years of alcohol use will provide a marker of lifetime alcohol use. The ASI-Lite Composite Score for Drug Use will provide a secondary measure of past month non-alcohol substance use severity, and the ASI-Lite number of years of drug use will provide a marker of lifetime non-alcohol substance use. The ASI-Lite is a valid and reliable standardized research interview to assess the occurrence and severity of alcohol and non-alcohol substance abuse. The ASI-Lite includes questions about the frequency, duration, and severity of problems over the subject’s lifetime and in the past 30 days.
6. Smoking status[68] is a two-question categorical measure employed by the Centers for Disease Control and Prevention National Health Interview Survey, and categorizes individuals into one of three groups: (a) "Never smokers", adults 18 or over who have smoked fewer than 100 cigarettes in their lifetime; (b) "Former smokers", adults who have smoked at least 100 cigarettes in their lifetime but are not smoking at time of interview; and (c) "Current smokers", adults who have smoked at least 100 cigarettes over their lifetime and who are still smoking at time of interview.
7. Number of pack years[68] is a two-question continuous measure of smoking that utilizes the number of cigarettes per day multiplied by the number of years of smoking to calculate pack years of smoking.
8. Five Facet Mindfulness Questionnaire (FFMQ)[69, 70]: The FFMQ is a 39-item questionnaire derived from a factor analysis of other mindfulness questionnaires. It assesses five facets of mindfulness: observing, describing, acting with awareness, non-judging and non-reactivity to inner experience which represent elements of mindfulness as it is currently conceptualized. Items are rated on a Likert scale ranging from 1 (never or very rarely true) to 5 (very often or always true). The FFMQ has been shown to have good internal consistency (alpha coefficient range .72 to .92) in several samples and significant relationships in the predicted directions with domains related to mindfulness[69, 70]. The FFMQ allows a detailed assessment of changes as a function of mindfulness and therefore will be used as a secondary outcome.
9. Godin Leisure-Time Exercise Questionnaire[71, 72]: is a validated brief inventory assessing sedentary, work, recreational, and aerobic activity in a typical week. This metric will be used to measure time spent in vigorous activity and will serve multiple purposes: a) It will be used to test if randomization effectively balances levels of baseline vigorous activity across the two groups; b) It will be used as a manipulation check to ensure that subjects randomized to IE engage in more vigorous activity after randomization than subjects

randomized to IMR/PTSD Recovery; c) It will be used as a secondary predictor of treatment response.

10. The Physical Activity Self-Efficacy scale (PASE)[73]: The PASE will be used as an exploratory measure of perceived confidence to continue exercising in the face of competing day-to-day conditions.

The following measures will be obtained in all subjects at Pre-treatment, week 4, week 8, and after 12 weeks, and at the 6 month follow-up period:

1. PTSD Checklist for DSM-5 (PCL-5)[74]. The PCL-5 is a validated self-report rating scale for assessing PTSD symptoms. It consists of 20 items that correspond to the DSM-5 symptoms of PTSD.
2. Symptom Check-List-90-Revised (SCL-90-R) [147]. The SCL-90-R is a standard self-report measure of general psychopathology. Scored for nine primary dimensions and three summary indices, the SCL-90-R manual reports extensive reliability and validity data. **Sleep and Alertness Assessment Methods:**
3. Pittsburgh Sleep Quality Index (PSQI)[75]. This self-report measure provides a subjective assessment of sleep quality, sleep latency, sleep duration, sleep efficiency, sleep disturbances (including nightmares), use of sedative-hypnotics, and daytime energy. This index is now widely used and has been validated by polysomnography. The PSQI will be our main measure of sleep quality.
4. PSQI- PTSD Addendum (PSQI-A)[76]. The PSQI-A assesses disruptive nocturnal behaviors related to PTSD, such as hot flashes, nightmares, and episodes of terror during sleep. The total score ranges from 0 (normal) to 21 (severe). The PSQI-A has demonstrated good internal consistency and convergent validity. The PSQI-A will be secondary measure of sleep quality that will be used in exploratory analyses.
5. Insomnia Severity Index (ISI): The ISI is a valid and reliable self-report measure[77] that is a more specific index of perceived insomnia severity, as compared to the widely used PSQI measure which captures sleep disturbances of all types. The internal consistency of the ISI was found to be excellent (Cronbach's = 0.74) and has been validated with both sleep diary and polysomnography[77]. The ISI will be secondary measure of sleep quality that will be used in exploratory analyses.

The following neurocognitive tests will be obtained via Millisecond Inquisit in all subjects at the Pre-treatment, Post-treatment, and 6 month follow-up period:

1. Short Arrow Flanker Test
2. List Learning Test
3. Letter number sequence Test

If circumstances permit and a subject elects to complete neurocognitive tests on-site at the SFVAMC, subjects willl complete the following neurocognitive tests:

1. Flanker Inhibitory Control and Attention Test
2. Oral Symbol Digit Test
3. Rey Auditory Verbal Learning Test (Rey AVLT)

Attach any unpublished instruments in the 'Other Study Documents' section of the Initial Review Submission Packet form after completing the study application. Published instruments should NOT be attached.

7.12 * BIOSPECIMEN COLLECTION: Are you drawing any blood or collecting other biosamples (e.g. tissue, buccal swabs, urine, saliva, hair, etc.) for analysis under this protocol and/or storage for future research: (REQUIRED)

(•) Yes ( ) No

* Could this study generate genetic data that may be broadly shared (e.g., submitted to NIH in compliance with **Genomic Data Sharing (GDS)/Genome-Wide Association Studies (GWAS)** requirements): **(REQUIRED)**

( ) Yes (•) No

Please check the Resource Sharing Plan section of your funding notice. You will not be able to share the data as required by your funding agency if the consent form doesn't include the required language.

7.13 STATISTICAL METHODS: Briefly summarize the methods and types of analyses that will be performed:

Study groups will be described in terms of baseline characteristics (including demographic and clinical measures, and baseline levels of the outcome variables) using appropriate summary statistics. Distributions of clinical measures and outcomes will be examined for presence of outliers and need for outcome transformations. The primary analyses will be *unadjusted* intent-to-treat analyses, with all subjects randomized included in the analyses. *Only the stratification factor, concurrent PTSD treatment status, will be included as a covariate in the primary analysis. Other baseline factors that remain unbalanced after randomization and are related to the outcomes will be analyzed in a separate sensitivity analysis to assess the robustness of the primary analysis*. All available time point data from any dropouts will be included in the analyses, using mixed models to accommodate the missing data. Residuals will be examined to ensure model assumptions are met. If we find violation of distributional assumptions, we will consider (1) transformations of or alternative distributions for the outcomes, and (2) bootstrapped 95% CI on the estimated intervention effect. If outliers are found, we will determine if data errors were overlooked during cleaning and check their influence via removal. The frequency and timing at which outcomes are obtained varies by measure. Some key measures, including the CAPS and the Exercise Treadmill Test, are measured at two time points, pre- and post-treatment, while self-report measures such as the WHOQOL, PCL and PSQI are measured at four time points. All of these outcomes will be analyzed with linear mixed models (LMM's). Measures with more than two measurement occasions make full use of the LMM strategy, whereas LMM's for pre-post measures reduce to ANCOVA as a special case, except for the added flexibility of modeling heterogeneous group variances. In analyses with intermediate time points, a number of modeling choices must be made, including whether to treat the time variable as continuous or categorical, the form of the within-subjects correlation matrix, and whether to allow for heterogeneity of variance across groups and/or time points. The best fitting model will be selected according to likelihood ratio tests (for nested models) or the Bayesian Information Criterion (BIC; for non-nested models) before examining any coefficients or test statistics. The LMM is an unbiased intent-to-treat analysis under assumptions of Missing At Random (MAR). *Any baseline clinical measures that correlate with dropout will be added to the models in a sensitivity analysis.* However, the possibility of informative missingness (i.e., missingness related to the missing outcome or other unmeasured variables) cannot be tested but also cannot be ruled out. Therefore, besides our primary LMM analytic strategy, we propose another sensitivity analysis for departure from MAR, based on copy reference (CR) imputation[90, 91]. In this procedure, post-dropout data in the treatment group are multiply imputed from a model that assumes that treatment arm dropouts revert to the control group trajectory at a rate determined by the within-control group correlation structure. In the limiting case of participants who drop out of the treatment arm immediately after baseline, their imputed trajectory follows that of the control group. Dropouts from the control group are imputed from the control group only model based on baseline data and any observed time points before dropout. Multiply-imputed data sets are analyzed with the LMMs as proposed, with the estimates combined using Rubin's rules. The effect is to reduce the estimated difference between groups at end of trial, and yields a more conservative effect estimate than the LMM without imputation.

7.14 REFERENCES: List only the 5-10 most relevant references (a separate bibliography can be attached for reference purposes if this study involves novel approaches, agents, or an emerging technology that the IRB may not be familiar with):

REFERENCES CITED

1. Kraemer, H.C. A Source of False Findings in Published Research Studies: Adjusting for Covariates. *JAMA psychiatry* **72**, 961-962 (2015).
2. Kraemer, H.C., Frank, E. & Kupfer, D.J. Moderators of treatment outcomes: clinical, research, and policy importance. *JAMA* **296**, 1286-1289 (2006).
3. Rothbaum, B.O. *et al.* A Randomized, double-blind evaluation of D-cycloserine or alprazolam combined with virtual reality exposure therapy for posttraumatic stress disorder in Iraq and Afghanistan war veterans. *Am J Psychiatry* **171**, 640-648 (2014).

4. Imel, Z.E., Laska, K., Jakupcak, M. & Simpson, T.L. Meta-analysis of dropout in treatments for posttraumatic stress disorder. *J Consult Clin Psychol* **81**, 394-404 (2013).
5. Mueser, K.T. *et al.* The Trauma Recovery Group: a cognitive-behavioral program for post-traumatic stress disorder in persons with severe mental illness. *Community mental health journal* **43**, 281-304 (2007).
6. McGuire, A.B. *et al.* Factors Affecting Implementation of an Evidence-Based Practice in the Veterans Health Administration: Illness Management and Recovery. *Psychi atr Rehabil J* (2015).
7. Hay-Smith, E.J., McClurg, D., Frawley, H. & Dean, S.G. Exercise adherence: integrating theory, evidence and behaviour change techniques. *Physiotherapy* (2015).
8. Hoge, C.W. *et al.* Combat duty in Iraq and Afghanistan, mental health problems, and barriers to care. *The New England journal of medicine* **351**, 13-22 (2004).
9. Seal, K.H. *et al.* VA mental health services utilization in Iraq and Afghanistan veterans in the first year of receiving new mental health diagnoses. *J Trauma Stress* **23**, 5-16 (2010).
10. Meerlo, P., Mistlberger, R.E., Jacobs, B.L., Craig Heller, H. & McGinty, D. New neurons in the adult brain: The role of sleep and consequences of sleep loss. *Sleep Med Rev* **13**, 187-194 (2009).
11. Liu-Ambrose, T. *et al.* Resistance training and executive functions: a 12-month randomized controlled trial. *Arch Intern Med* **170**, 170-178 (2010).
12. Babyak, M. *et al.* Exercise treatment for major depression: maintenance of therapeutic benefit at 10 months. *Psychosom Med* **62**, 633-638 (2000).
13. Yang, P.Y., Ho, K.H., Chen, H.C. & Chien, M.Y. Exercise training improves sleep quality in middle-aged and older adults with sleep problems: a systematic review. *Journal of physiotherapy* **58**, 157-163 (2012).
14. Thompson, P.D. *et al.* Exercise and physical activity in the prevention and treatment of atherosclerotic cardiovascular disease: a statement from the Council on Clinical Cardiology (Subcommittee on Exercise, Rehabilitation, and Prevention) and the Council on Nutrition, Physical Activity, and Metabolism (Subcommittee on Physical Activity). *Circulation* **107**, 3109-3116 (2003).
15. Zen, A.L., Whooley, M.A., Zhao, S. & Cohen, B.E. Post-traumatic stress disorder is associated with poor health behaviors: Findings from the Heart and Soul Study. *Health Psychol* **31**, 194-201 (2012).
16. Otter, L. & Currie, J. A long time getting home: Vietnam Veterans' experiences in a community exercise rehabilitation programme. *Disabil Rehabil* **26**, 27-34 (2004).
17. Newman, C.L. & Motta, R.W. The effects of aerobic exercise on childhood PTSD, anxiety, and depression. *International journal of emergency mental health* **9**, 133-158 (2007).
18. Diaz, A.B. & Motta, R. The effects of an aerobic exercise program on posttraumatic stress disorder symptom severity in adolescents. *International journal of emergency mental health* **10**, 49-59 (2008).
19. Manger, T.A. & Motta, R.W. The impact of an exercise program on posttraumatic stress disorder, anxiety, and depression. *International journal of emergency mental health* **7**, 49-57 (2005).
20. Kim, S.H. *et al.* PTSD symptom reduction with mindfulness-based stretching and deep breathing exercise: randomized controlled clinical trial of efficacy. *J Clin Endocrinol Metab* **98**, 2984-2992 (2013).
21. Rethorst, C.D. & Trivedi, M.H. Evidence-based recommendations for the prescription of exercise for major depressive disorder. *J Psychiatr Pract* **19**, 204-212 (2013).
22. Cooney, G.M. *et al.* Exercise for depression. *Cochrane database of systematic reviews (Online)* **9**, CD004366 (2013).
23. Wipfli, B.M., Rethorst, C.D. & Landers, D.M. The anxiolytic effects of exercise: a meta-analysis of randomized trials and dose-response analysis. *J Sport Exerc Psychol* **30**, 392-410 (2008).
24. Steptoe, A., Edwards, S., Moses, J. & Mathews, A. The effects of exercise training on mood and perceived coping ability in anxious adults from the general population. *J Psychosom Res* **33**, 537-547 (1989).
25. Larun, L., Nordheim, L.V., Ekeland, E., Hagen, K.B. & Heian, F. Exercise in prevention and treatment of anxiety and depression among children and young people. *C ochrane database of systematic reviews (Online)* **3**, CD004691 (2006).
26. Driver, H.S. & Taylor, S.R. Exercise and sleep. *Sleep Med Rev* **4**, 387-402 (2000).
27. Roszell, D.K., McFall, M.E. & Malas, K.L. Frequency of symptoms and concurrent psychiatric disorder in Vietnam veterans with chronic PTSD. *Hospital & Community Psychiatry* **42**, 293-296 (1991).
28. Kessler, R.C., Sonnega, A., Bromet, E., Hughes, M. & Nelson, C.B. Posttraumatic stress disorder in the National Comorbidity Survey. *Archives of General Psychiatry* **52**, 1048-1060 (1995).

29. Mellman, T.A., Clark, R.E. & Peacock, W.J. Prescribing patterns for patients with posttraumatic stress disorder. *Psychiatr Serv* **54**, 1618-1621 (2003).
30. Friedman, M.J. Future pharmacotherapy for post-traumatic stress disorder: prevention and treatment. *Psychiatr Clin North Am* **25**, 427-441 (2002).
31. Boscarino, J.A. Posttraumatic stress disorder and mortality among U.S. Army veterans 30 years after military service. *Annals of epidemiology* **16**, 248-256 (2006).
32. Boscarino, J.A. Diseases among men 20 years after exposure to severe stress: implications for clinical research and medical care [see comments]. *Psychosomatic Medicine* **59**, 605-614 (1997).
33. Cohen, B.E. *et al.* Posttraumatic stress disorder and health-related quality of life in patients with coronary heart disease: findings from the Heart and Soul Study. *Arch Gen Psychiatry* **66**, 1214-1220 (2009).
34. Kubzansky, L., Koenen, K., Spiro III, A., Vokonas, P. & Sparrow, D. Prospective study of posttraumatic stress disorder symptoms and coronary heart disease in the Normative Aging Study. *Archives of general psychiatry* **64**, 109 (2007).
35. Kubzansky, L.D., Koenen, K.C., Jones, C. & Eaton, W.W. A prospective study of posttraumatic stress disorder symptoms and coronary heart disease in women. *Health Psychol* **28**, 125-130 (2009).
36. Jordan, H.T. *et al.* Cardiovascular disease hospitalizations in relation to exposure to the September 11, 2001 World Trade Center disaster and posttraumatic stress disorder. *Journal of the American Heart Association* **2**, e000431 (2013).
37. Vaccarino, V. *et al.* Posttraumatic Stress Disorder and Incidence of Coronary Heart Disease: A Twin Study. *Journal of the American College of Cardiology* (2013).
38. Ahmadi, N. *et al.* Post-traumatic stress disorder, coronary atherosclerosis, and mortality. *Am J Cardiol* **108**, 29-33 (2011).
39. Turner, J.H., Neylan, T.C., Schiller, N.B., Li, Y. & Cohen, B.E. Objective evidence of myocardial ischemia in patients with posttraumatic stress disorder. *Biol Psychiatry* **74**, 861-866 (2013).
40. Hsu, S. *et al.* A clinician's guide to the ABCs of cardiovascular disease prevention: the Johns Hopkins Ciccarone Center for the Prevention of Heart Disease and American College of Cardiology Cardiosource Approach to the Million Hearts Initiative. *Clin ical cardiology* **36**, 383-393 (2013).
41. Libby, P., Aikawa, M. & Jain, M.K. Vascular endothelium and atherosclerosis. *Han dbook of experimental pharmacology*, 285-306 (2006).
42. Greenland, P. *et al.* 2010 ACCF/AHA guideline for assessment of cardiovascular risk in asymptomatic adults: a report of the American College of Cardiology Foundation /American Heart Association Task Force on Practice Guidelines. *Journal of the American College of Cardiology* **56**, e50-103 (2010).
43. Yeboah, J. *et al.* Predictive value of brachial flow-mediated dilation for incident cardiovascular events in a population-based study: the multi-ethnic study of atherosclerosis. *Circulation* **120**, 502-509 (2009).
44. Ganz, P. & Vita, J.A. Testing endothelial vasomotor function: nitric oxide, a multipotent molecule. *Circulation* **108**, 2049-2053 (2003).
45. Crawford, C. *et al.* A Systematic Review of Biopsychosocial Training Programs for the Self-Management of Emotional Stress: Potential Applications for the Military. *Evide nce-based complementary and alternative medicine : eCAM* **2013**, 747694 (2013).
46. Kearney, D.J., McDermott, K., Malte, C., Martinez, M. & Simpson, T.L. Effects of participation in a mindfulness program for veterans with posttraumatic stress disorder: a randomized controlled pilot study. *J Clin Psychol* **69**, 14-27 (2013).
47. Polusny, M.A. *et al.* Mindfulness-Based Stress Reduction for Posttraumatic Stress Disorder Among Veterans: A Randomized Clinical Trial. *JAMA* **314**, 456-465 (2015).
48. Steenkamp, M.M., Litz, B.T., Hoge, C.W. & Marmar, C.R. Psychotherapy for Military-Related PTSD: A Review of Randomized Clinical Trials. *JAMA* **314**, 489-500 (2015).
49. Brown, R.P., Gerbarg, P.L. & Muench, F. Breathing practices for treatment of psychiatric and stress-related medical conditions. *Psychiatr Clin North Am* **36**, 121-140 (2013).
50. Blake, D.D. *et al.* The development of a clinician-administered PTSD scale. *Journ al of Traumatic Stress* **8**, 75-90 (1995).
51. Development of the World Health Organization WHOQOL-BREF quality of life assessment. The WHOQOL Group. *Psychol Med* **28**, 551-558 (1998).
52. Skevington, S.M., Lotfy, M., O'Connell, K.A. & Group, W. The World Health Organization's WHOQOL-BREF quality of life assessment: psychometric properties and results of the international field trial. A report from the WHOQOL group. *Qual Life Res* **13**, 299-310 (2004).
53. Gehi, A.K., Ali, S., Na, B., Schiller, N.B. & Whooley, M.A. Inducible ischemia and the risk of recurrent cardiovascular events in outpatients with stable coronary heart disease: the heart and soul study. *Arch Intern Med* **168**, 1423-1428 (2008).

54. Heinz, A. *et al.* Effects of acute psychological stress on adhesion molecules, interleukins and sex hormones: implications for coronary heart disease. *Psychopharmacology (Berl)* **165**, 111-117 (2003).
55. Spieker, L.E. *et al.* Mental stress induces prolonged endothelial dysfunction via endothelin-A receptors. *Circulation* **105**, 2817-2820 (2002).
56. Ghiadoni, L. *et al.* Mental stress induces transient endothelial dysfunction in humans. *Circulation* **102**, 2473-2478 (2000).
57. von Kanel, R. *et al.* Measures of endothelial dysfunction in plasma of patients with posttraumatic stress disorder. *Psychiatry Res* **158**, 363-373 (2008).
58. Violanti, J.M. *et al.* Posttraumatic stress symptoms and subclinical cardiovascular disease in police officers. *International Journal of Stress Management* **13**, 541-554. (2006).
59. Ras, R.T., Streppel, M.T., Draijer, R. & Zock, P.L. Flow-mediated dilation and cardiovascular risk prediction: a systematic review with meta-analysis. *Int J Cardiol* **168**, 344-351 (2013).
60. Xu, Y. *et al.* Non-invasive endothelial function testing and the risk of adverse outcomes: a systematic review and meta-analysis. *Eur Heart J Cardiovasc Imaging* **15**, 736-746 (2014).
61. McGuire, A.B. *et al.* Illness management and recovery: a review of the literature. *Psychiatr Serv* **65**, 171-179 (2014).
62. Mohr, D.C. *et al.* Treatment adherence and patient retention in the first year of a Phase-III clinical trial for the treatment of multiple sclerosis. *Mult Scler* **5**, 192-197 (1999).
63. Mosher, W.D. Design and operation of the 1995 National Survey of Family Growth. *Fam Plann Perspect* **30**, 43-46 (1998).
64. First, M.B., Williams, J.B.W., Karg, R.S. & Spitzer, R.L. *Structured Clinical Interview for DSM-5—Research Version (SCID-5 for DSM-5, Research Version; SCID-5-RV, Version 1.0.0)*. (American Psychiatric Association, Arlington, VA; 2015).
65. Weathers, F.W. *et al.* (2013).
66. Wolfe, J., Kimerling, R., Brown, P.J., Chresman, K.R. & Levin, K. *Psychometric review of the life stressor checklist-revised*, Vol. 149. (Sidran Press, Lutherville, MD; 1996).
67. McLellan, A.T. *et al.* The Fifth Edition of the Addiction Severity Index. *J Subst Abuse Treat* **9**, 199-213 (1992).
68. Schoenborn, C.A., Adams, P.F. & Schiller, J.S. Summary health statistics for the U.S. population: National Health Interview Survey, 2000. *Vital and health statistics*, 1-83 (2003).
69. Baer, R.A., Smith, G.T., Hopkins, J., Krietemeyer, J. & Toney, L. Using self-report assessment methods to explore facets of mindfulness. *Assessment* **13**, 27-45 (2006).
70. Baer, R.A. *et al.* Construct validity of the five facet mindfulness questionnaire in meditating and nonmeditating samples. *Assessment* **15**, 329-342 (2008).
71. Godin, G. & Shephard, R.J. A simple method to assess exercise behavior in the community. *Canadian journal of applied sport sciences* **10**, 141-146 (1985).
72. Gionet, N.J. & Godin, G. Self-reported exercise behavior of employees: a validity study. *Journal of occupational medicine. : official publication of the Industrial Medical Association* **31**, 969-973 (1989).
73. Marcus, B.H., Selby, V.C., Niaura, R.S. & Rossi, J.S. Self-efficacy and the stages of exercise behavior change. *Research quarterly for exercise and sport* **63**, 60-66 (1992).
74. Weathers *et al.* (2013).
75. Buysse, D.J., Reynolds, C.F., Monk, T.H., Berman, S.R. & Kupfer, D.J. The Pittsburgh Sleep Quality Index: A new instrument for psychiatric practice and research. *Psychiatry Research* **28**, 193-213 (1989).
76. Germain, A., Hall, M., Krakow, B., Katherine Shear, M. & Buysse, D.J. A brief sleep scale for Posttraumatic Stress Disorder: Pittsburgh Sleep Quality Index Addendum for PTSD. *J Anxiety Disord* **19**, 233-244 (2005).
77. Bastien, C.H., Vallieres, A. & Morin, C.M. Validation of the Insomnia Severity Index as an outcome measure for insomnia research. *Sleep Medicine* **2**, 297-307 (2001).
78. Corretti, M.C. *et al.* Guidelines for the ultrasound assessment of endothelial-dependent flow-mediated vasodilation of the brachial artery: a report of the International Brachial Artery Reactivity Task Force. *Journal of the American College of Cardiology* **39**, 257-265 (2002).
79. Pyke, K.E., Dwyer, E.M. & Tschakovsky, M.E. Impact of controlling shear rate on flow-mediated dilation responses in the brachial artery of humans. *J Appl Physiol (1985)* **97**, 499-508 (2004).
80. Mitchell, G.F. *et al.* Local shear stress and brachial artery flow-mediated dilation: the Framingham Heart Study. *Hypertension* **44**, 134-139 (2004).
81. Hoge, C.E. *Once a warrior, always a warrior.* (Globe Pequote Press, Guilford, CT; 2010).

82. American College of Sports Medicine Position Stand. The recommended quantity and quality of exercise for developing and maintaining cardiorespiratory and muscular fitness, and flexibility in healthy adults. *Med Sci Sports Exerc* **30**, 975-991 (1998).
83. American College of Sports Medicine position stand. Progression models in resistance training for healthy adults. *Med Sci Sports Exerc* **41**, 687-708 (2009).
84. Kraemer, W.J. & Fleck, S.J. *Optimizing Strength Training: Designing Nonlinear Periodization Workouts*. (Human Kinetics Publishers, Champagne, IL; 2007).
85. Fleck, S.J. & Kraemer, W.J. *Designing Resistance Training Programs*, Edn. 3rd. (Human Kinetic Publishers, Champagne, IL; 2004).
86. Kabat-Zinn, J. *Full catastrophe living. Using the wisdom of the body and mind to face stress, pain, and illness.* (Delta/Random House, New York, NY; 1990).
87. Arch, J.J. & Craske, M.G. Mechanisms of mindfulness: emotion regulation following a focused breathing induction. *Behav Res Ther* **44**, 1849-1858 (2006).
88. Miller, J.J., Fletcher, K. & Kabat-Zinn, J. Three-year follow-up and clinical implications of a mindfulness meditation-based stress reduction intervention in the treatment of anxiety disorders. *Gen Hosp Psychiatry* **17**, 192-200 (1995).
89. Mueser, K.T. *et al.* The Illness Management and Recovery program: rationale, development, and preliminary findings. *Schizophrenia bulletin* **32 Suppl 1**, S32-43 (2006).
90. Carpenter, J.R., Roger, J.H. & Kenward, M.G. Analysis of longitudinal trials with protocol deviation: a framework for relevant, accessible assumptions, and inference via multiple imputation. *Journal of biopharmaceutical statistics* **23**, 1352-1371 (2013).
91. Mallinckrodt, C. Missing Data in Clinical Trials, in *Handbook of Missing Data Methodology*. (eds. G. Molenberghs, G. Fitzmaurice, M.G. Kenward, A. Tsiatis & G. Verbeke) (CRC Press, Boca Raton, FL; 2014).
92. Westfall, P.H. & Young, S.S. (John Wiley & Sons, Inc., New York; 1993).
93. Kraemer, H.C., Mintz, J., Noda, A., Tinklenberg, J. & Yesavage, J.A. Caution regarding the use of pilot studies to guide power calculations for study proposals. *Arch Gen Psychiatry* **63**, 484-489 (2006).
94. Vittinghoff, E., Sen, S. & McCulloch, C.E. Sample size calculations for evaluating mediation. *Stat Med* **28**, 541-557 (2009).

8.0 Biospecimen Collection and/or Bank Administration

8.1 * TYPE OF SPECIMENS (check all that apply): (REQUIRED)

- ☑ Blood (provide amount below)
- ☐ Tissue (describe below)
- ☐ Other type of biospecimen, such as sputum, cerebrospinal fluid, buccal swabs, etc. (describe below)
- ☐ Existing/archival materials (name source below)

Briefly describe the types of biospecimens that will be collected. Provide the amount of blood, if applicable. For leftover/existing/archival material, identify the source:

Participants may be asked to provide both urine and blood samples. Approximately 61.5 ccs (around 12 teaspoons) of blood will be drawn. The blood will be stored at the San Francisco VA Medical Center.

8.3 * SPECIMENS ARE: (check all that apply): (REQUIRED)

- ☐ Leftover specimens from a clinical diagnostic or therapeutic procedure
- ☑ Specimens collected for research purposes only (including extra samples taken during a clinical procedure)
- ☐ Other

8.4 * FUTURE SPECIMEN USE: Will any specimens or portions of specimens be retained after the study is over for possible use in future research studies: (REQUIRED)

(•) Yes ( ) No

8.5 * SPECIMEN BANKING - CONSENT METHOD: Consent for retaining specimens for future research studies will be obtained via (check all that apply): (REQUIRED)

- [x] Specimen section within a main research study consent form
- [ ] Separate specimen consent form
- [ ] UCSF surgical consent form with tissue donation brochure

8.6 * SPECIMEN DESTINATION: Indicate where specimens will ultimately be stored: (REQUIRED)

Outside Entities: Indicate where specimens will be sent if they will not remain at UCSF (choose at least one; check all that apply):

- [ ] Cooperative group bank
- [ ] NIH
- [ ] Other university or collaborator
- [ ] Industry sponsor
- [x] Other
- [ ] N/A - all specimens will remain at UCSF

Specify to what institution, cooperative group, or company specimens will be transferred:

Blood will be stored at the SFVAMC in Dr. Thomas Neylan's -80 freezer (building 8; room 4) for future analysis.

Internal Storage: If specimens will remain at UCSF, in what kind of facility will they reside (choose at least one; check all that apply):

- [ ] UCSF repository/bank being established under this protocol
- [ ] Existing UCSF specimen repository/bank with IRB approval
- [ ] National cooperative group bank housed at UCSF
- [ ] Other location at UCSF (please describe)
- [x] N/A - no specimens will be retained at UCSF facilities

8.7 SPECIMENS SENT OUTSIDE UCSF - IDENTIFIABILITY: Will direct identifiers be associated with specimens or shared with other researchers and/or outside entities:

( ) Yes
( ) No
(•) N/A - Specimens will not be shared with others

9.0 Drugs and Devices

9.1 * DRUGS AND/OR BIOLOGICS: Are you STUDYING any drugs and/or biologics that are either approved or unapproved: (REQUIRED)

( ) Yes (•) No

If you have questions about FDA requirements for drug or device research, you can send an email to request a consult.

Note: This question is frequently answered incorrectly. **If any drugs or biologics, approved or unapproved, are being administered under this protocol, you should check 'Yes' unless you are *absolutely* sure that NONE of the drugs are part of the research protocol. Tip: Ask the PI or the sponsor if you are not sure how to answer this question.**

9.3 * MEDICAL DEVICES: Are you STUDYING any medical devices, in vitro diagnostics, or assays that are either approved or unapproved:(REQUIRED)

○ Yes ◉ No

If you have questions about FDA requirements for drug or device research, you can send an email to request a consult.

10.0 Sample Size and Eligibility Criteria

10.1 ENROLLMENT TARGET: How many people will you enroll:

104

If there are multiple participant groups, indicate how many people will be in each group:

104 subjects will be randomized so that approximately 52 will be assigned to each of the 2 study groups: IMR/PTSD Recovery and Integrative Exercise.

We estimate that we will have to consent 208 individuals to undergo the initial screening and eligibility assessment, in order to enroll 104 eligible subjects.

10.3 SAMPLE SIZE JUSTIFICATION: Explain how and why the number of people was chosen. For multi-site studies, this is referring to the number that will be enrolled across all sites:

Power Analysis: Our pilot data on WHOQOL-BREF, PTSD symptoms and METS units in response to exercise treatment are promising, but we are reluctant to base our power calculations entirely on effect sizes derived from pilot data, as these tend to be far too imprecise [93]. Rather we have powered the study to detect clinically meaningful effects on our primary variables. We conducted our power calculations for measures obtained at only pre- and post-treatment time points, because these are key outcomes and because the two time point case represents a lower bound for power when outcomes are measured more frequently. We used simulation (2000 replications each at multiple effect sizes) under the LMM to estimate power. Because our interest is in between-group differences in treatment outcomes, it is both appropriate and intuitive to express effect sizes in terms of standardized group differences at outcome. These are calculated from LMM-based marginal means and variances of post-treatment outcomes adjusted for baseline outcome measures. In the special cases where the LMM reduces to classical ANOVA-type models, these effect size estimates are identical to Cohen’s d, and they have the same interpretation in more complex models. Our proposed total sample size of 80 completers yields power of 80% at alpha = .05 to detect standardized effects (model-estimated differences between the two groups at Week 12) of d = .5, assuming within-participant correlations of .7, which is a somewhat conservative correlation compared to our pilot data (r = .8) and previous experience in our lab. Using standard deviation estimates from similar patient populations studied in our lab, we estimate that an effect size of d = .5 translates to a difference in CAPS scores of approximately 8 points, WHOQOL scores of 1.3 points, and METS scores of 2.7 units, which we believe are clinically meaningful effects and which are somewhat smaller than observed in our pilot data. There are no existing effect size estimates for calculating power for the mediation hypothesis, Hyp. 1c, and our pilot data sample is too small to provide reasonable estimates of mediation effects, as these depend on the relationship among several coefficients each of which is estimated with considerable error in a small sample. However, taking the point estimates of our pilot treatment effects on WHOQOL and CAPS at face value, we will have 80% power to detect a mediation effect of approximately 40% of the total effect of treatment on WHOQOL and 22% of

the total effect on CAPS scores, using the method of Vittinghoff, Senn, and McCullough[94]. The mediation effects estimated from our pilot data are approximately 18% for both WHOQOL and CAPS, but with 95% C.I.'s ranging from -25% (i.e., an effect in the wrong direction) to more than 60%, so these estimates provide little information. Still, our proposed sample size will be able to detect a fairly substantial mediation effect if it exists, and a smaller effect is not likely to be clinically important.

10.4 * PARTICIPANT AGE RANGE: Eligible age ranges: (REQUIRED)

- [ ] 0-6 years
- [ ] 7-12 years
- [ ] 13-17 years
- [x] 18-64 years
- [x] 65+

10.5 * STUDY POPULATIONS: Data will be collected from or about the following types of people (check all that apply): (REQUIRED)

- [ ] Inpatients
- [x] Outpatients
- [ ] Family members or caregivers
- [ ] Providers
- [x] People who have a condition but who are not being seen as patients
- [ ] Healthy volunteers
- [ ] Students
- [ ] Staff of UCSF or affiliated institutions
- [ ] None of the above

10.6 * SPECIAL SUBJECT GROUPS: Check the populations that may be enrolled: (REQUIRED)

- [ ] Children / Minors
- [ ] Adult subjects unable to consent for themselves
- [ ] Adult subjects unable to consent for themselves (emergency setting)
- [ ] Subjects with diminished capacity to consent
- [ ] Subjects unable to read, speak or understand English
- [ ] Pregnant women
- [ ] Fetuses
- [ ] Neonates
- [ ] Prisoners
- [ ] Economically or educationally disadvantaged persons
- [x] None of the above

10.7 INCLUSION CRITERIA: Briefly describe the population(s) that will be involved in this study. Include anyone that data will be collected from or about (e.g. patients, healthy controls, caregivers, providers, administrators, students, parents, family members, etc.):

Inclusion Criteria:

1. Male and Female veterans between the ages of 18-75 who are physically able to participate in an exercise program.
2. Meet criteria for of at least 3 months duration, as indexed by the Clinician Administered PTSD Scale for DSM-5 (CAPS-5). Participants may either meet full criteria as determined by the CAPS-5 or receive a score of equal to or greater than 23.

10.8 EXCLUSION CRITERIA: List any exclusion criteria (e.g. reasons why someone would not be included in the study):

Exclusion Criteria:

1. History of any psychiatric disorder with active psychosis or mania in the past 5 years.
2. Severe drug or alcohol use disorder within the past 6 months as assessed by the Structured Clinical Interview for DSM-5. Participants with moderate drug or alcohol use disorder will be reviewed on a case-by-case basis.
3. Prominent suicidal or homicidal ideation.
4. Currently exposed to recurrent trauma or have been exposed to a traumatic event within the past 3 months.
5. Pregnant, have a clinically significant neurologic disorder, systemic illness affecting CNS function, and/or physical disabilities making it impossible to use exercise equipment.
6. History of seizures in the past 5 years.
7. Any acute coronary event (i.e., Myocardial Infarction) in the past 6 months.
8. Moderate to severe Traumatic Brain Injury (any history of head trauma associated with the onset of persistent cognitive complaints, neurological symptoms, or loss of consciousness > 30 minutes).
9. Subjects who, in the opinion of the investigator, are otherwise unsuitable for a study of this type.

10. Subjects who do not have access to an electronic device that will support telehealth participation (e.g., smart phone, laptop, desktop computer).

11. Subjects who do not have private space in their residence to participate in telehealth classes.

We will not exclude patients with PTSD who are currently receiving individual or group therapy or patients who are currently taking antidepressant or anti-anxiety medication, but will apply the following criteria: patients must have been in treatment for at least 2 months, meet symptomatic criteria for inclusion, and do not have plans to discontinue treatment during the course of the trial.

10.9 * RESEARCH CONDUCTED ON PATIENT CARE WARDS: Do any study activities take place on any patient care units including inpatient wards, peri- or post-operative care units, operating rooms, or in the Emergency Department at UCSF Health medical facilities: (REQUIRED)

○ Yes ◉ No

10.11 * EMERGENCY DEPARTMENT: Does your protocol or study involve any of the following patient related activities in the emergency department (e.g. subject identification, recruitment, consent, blood draws, specimen retrieval, involvement of ED staff (nursing, tech, and/or physician), or any other ED based procedures): (REQUIRED)

○ Yes ◉ No

11.0 Recruitment and Consent

11.1 * COMPETITIVE ENROLLMENT: Is this a competitive enrollment clinical trial? By competitive enrollment, we mean that sites who do not enroll participants early may not get to participate at all: (REQUIRED)

○ Yes ◉ No

11.2 * SUBJECT IDENTIFICATION METHODS: What kinds of methods will be used to identify potential participants for recruitment (check all that apply): (REQUIRED)

- [x] Review of patients' conditions, history, test results, etc. (includes patients seen in clinic, scheduled for surgery, a procedure, imaging, or tests, or seen in the Emergency Department as well as searching through medical record data for possible cohort identification)
- [ ] Already approved recruitment registry
- [x] Re-contact of participants from the investigators' previous studies
- [x] Referrals from colleagues (attach the 'Dear Colleague' letter or other recruitment materials you will provide to colleagues)
- [x] Referrals from the community / word of mouth
- [x] Advertisements (flyers, brochures, radio or t.v. ads, posting on clinical research sites or social media, presentation of the study at community events/media, etc.)
- [x] Online recruiting tool (describe below)
- [ ] CTSI Recruitment Services unit
- [ ] Posting on UCSF Clinical Trials, ClinicalTrials.gov or other publicly available clinical trial website
- [x] Other method (describe below)

Attach your recruitment materials (e.g., flyers, ads, recruitment letter templates, email text, etc.) in the Other Study Documents section of the Initial Review Submission Packet Form.

* Provide details about the subject identification methods: **(REQUIRED)**

We will mail out an invitation letter, brochure, and response postcard (a self-addressed, stamped envelope will be included for the subject to return the response postcard) to a large numbers of veterans who meet broad criteria, based on diagnostic codes in veteran health records. After patients have had the opportunity to mail in the postcard (after two weeks) or have initiated contact with us themselves, we will provide them with additional study information over the phone and, if interested, we will continue with screening procedures.

We may also request a similar list of veterans from the DoD, in order to reach veterans who may not yet be registered with the VA. The same mailing procedures as described above will be followed for this group of veterans.

All letters, postcards and brochures used in the mailings above will be submitted for IRB approval before mailing.

ResearchMatch

ResearchMatch.org will be utilized as a recruitment tool for this protocol. ResearchMatch.org is a national electronic, web-based recruitment tool that was created through the Clinical & Translational Science Awards Consortium in 2009 and is maintained at Vanderbilt University as an IRB-approved data repository. UCSF is part of the ResearchMatch network, so UCSF researchers are allowed to use this registry with IRB approval.

How it works: Anyone residing in the United States can self-register as a potential research volunteer on ResearchMatch.com. Once registered, volunteers' coded information becomes part of a pool of data that researchers can search through when looking for people to contact about their studies. The researchers then send a recruitment message (attached to this submission) to potentially eligible volunteers through ResearchMatch's secure web system. After receiving the recruitment email from ResearchMatch, the volunteer can click a button to release their contact information to the researcher if they want to learn more about the study. All volunteer information is kept confidential until the volunteer decides to release it.

* Did all the participants of previous studies provide permission to be contacted for future studies: **(REQUIRED)**

(•) Yes ( ) No

11.3 * SEARCHING OF MEDICAL RECORDS: (REQUIRED)

Whose patients are they:

- [x] Investigators' own patients or patients seen within the same practice
- [x] Patients not under the care of the investigators

How and by whom will records be accessed and searched (check all that apply):

- [ ] Self-search in APeX or other medical records source
- [ ] Self-search using UCSF's Research Cohort Selection Tool
- [ ] CTSI Consultation Service Recruitment Services
- [ ] UCSF Academic Research Services (ARS)
- [ ] University of California Research Exchange (UC ReX)
- [x] Other method (describe below)

Describe the other ways medical records may be accessed and searched to identify prospective participants:

We will request from from the VA a list of veterans who meet broad criteria, based on diagnostic codes in veteran health records.

11.4 DETERMINATION OF ELIGIBILITY: How, when, and by whom will eligibility for recruitment be determined:

Eligibility will be determined as follows:

Phone Screen and Phone Verbal Consent
Interested individuals, responding to recruitment efforts, can call the study recruiter for more information. Before study personnel provide information about the study, individuals will be given the option to have their information stored in our recruitment database (CHR Protocol #12-09158) so they may be contacted by program staff regarding other studies they may be eligible for. Study personnel will then provide information about the study. If a potential subject is interested in participating, he or she will be asked a series of eligibility questions. If he/she meets initial requirements for eligibility and is interested in participating, the potential participant will be invited for a remote appointment with the study team, where further eligibility procedures will take place.

Eligibility Procedures
Once informed consent has been obtained, potential subjects will meet remotely with the clinical interviewer and the study doctor. Data from the clinical interview and medical screen will be reviewed in conference with Dr. Neylan & study staff. Eligibility to be enrolled will be determined by the inclusion and exclusion criteria noted above.

11.5 * INITIATION OF CONTACT: Who initiates contact (check all that apply): (REQUIRED)

- [x] Investigators/study team
- [ ] UCSF recruitment unit (e.g. CTSI Consultation Services)
- [x] Potential participant
- [x] Other (explain below)

Provide details about how contact is initiated:

TrialFacts will use an online advertising campaign to recruit participants on behalf of our study. Veterans who see our promotional materials and who are interested in our clinical trial will complete prescreen questionnaires through TrialFacts.org website. All promotional materials and prescreen questionnaires used by TrialFacts will be submitted for IRB approval before TrialFacts recruiting begins.

TrialFacts will send Veterans who are a potential fit for our study (as indicated by their prescreen questionnaire responses) and who choose to complete a phone screen with research support staff a calendar invitation, a confirmation email, as well as email and text message reminders.

Contact may be initiated through ResearchMatch.org. How it works: Anyone residing in the United States can self-register as a potential research volunteer on ResearchMatch.com. Once registered, volunteers' coded information becomes part of a pool of data that researchers can search through when looking for people to contact about their studies. The researchers then send a recruitment message (attached to this submission) to potentially eligible volunteers through ResearchMatch's secure web system. After receiving the recruitment email from ResearchMatch, the volunteer can click a button to release their contact information to the researcher if they want to learn more about the study. All volunteer information is kept confidential until the volunteer decides to release it.

11.6 * HOW IS CONTACT INITIATED: (check all that apply): (REQUIRED)

- [x] In person
- [x] Phone
- [x] Letter / email
- [ ] Website or app
- [x] Other (explain below)

Attach the telephone recruitment script in the Other Study Documents section of the Initial Review Submission Packet Form. If potential participants will initiate contact, attach the telephone screening script that will be used to provide more information about the study and determine if callers are eligible to participate.

Attach the recruitment letter or email template in the Other Study Documents section of the Initial Review Submission Packet Form.

11.7 RECRUITMENT PLAN: Based on the checkboxes you chose above, please provide a narrative describing your recruitment plan. We want to know:

- **Who is conducting the search for potential participants, and how?**
- **How are potential subjects being approached for recruitment? By whom, and when?**

If there will be more than one participant group (e.g. patients, healthy controls, caregivers, family members, providers, etc.), provide details about the recruitment plans for each group. (Recommended length - 100-250 words)

Recruitment Strategies: Recruitment methods and media will include flyers; in-person presentations; website advertisements; internet postings/boards; a study-specific webpage; informational letters; newspaper advertisements; print newsletters; press releases or advertisements in print, internet, television and radio; public service announcements; public notice-board postings; contact with and referral from relevant clinicians; social media, pamphlets; postcards, mailings; informational sessions about the research. In clinical settings, care providers will also be given informational materials to distribute to potential candidates.

The above mentioned recruitment strategies will also take place at social service agencies; community mental health clinics; community organizations /events, including recreational and wellness centers; local professional organizations; residential treatment facilities; consenting support and

recovery centers; local hospitals and healthcare systems; insurance providers; regional employee assistance programs; religious organizations; cultural centers; public transportation vehicles and stations; social clubs; and local universities.

Additionally, we will mail out an invitation letter, brochure, and response postcard (a self-addressed stamped envelope will be included for the subject to return the response postcard) to large numbers of Veterans who meet broad criteria, based on diagnostic codes in Veteran health records. We will contact patients after they have had the opportunity to mail in the postcard (after two weeks). (Before mailing this letter and postcard will be submitted for approval by the IRB.) Staff will contact Veterans via phone call, text message from a VA issued, password protected and encrypted cellular phone. Study staff will also use a VA issued cellular phone to send text messages and VA email to send messages with information about the study to Veterans with whom study staff have established contact. Text messages sent by study staff will be discreet and use the minimum language necessary to communicate information about the study. Email Text messages will include the option to opt-out of further contact and messages will not include personally identifiable information or personal health information.

Interested candidates who respond to recruitment solicitation will initiate contact with the Stress and Health Research Program directly via phone, will be given an overview of the study and its enrollment requirements, and will be initially screened by telephone.

Additionally, recruiters will access a pool of shared participants utilizing a password protected database which is described in depth in approved CHR Protocol #12-09158. Particiants in this database have previously agreed to be contacted about additional research.

If a potential participant is interested and meets initial requirements for participation, he/she will be invited for an appointment with a trained staff member from the Stress and Health Research Program. This remote appointment will entail meeting with trained research personnel who will describe the study in detail, address any questions/concerns and obtain written or electronic informed consent for study participation. After informed consent has been obtained, the initial evaluation interview, and other procedures to determine eligibility will then be conducted.

In addition to the recruitment plans described above, we will be using an online recruitment service provider (TrialFacts.org) to recruit potential participants. To recruit for this study, TrialFacts has created study-specific promotional materials that will be used in their online advertising campaigns. Participants who are interested in our clinical trial will answer a series of prescreen questionnaires to assess whether they are a potential fit. Participants who sign up to participate in the study and pass the online screening questionnaire are then asked to book an appointment for a phone screen with our research staff. These participants will choose a specific time slot to be contacted by our research staff for their phone screening call. TrialFacts will send the participant a calendar invitation, a confirmation email, as well as email and text message reminders. Research staff will contact these interested candidates directly and will provide an overview of the study and its enrollment requirements and will conduct an initial phone screen.

ResearchMatch: We may use ResearchMatch.org to recruit potential participants. We will send out a recruitment message to potentially eligible volunteers. Volunteers who are interested in being contacted will click a button to release their contact information to our research team if they wish to learn more about the study. Our research staff will contact these interested candidates directly and will provide an overview of the study and its enrollment requirements and will conduct an initial phone screen.

11.8 * CONSENT METHODS: How will permission to participate (i.e., informed consent) be obtained from each potential participant. If there will be multiple groups and different plans for consenting each, check all that apply. See the orange Help bubble to the right for more detailed guidance. Participants will (check all that apply): (REQUIRED)

- [x] Sign a paper consent form at the end of the consent discussion (signed consent)
- [x] Sign an electronic consent form using DocuSign (signed consent)
- [ ] Provide online consent through an app, a website, or a survey tool such as Qualtrics or REDCap (waiver of signed consent)
- [x] Be told about the study and be given a handout/information sheet and be asked if they agree to participate (verbal consent - waiver of signed consent)
- [ ] Complete the study activities and turn in materials, as in the case of a completed survey that is placed in a drop box or mailed to the study team (implied consent - waiver of signed consent)
- [ ] Not be able to provide consent and will have a family member consent for them, as in the case of a critically ill or unconscious patient (surrogate consent)
- [ ] Not be able to provide consent (emergency waiver of consent - allowed for minimal risk research or greater than minimal risk research with an approved community consultation plan)
- [ ] Not know about the study, as in the case of chart reviews or observations of public behavior (waiver of consent)
- [x] Other method (describe below)

Attach your consent form, information sheet, or electronic consent text in the Informed Consent Documents section of the Initial Review Submission Packet Form.

11.9 * CONSENT PROCESS: Describe the process for obtaining informed consent, including details such as who will have the consent discussion and when participants will be asked to sign the consent form in relation to finding out about the study: (REQUIRED) We encourage researchers to review our guidance on obtaining and documenting informed consent.

- **If there are multiple groups being consented differently, provide details about the consent process for each group.**
- **If you are relying on verbal or implied consent, provide details about how that will happen.**
- **For studies using online recruitment and consent or consent via mail, provide details here.**

Interested individuals, responding to recruitment efforts, can call the Stress and Health Research Program study recruiter for more information. Study personnel will then provide information about the study over the telephone or via mail.

Before potential participants complete the eligibility phone screen, they will be given the option to have their information stored in our database so they may be contacted by program staff regarding other studies they may be eligible for. Verbal consent will be obtained for the telephone screening interview and for optional storage of screening data for future use. Consent will be documented via the attached Verbal Consent for Screening.

The potential participant will be asked a series of eligibility questions. If eligible, the potential participant will be invited for a remote appointment with a trained staff member from the Stress and Health Research Program.

This appointment will entail meeting with trained research personnel who will describe the study in detail, review the consent form, and address any questions/concerns. The potential participant will be given ample time and opportunity to ask any questions before written or electronic informed consent for study participation is obtained. **Electronic consent will be collected using VA DocuSign.**

* It is important that the people obtaining consent are qualified to do so. Briefly describe the training and experience these individuals have in obtaining informed consent: **(REQUIRED)**

All personnel who obtain informed consent will have at least a Bachelor's degree in Psychology or a related field and will have previous experience in human subjects research and/or work with individials who have PTSD. They will all complete CITI training (for UCSF and VA) and any applicable VA-mandated trainings. Personnel will be familiar with the study protocol, including all procedures, risks and benefits, study timeline and data security and storage. They will be trained on how to assess participants for their understanding of the study and will be prepared to answer the most frequently occurring questions from the previous trial at this site. Staff who are new to consenting for this study will be observed until it is determined they are competent to complete the task.

11.10 * CONSENT COMPREHENSION: Indicate how the study team will assess and enhance the subjects' understanding of study procedures, risks, and benefits prior to signing the consent form (check all that apply): (REQUIRED) Tip: Review the Consent Comprehension - Learning Notes in the Help bubble at the right for specific questions that can be asked to assess comprehension, consider using the UCSF Decision-Making Capacity Assesment Tool, and review our guidance on obtaining written or verbal informed consent for more detail on how to conduct the assessment.

- [x] The study team will engage the potential participant in a dialogue, using open-ended questions about the nature of the study or the experimental treatment, the risks and benefits of participating, and the voluntary nature of participation
- [ ] Potential participants will be asked or shown a series of questions to assess their understanding of the study purpose, procedures, risks and benefits, as well as the voluntary nature of participation (especially appropriate when the consent process happens online or through a mobile health app)
- [ ] Other method (describe below):

Provide details of the other approaches that will be used, if using another method to assess comprehension:

Every possible effort will be made to provide the subject with all required information about the study in a language and format that is easily comprehensible. The consent form is written at an eighth grade reading level with everyday vocabulary and simple sentence structure. A trained research staff member will be present (remotely) to explain the study and obtain consent and thus will be available to address any questions or concerns from the subject. A copy of the consent form will be given to the subject to take home and have for reference. In addition, there is a section in the consent form where we have provided contact information for the subject in case they have questions about the study.

11.11 * DECEPTION: Does this study rely on some deception or misinformation about what the researchers are observing to get valid data? (REQUIRED)

○ Yes ● No

11.13 * WAIVER OF DOCUMENTATION OF SIGNED CONSENT: Select the regulatory category under which the IRB may waive the requirement to obtain *signed* consent for this study:

○ The only record linking the subject and the research would be the consent document and the principal risk would be potential harm resulting from a breach of confidentiality. Each subject will be asked whether they want documentation linking them with the research. 46.117(c)(1)

◉ The research presents no more than minimal risk of harm to subjects and involves no procedures for which written consent is normally required outside of the research context. 46.117(c) (2)

11.14 TIME: What is the estimated time commitment for participants (per visit and in total):

Total spent in pre- and post-intervention assessments (fully remote only): 11 - 13 hours

Total spent in pre- and post-intervention assessments (fully remote with optional in-person visits): 14 -16 hours

Total spent in IE or IMR/PTSD Recovery classes: 36 hours

Screening and Eligibility Assessments:

- Clinician Administered Diagnostic Interview & Questionnaires (approximately 3 hours)
- Medical Screen/Clearance (approximately 30 minutes)
- OPTIONAL Blood draw and urine screen (approximately 30 minutes)

Baseline Assessments:
- Self-Report Questionnaires (approximately 1 hour)
- Neurocognitive Testing (approximately 30 minutes)
- OPTIONAL FMD assessment (approximately 1 hour)

Telehealth Home Visits:
- Study staff may conduct one or more virtual home visits (approximately 30 minutes - 1 hour per visit).

Integrative Exercise Sessions:
- 12 week course; 3 sessions per week; 1 hour per session

Illness Management and Recovery/PTSD Recovery Sessions:
- 12 week course; 3 sessions per week; 1 hour per session

4 and 8-week Self-Report Assessments:
- Self-Report Questionnaires (approximately 30 minutes)

Post 12-week assessments:
- Clinician Administered Diagnostic Interview & Questionnaires (approximately 1-2 hours)
- Self-Report Questionnaires (approximately 1 hour)
- Neurocognitive Testing (approximately 30 minutes)
- OPTIONAL Blood Draw (approximately 30 minutes)
- OPTIONAL FMD assessment (approximately 1 hour)

6-month assessments:
- Clinician Administered Diagnostic Interview & Questionnaires (approximately 1-2 hours)
- Self-Report Questionnaires (approximately 1 hour)
- Neurocognitive Testing (approximately 30 minutes)

IMPORTANT TIP: Ensure this information is consistent with the information provided in the consent form.

11.15 ALTERNATIVES: Is there a standard of care (SOC) or usual care that would be offered to prospective participants at UCSF (or the study site) if they did not participate in this research study:

○ Yes ◉ No

11.16 OFF-STUDY TREATMENT: Is the study drug or treatment available off-study:

- [x] Yes
- [ ] No
- [ ] Not applicable

11.17 OTHER ALTERNATIVES: Describe other alternatives to study participation, if any, that are available to prospective subjects:

Subjects may choose to not take part in this study, or they may choose to take part in another research study.

Participants enrolled in this study will have the option to take part in study #1413833, "Neurobehavioral Correlates of Pain in Post-Traumatic Stress Disorder (PTSD)." In order to coordinate scheduling of study visits and sharing of all data across studies, it will be necessary to share PHI such as name, date of birth, and social security number. The HIPAA authorization for this study will clearly state that by enrolling in this study, the participant is agreeing to sharing of data between the two studies if the participant chooses to take part in study #1413833.

12.0 Waiver of Consent/Authorization for Recruitment Purposes

This section is required when medical records may be reviewed to determine eligibility for recruitment.

12.1 * PRACTICABILITY OF OBTAINING CONSENT PRIOR TO ACCESS: Study personnel need to access protected health information (PHI) during the recruitment process and it is not practicable to obtain informed consent until potential subjects have been identified: (REQUIRED)

- [x] Yes

If **no**, a waiver of consent/authorization is NOT needed.

12.2 * RISK TO PRIVACY: A waiver for screening of health records to identify potential subjects poses no more than minimal risk to privacy for participants:

- [x] Yes

If **no**, a waiver of authorization can NOT be granted.

12.3 * RIGHTS/WELFARE: Screening health records prior to obtaining consent will not adversely affect subjects' rights and welfare:

- [x] Yes

If **no**, a waiver of authorization can NOT be granted.

12.4 * IDENTIFIERS: Check all the identifiers that will be collected prior to obtaining informed consent:

- [x] Names
- [x] Dates
- [x] Postal addresses
- [x] Phone numbers
- [x] Fax numbers
- [x] Email addresses

- [ ] Social Security Numbers*
- [ ] Medical record numbers
- [ ] Health plan numbers
- [ ] Account numbers
- [ ] License or certificate numbers
- [ ] Vehicle ID numbers
- [ ] Device identifiers or serial numbers
- [ ] Web URLs
- [ ] IP address numbers
- [ ] Biometric identifiers
- [ ] Facial photos or other identifiable images
- [ ] Any other unique identifier
- [ ] None

Note: HIPAA rules require that you collect the minimum necessary.

12.5 * HEALTH INFORMATION: Describe any health information that will be collected prior to obtaining informed consent:

Some participants may be recruited via their participation in other research studies within the Stress and Health Research Program, per their consent to be contacted for additional studies or through their inclusion in the Stress and Health recruitment database through the Program-Wide Prescreen (Protocol 12-09158). In these cases, the research data already collected may be reviewed to determine eligibility.

Additionally, the names, service dates, and contact information of some participants may be obtained using broad-based diagnostic codes used in Veteran health records. We will contact patients after they have had an opportunity to mail in an opt-out postcard (after two weeks).

Some study participants who respond to advertisements and outreach will be screened with the telephone screening attached to this application. Verbal consent for screening will be obtained and documented before screening is performed.

Note: HIPAA requires that you collect the minimum necessary.

12.6 * DATA RETENTION/DESTRUCTION PLAN: Describe your plan to destroy any identifiable data collected to determine eligibility for recruitment. This should be done at the earliest opportunity. If you plan to retain identifiable recruitment data, provide the justification for doing so:

The information will be retained, as per current VA and federal policy, until disposition instructions are approved by the National Archives and Records Administration and are published in VHA's Records Control Schedule

13.0 Risks and Benefits

13.1 RESEARCH-RELATED RISKS: Check if your study involves any of these specific research-related risks to participants that may need to be disclosed in the consent form:

- [x] Physical discomforts or pain
- [ ] Risks to employment, or social or legal standing
- [x] Risk that the study team may observe possible evidence of child abuse, elder abuse, or a threat to self or others that they are required to report

For reportable information, include details of the reporting plan below. (See the Help link for Mandated Reporter child and elder abuse resources.)

* For any boxes checked above, describe how you will minimize these risks and discomforts, e.g., adding or increasing the frequency of monitoring, additional screening to identify and exclude people with diminished kidney or liver function, or modification of procedures such as changing imaging studies to avoid giving contrast agent to people who are more likely to suffer side effects from it, etc.: **(REQUIRED)**

Integrative Exercise: As with any program involving new exercises, participants may experience injuries including muscle strain or soreness. We have developed the Integrative Exercise therapy in consultation with experts worldwide who have extensive knowledge with a variety of alternative, integrative medicine exercises to ensure that the program is safe and likely to be efective. In the unlikely event that an emergency injury should arise during a session, our skilled recreational therapists will assess the situation and call 911, if necessary.

Clinical Interviews: Participants are informed via the consent form that we are mandated reporters of abuse and threat to self/others. This is especially relevant during the clinical interview procedure. All interviewers are trained and supervised by a licensed clinical psychologist. Interviewers are trained in assessment of suicide risk; homicide risk; and suspected abuse of children, and of elder and dependent adults.Training also covers mandated reporting requirements and procedures. Interviewers review with all participants mandated reporting and the resultant limits to confidentiality immediately before administering clinical interviews.

13.2 * RISKS: Describe any anticipated risks and discomforts not listed above: (REQUIRED)

Potential Risks and Protection Against Risks:

Clinical Assessment & Questionnaires: The interview and questionnaires may be distressing to some subjects. Subjects will be told that they are free to decline to answer any questions or to stop the interviews at any time. Skilled clinicians who can deal effectively and sensitively with uncomfortable emotional or physical responses to the procedures conduct the interviews and examinations. Subjects can choose to not answer any questions posed to them, and subjects who are experiencing psychiatric distress or a diagnosed disorder will be referred for appropriate treatment.

Neurocognitive Testing: The cognitive testing may be slightly frustrating or produce fatigue and boredom. Subjects can choose to take a break, skip a question, and/or ask the study staff if they have any questions.

Optional Blood Draw: The risks of drawing blood include temporary discomfort from the needle stick, bruising, discomfort, and rarely, infection. Only a qualified nurse or phlebotomist will draw blood following standard procedures. In the event that subjects have an adverse reaction to any of the medical procedures, the lab's central location near the hospital emergency department allows us easy access to emergency response teams.

Optional Brachial artery flow-mediated dilation (FMD assessment): The risks associated with ultrasound measurement of brachial artery flow- mediated dilation include discomfort in the arm during and immediately post occlusion of the brachial artery with the inflatable cuff. To minimize any risks, medical personnel with extensive experience obtaining various types of vascular measurement from patients will be handling all aspects of the procedure. Participants are also free to discontinue the exam if they wish.

Randomization: Subjects have a 50/50 chance of being randomized to the Integrative Exercise vs. IMR/PTSD Recovery Group, which may result in being assigned a treatment that is less

desirable to the individual. Subjects will be clearly informed of this possibility during the consent process. Participants assigned to IMR/PTSD Recovery will be offered free exercise classes after they complete the 12-week assessments.

Integrative Exercise: As with any program involving new exercises, participants may experience injuries including muscle strain or soreness. We have developed the Integrative Exercise therapy in consultation with experts worldwide who have extensive knowledge with a variety of alternative, integrative medicine exercises to ensure that the program is safe and likely to be efective. In the unlikely event that an emergency injury should arise during a session, our skilled exercise instructors will assess the situation and call 911, if necessary. Our exercise instructors will consistently monitor participants via 2-way video-streaming and will be able to call 911 in the case of emergency injury.

During the course of treatment fatigue and/or sleepiness, and/or memory and concentration difficulties may occur (although these symptoms are usually limited to the first 1-2 weeks of treatment). If side effects occur that are not controllable or that are too bothersome, subjects are free to discontinue treatment and withdraw from the study. In the event that a subject experiences persistent side effects, and does not volunteer to withdraw from the study, the PI and /or our DSMB will review his or her case. This review may be followed by the recommendation that the subject be withdrawn from the study.

Illness Management and Recovery/PTSD Recovery Classes: There are no physical risks from participating in the classes. Some of the topics covered in the classes may be emotionally upsetting to participants. Illness Management and Recovery/PTSD Recovery has been developed for use with diverse veteran populations and is shown to be safe. The classes will be conducted by trained mental health professionals. It is very likely that any feelings of upset or distress will be temporary. However, if side effects occur that are not controllable or are too bothersome, participants are free to take a break from class or discontinue treatment and withdraw from the study.

Confidentiality: Participation in research will mean a loss of privacy. The study records will be kept as confidential as is possible under the law. No individual identities will be used in any reports or publications resulting from the study. All subjects will be assigned a unique participant identification number that will only be known to the PI and coordinator. Study information will be coded with this unique number. All information will be kept in secured files. Only study personnel, with the permission of the principal investigator, will have access to the files.

13.3

MINIMIZING RISKS: Describe the steps you have taken to minimize the risks/discomforts to subjects. Examples include:

- **designing the study to make use of procedures involving less risk when appropriate**
- **minimizing study procedures by taking advantage of clinical procedures conducted on the study participants**
- **mitigating risks by planning special monitoring or conducting supportive interventions for the study**
- **having a plan for evaluation and possible referral of subjects who report suicidal ideation**

The following resources are in place to ensure the protection of the rights and welfare of participants:

· Trained and Qualified Personnel – The study team consists of a sufficient number of staff members to effectively carry out the study protocol and ensure that the needs of participants are met. All staff members have been trained in the areas of Human Subjects Protection, Privacy, and Good Clinical Practices. The Principal Investigator, Co-Investigators, and other key study personnel have extensive experience in clinical research and have worked with several CHR protocols.

· Funding – Sufficient funding has been provided to effectively execute the study protocol. Funding is sufficient to allow the Investigators to devote enough time to the project to ensure adequate study design, procedures, and safety monitoring.

· Confidentiality and Privacy – Subjects are free to decline to answer any question or to complete any portion of the interview or, to stop the interviews at any time. Subjects' names are not recorded on test materials; identification numbers are used instead. Subject number identification codes are kept in a secure, locked area. All data are coded and stored in locked file

cabinets in secure locked storage areas. Any electronic data is password protected and stored on secure encrypted server and will have no identifying information.

·   Proximity of Healthcare Resources – Screening procedures, treatment classes, and post-treament assessments will take place remotely. Medical and mental health professionals are available by phone and videoconferencing to address subject concerns if they arise. As well, participants are given contact information for the study doctor and study coordinator and are encouraged to reach out with any questions or concerns.

13.5 * BENEFITS: (REQUIRED) Note: These are the benefits that the IRB will consider during their review. They are not necessarily appropriate to include in the consent form.

Possible immediate and/or direct benefits to participants and society at large (check all that apply):

- [x] Positive health outcome (e.g. improvement of condition, relief of pain, increased mobility, etc.)
- [ ] Closer follow-up than standard care may lead to improved outcomes or patient engagement
- [x] Health and lifestyle changes may occur as a result of participation
- [x] Knowledge may be gained about their health and health conditions
- [x] Feeling of contribution to knowledge in the health or social sciences field
- [ ] The research presents a reasonable opportunity to further the understanding, prevention, or alleviation of a serious problem affecting the health or welfare of children
- [ ] Other benefit (describe below)
- [ ] None

13.6 RISK TO BENEFIT RATIO: Explain why the risks to subjects are reasonable in relation to anticipated benefits, if any, to the participant or society:

All risks are minimal and will be attended to by qualified medical and mental health professionals. All subjects benefit to the extent that every participant (including those originally placed in the IMR/PTSD Recovery group) will have the chance to participate in Integrative Exercise classes. Integrative Exercise may be found helpful in the sample, providing a new treatment option to this population.

13.7 * DATA AND SAFETY MONITORING: Do you have a Data and Safety Monitoring Plan (DSMP) for this study (A DSMP is required for Greater than Minimal Risk research): (Click the Help link for guidance on risk determination) (REQUIRED)

(•) Yes ( ) No

This is not required for minimal risk research but the UCSF IRB strongly recommends one to ensure the data collected are adequate to meet the research aims:

14.0 Data and Safety Monitoring Plan

14.1 * DATA AND SAFETY MONITORING PLAN (DSMP): (REQUIRED) Provide a summary of the DSMP:

All greater than minimal risk studies are required to provide a plan. Lack of an adequate plan is one of the most common reasons why IRB approval is delayed.

Instructions:

Describe the plan for monitoring data quality and participant safety. Key areas that should be included in the plan are:

- An explanation of the plan to monitor data collection, study progress, and safety
- A description of who will perform the monitoring and at what frequency (e.g., the PI only, a contract research organization, a Data and Safety Monitoring Board or Data Monitoring Committee, etc.)
- The type of data and events that will be reviewed (e.g., adverse events, breaches of confidentiality, unanticipated problems involving risk to participants or others, unblinded efficacy data, etc.)
- Procedures and timeline for communicating monitoring results to the UCSF IRB, the study sponsor, and other appropriate entities

As appropriate:

- A plan for conducting and reporting interim analysis
- Clearly defined stopping rules
- Clearly defined rules for withdrawing participants from study interventions

This study will use a Data Safety Monitoring Board, which will meet quarterly and will be available to have additional ad hoc meetings to review any serious adverse events. This protocol and all associated consent forms and questionnaires will be approved and monitored by our University of California, San Francisco Committee on Humans Subjects Research (CHR). As such, all adverse events that are reported to study staff during classes or study visits will be reported to the UCSF CHR. Per the CHR policy, serious adverse events must be reported immediately and all others are reported annually.
In the event that a subject experiences a serious adverse event, and does not volunteer to withdraw from the study, their case will be reviewed by the PI and/or our DSMB. This review may be followed by the recommendation that the subject be withdrawn from the study.

Other provisions for subject safety:

- During the trial, subjects are seen three times a week. All study staff are trained to report adverse events or any concerns about a subject's wellbeing to the Study Coordinator and Principal Investigator. Thus, any untoward event that might occur will be detected almost immediately.
- To ensure that patients have access to more immediate attention, they are provided with a cell phone number for the Study Coordinator, which will give them nearly immediate access to assistance over the course of the study.

14.2 * DATA AND SAFETY MONITORING BOARD (DSMB): (REQUIRED) Will a Data and Safety Monitoring Board (DSMB) be established:

(•) Yes
( ) No

14.3 DSMB DETAILS: Provide details about the DSMB, including meeting frequency, and the affiliations and qualifications of members: Attach the DSMB charter to the Other Study Documents section. If the DSMB has not yet been established, submit details and the charter to us as soon as they become available.

The DSMB has been formed and consists of three medical UCSF/SFVAMC medical professionals: Drs. William Wolfe, Ellen Herbst, and Brian Mohlenhoff. Dr. William Wolfe is an Associate Professor of Psychiatry at UCSF and the Medical Director of the PTSD program at the SFVAMC. Dr. Ellen Herbst is an Associate Professor of Psychiatry at UCSF and a Psychiatrist at the SFVAMC. Dr. Brian Mohlenhoff is an Assistant Professor of Psychiatry at UCSF and the Director of Pharmacotherapy for the PSTD program at the SFVAMC.

The DSMB meets quarterly and is available to have additional ad hoc meetings to review any serious adverse events.

15.0 Confidentiality, Privacy, and Data Security

15.1 PROTECTING PRIVACY: Indicate how subject privacy will be protected:

- [x] Conduct conversations about the research in a private room
- [x] Ask the subject how they wish to be communicated with – what phone numbers can be called, can messages be left, can they receive mail about the study at home, etc.
- [ ] Take special measures to ensure that data collected about sensitive issues do not get added to their medical records or shared with others without the subject's permission
- [ ] Other methods (describe below)

15.2 SENSITIVE DATA: Do any of the instruments ask about illegal or stigmatized behavior:

(●) Yes ( ) No

IMPORTANT NOTE: Indicate in the consent form what kinds of sensitive information will be collected.

15.3 SIGNIFICANT CONSEQUENCES OF A LOSS OF PRIVACY OR CONFIDENTIALITY: Could a breach of privacy or confidentiality result in any significant consequences to participants, such as criminal or civil liability, loss of state or federal benefits, or be damaging to the participant's financial standing, employability, or reputation:

( ) Yes (●) No

15.4 EXTRA CONFIDENTIALITY MEASURES: Explain any extra steps that will be taken to assure confidentiality and protect identifiable information from improper use and disclosure, if any:

The study records will be kept as confidential as is possible under the law. Although VA hospital personnel will not have access to study materials, a medical record will be created because of participation in this study. The following information will be included in the medical record: documentation that the subject has been medically cleared to exercise, the results of the exercise treadmill test, and the results of the standard lab tests. Therefore, other doctors may become aware of the individual's study participation. Hospital regulations require that all health care providers treat information in medical records confidentially. At the time of consent, subjects will be asked to sign forms to authorize the release of their personal health information for research purposes. Participants in the PTSD Recovery Classes will have each class scheduled as an appointment in their VA medical record (CPRS). A progress note for each class attended will be added to the medical record. These are the same procedures that would be followed if the veteran was enrolled in any group or class as a VA patient not in a research study.

If information from this study is published or presented at scientific meetings, the participant's name and other personal information will not be used.

All study information, such as the interviews and questionnaires, will be coded with a code number unique to the study. Only study personnel, with the permission of the principal investigator, will have access to the key with the name and ID codes.

Audio recordings – Clinical Interview: The audio recordings may make a participant somewhat more uncomfortable than he or she would be without the taping. The recordings will be labeled with a unique code number and will be used only by research personnel in order to calibrate the clinicians' ratings on the standardized interview format. The audio recordings will be retained in a secure location (the digital recordings will be encrypted and passcode protected and stored and accessed via the secure VA server). Subjects will be informed that their screening clinical interviews will be audio recorded for the purpose of allowing the research team to ensure

consistency across all clinical interviews. They will be informed that the recordings will be maintained under secure conditions at all times and identified only by the unique Subject ID number.

Organizations that may look at and/or copy subjects' medical records for research, quality assurance, and data analysis include the UCSF IRB (a group of people who review research to protect participants' rights) and the Department of Veterans Affairs Regulatory Personnel (the group which protects research participants' rights at the VA).

Additionally, recruiters will access a pool of shared participants utilizing a password-protected database at the SFVA. Individuals in this database have been asked brief screening questions from a Program Wide Prescreen, which is described in depth in approved Protocol 12-09158, or from a specific study. Recruiters will contact individuals from this database who can decide if they want to answer the study specific screen. Informed consent will be obtained by research staff prior to beginning any procedures.

To assure confidentiality and protect identifiers from improper use and disclosure, if any, screening data will be stored in firewall and password-protected computers within the SFVAMC, and only accessed by program staff. It is not practical to code data at the recruitment stage, as personal identifiers are required to contact participants for participation in related CHR-approved studies.

15.5 * REPORTABILITY: Do you anticipate that this study may collect information that State or Federal law requires to be reported to other officials, such as elder abuse, child abuse, or threat to self or others: (REQUIRED)

(•) Yes ( ) No

The confidentiality and privacy section of the consent form should include this as a possible risk of participation.

* Describe the types of reportable information the research team may encounter and provide the details of the reporting plan: **(REQUIRED)**

Exceptions to the protection of a participant's confidentiality would occur if it were learned through the initial interview that the participant was a danger to him/herself or to others, that a child had been abused or neglected, or that an elder or dependent had been abused. Should this happen, the appropriate authorities would be notified, as required by law.

15.6 CERTIFICATE OF CONFIDENTIALITY: Will this study obtain a Certificate of Confidentiality:

( ) Yes (•) No

15.7 SHARING OF RESEARCH RESULTS: Will there be any sharing of EXPERIMENTAL research test results with subjects or their care providers:

(•) Yes ( ) No

Note: This is unusual and not recommended, particularly in cases where the tests are carried out in a non-CLIA certified laboratory, the results are of unproven clinical significance, or where there are not known preventative strategies and/or treatments. If these are the most likely scenarios for your study, you should check 'No.'

If you have an incidental finding of clear clinical significance, call the HRPP QIU at 415-476-1814 for a consult.

Explain under what circumstances research results may be shared:

Results of the blood will not be shared with study participants.The only exception would be if the results were abnormal and required clinical follow-up; we will then contact the participant and/or primary care physician. This information will be communicated to the medical professional only with the permission of the participant, indicated by signing the informed consent and HIPAA release.

Any abnormal results will be communicated to the subject's own physician, regardless of their veteran status. It will then be the responsibility of the subject's physician to interpret the results and follow-up as needed.

Participants enrolled in this study will have the option to take part in study #1413833, "Neurobehavioral Correlates of Pain in Post-Traumatic Stress Disorder (PTSD)." In order to coordinate scheduling of study visits and sharing of all data across studies, it will be necessary to share PHI such as name, date of birth, and social security number. The HIPAA authorization for this study will clearly state that by enrolling in this study, the participant is agreeing to sharing of all study data between the two studies if the participant chooses to take part in study #1413833.

15.9 * HIPAA APPLICABILITY: Study data will be: (REQUIRED)

- [x] Derived from a medical record (e.g. APeX, OnCore, etc. Identify source below)
- [x] Added to the hospital or clinical medical record
- [ ] Created or collected as part of health care
- [ ] Used to make health care decisions
- [x] Obtained from the subject, including interviews, questionnaires
- [ ] Obtained ONLY from a foreign country or countries
- [ ] Obtained ONLY from records open to the public
- [x] Obtained from existing research records
- [ ] None of the above
- [ ] Derived from the Integrated Data Repository (IDR) or The Health Record Data Service (THREDS) at SFGH

In addition to signing a consent form, each subject will have to sign the UCSF Research Subject Authorization Form (HIPAA Form). Upload the HIPAA Authorization Form in the Other Study Documents section of the Initial Review Submission Packet Form. Failure to have patients sign the HIPAA Authorization is one of the most common findings from QIU Routine Site Visits. Please call the IRB office at 415-476-1814 if you have questions about HIPAA research requirements.

If derived from a medical record, identify source:

CPRS

15.10 * IDENTIFIERS: Check all identifiers that will be collected and included in the research records, even temporarily: (REQUIRED)

- [x] Names
- [x] Dates
- [x] Postal addresses (if only requesting/receiving zip codes check Yes to the Zip Code question below instead of checking this box)
- [x] Phone numbers
- [x] Fax numbers
- [x] Email addresses
- [x] Social Security Numbers*

- [x] Medical record numbers
- [ ] Health plan numbers
- [ ] Account numbers
- [ ] License or certificate numbers
- [ ] Vehicle ID numbers
- [ ] Device identifiers or serial numbers
- [ ] Web URLs
- [ ] IP address numbers
- [ ] Biometric identifiers
- [ ] Facial photos or other identifiable images
- [ ] Any other unique identifier
- [ ] None

*** Required for studies conducted at the VAMC**

* Could study records include *ANY* photos or images (even 'unidentifiable' ones): **(REQUIRED)**

○ Yes ◉ No

* Please provide a justification for including the Social Security Number (SSN) in your data set. Best practices dictate that you store the SSN separately from the full data set in a password protected file. **(REQUIRED)**

SSN is required for processing study payment. It will be stored with the study consent and other documents containing PHI. It will not be stored with any coded data.

15.12 * PATIENT RECORDS: Will health information or other clinical data be accessed from UCSF Health, Benioff Children's Hospital Oakland, or Zuckerberg San Francisco General (ZSFG): (REQUIRED)

○ Yes ◉ No

15.15 * HIPAA - PERMISSION TO ACCESS SENSITIVE DATA: Does the research require access to any of the following types of health information from the medical record: (check all that apply) (REQUIRED)

- [ ] Drug or alcohol abuse, diagnosis or treatment
- [ ] HIV/AIDS testing information
- [ ] Genetic testing information
- [x] Mental health diagnosis or treatment
- [ ] None of the above

Important note: Ensure that participants initial the corresponding line(s) in Section C of the HIPAA authorization form during the consent process.

15.19 * DATA COLLECTION AND STORAGE: (check all that apply): (REQUIRED)

Collection methods:

- [ ] Electronic case report form systems (eCRFs), such as OnCore or sponsor-provided clinical trial management portal
- [ ] UCSF ITS approved Web-based online survey tools: Qualtrics or RedCap
- [x] Other web-based online surveys or computer-assisted interview tool
- [ ] Mobile applications (mobile or tablet-based)
- [ ] Text Messaging

- [ ] Wearable devices
- [x] Audio/video recordings
- [ ] Photographs
- [x] Paper-based (surveys, logs, diaries, etc.)
- [ ] Other:

* What online survey or computer assisted interview tool will you use: **(REQUIRED)**

- [x] Qualtrics (Recommended)
- [ ] RedCAP (Recommended)
- [ ] Survey Monkey (NOT recommended and may require UCSF ITS Security review)
- [x] Other

* What's the name of the survey tool and who is it owned by: **(REQUIRED)**

Millisecond Inquisit Web for remote neurocognitive testing

If the survey tool is not provided by the study sponsor, and the survey tool stores data on a server, vendor, cloud, or 3rd party, contact datasecurity@ucsf.edu to determine if you need to complete a security assessment.

* Data will be collected/stored in systems owned by (check all that apply): **(REQUIRED)**

- [ ] Study sponsor
- [x] UCSF data center (including OnCore, RedCap, Qualtrics, and MyResearch)
- [ ] UCSF encrypted server, workstation, or laptop residing outside of UCSF data center
- [ ] Personal devices, such as laptops or tablets that are not owned or managed by UCSF
- [x] SF VAMC
- [ ] Zuckerberg San Francisco General Hospital
- [ ] Benioff Children's Hospital Oakland
- [ ] Langley Porter Psychiatric Institution
- [ ] Other UCSF affiliate clinic or location (specify below)
- [ ] Cloud vendor such as Amazon Web Services (AWS), Salesforce, etc. (specify below)
- [ ] Other academic institution
- [ ] 3rd party vendor (business entity)
- [x] Other (explain below)

Please consult with the VA's Clinical Research Office at 415-221-4810 x 2-6425 about the VA's requirements for data storage and security.

* Provide more details about where study data will be stored: **(REQUIRED)**

Neurocognitive data collected using Millisecond Inquisit Web will upload to our Millisecond account, where we can login to access and download data. Millisecond Inquisit will save data in a highly reliable redundant store with copies maintained on multiple servers in different facilities. Please see below for Millisecond's Security Statement, found here: **https://www.millisecond.com/products/securitystatement.aspx**

"Millisecond is dedicated to protecting all customer data using industry best standards. This Security Statement is intended to provide a transparent look at our security infrastructure and practices to help assure that your data are sufficiently protected.

Millisecond’s highest priority is the protection and reliability of customer data. Our servers are protected by high-end firewall systems, and scans are performed regularly to ensure that any

vulnerabilities are quickly found and patched. All services have quick failover points and redundant hardware, with complete backups performed nightly. Data are stored redundantly across data centers for resiliency and availability during disasters.

Millisecond provides each customer a unique username and enforces strong passwords that must be entered each time a customer logs on. The user remains authenticated only for the duration of the session and is automatically logged off after 30 minutes of inactivity. This system ensures that customer data can only be accessed by authenticated and authorized users. Millisecond uses AWS Cognito to manage authentication and does not store user passwords locally. For additional account security, Millisecond customers can enable multi-factor authentication with a one-time password sent via SMS.

Customer data are processed and stored in world-class data center facilities in Oregon, USA and Ireland, EU. Data are not moved around to other locations. The data centers are housed in nondescript facilities. Physical access is strictly controlled both at the perimeter and at building ingress points by professional security staff utilizing video surveillance, intrusion detection systems, and other electronic means. Authorized staff must pass two-factor authentication a minimum of two times to access data center floors. All visitors and contractors are required to present identification and are signed in and continually escorted by authorized staff.

Facilities are equipped with fire detection and suppression equipment, multiple backup power systems, and climate and temperature control. Servers are decommissioned and disposed using processes that prevent unauthorized access to data.

The servers reside behind high-availability firewalls and are monitored using state of the art systems for detection and prevention of various threats including denial of service, man in the middle, IP spoofing, port scanning, and packet sniffing. Automated network security audits using the industry standard SSAE-16 method are conducted to the standards and requirements of the SANS/FBI security test, the U.S. Department of Homeland Security's published recommendations and the Payment Card Industry Data Security Standard.

Millisecond encrypts all data in transit by enforcing the latest versions of Transport Layer Security (TLS) encryption (also known as HTTPS). Millisecond encrypts all data at rest using the industry standard AES-256 cypher.

Millisecond deploys the general requirements set forth by many Federal Acts, including the FISMA Act of 2002. We meet or exceed the minimum requirements as outlined in FIPS Publication 200. We also comply with FERPA for protecting student privacy.

Since our subscribers control their users and their data, it is important for the users to practice sound security practices by using strong account passwords and restricting access to their accounts to authorized persons. For added protection, Millisecond supports multi-factor authentication for logging into user accounts.

Regarding HIPAA, HITECH, and specific data types: Millisecond provides general research software and other services where all data are processed equally, without regard to how a customer might classify their data. As such, Millisecond cannot declare or represent any data entered into its services. Any processing of specific data types are purely incidental, and not required to use the services.

HITECH (Health Information Technology for Economic and Clinical Health Act) updated HIPAA rules to ensure that data are properly protected and best security practices followed. Millisecond safeguards all customer data, and uses secure data centers to ensure the highest protection as per HITECH requirements."

15.21 * DATA SHARING: During the lifecycle of data collection, transmission, and storage, will identifiable information be shared with or be accessible to anyone outside of UCSF: (REQUIRED)

(•) Yes ( ) No

* Who will have access to the data: **(REQUIRED)**

- [ ] Collaborators listed in the study application
- [ ] NIH or other shared data repository
- [ ] Sponsors
- [ ]

FDA

- [x] Other 3rd party (such as vendors/contractors)

IMPORTANT: The IRB now recommends that all consent forms include a provision for sharing of de-identified/coded data to permit re-use of data for secondary research purposes. This doesn't apply if you've been granted a waiver of consent for this study.

* Provide the details of whom the data will be shared with and what types of information and identifiers will be shared: **(REQUIRED)**

The YMCA will receive the names and contact information of all individuals who receive a 6-month YMCA membership as a result of their study completion. No other study data or individual health information will be shared with the YMCA. Participants will consent to this release of their name via the consent form and HIPAA authorization.

Participants enrolled in this study will have the option to take part in study #1413833, "Neurobehavioral Correlates of Pain in Post-Traumatic Stress Disorder (PTSD)." In order to coordinate scheduling of study visits and sharing of all data across studies, it will be necessary to share PHI such as name, date of birth, and social security number. The HIPAA authorization for this study will clearly state that by enrolling in this study, the participant is agreeing to sharing of all study data between the two studies if the participant chooses to take part in study #1413833.

15.22 * DATA SHARING METHODS: How will data be securely shared with the 3rd party: (REQUIRED)

- [ ] Collaborators will access data in MyResearch
- [ ] Collaborators will access data in REDCap
- [ ] Collaborators will be sponsored as an affiliate and be treated as an UCSF user (includes using UCSF Box)
- [ ] UCSF Secure Email will be used to share data
- [ ] Collaborator’s or Sponsor’s system will be used (specify below)
- [x] Other method (describe below)

Please provide details about how the data will be shared:

The YMCA will receive the names and contact information of all individuals who receive a 6-month YMCA membership as a result of their study completion. No other study data or individual health information will be shared with the YMCA. Participants will consent to this release of their name via the consent form and HIPAA authorization.

16.0 Financial Considerations

16.1 * PAYMENT: Will subjects be paid for participation, reimbursed for time or expenses, or receive any other kind of compensation: (REQUIRED)

(•) Yes ( ) No

16.2 PAYMENT METHODS: Subjects payment or compensation method (check all that apply):

Payments will be (check all that apply):

- [x] Cash
- [x] Check
- [ ] Gift card
- [ ] Debit card
- [ ] UCSF Research Subject Payment Card
- [ ] Reimbursement for parking and other expenses

☑ Other:

Specify **other** payment/compensation method:

Direct deposit.

16.3 PAYMENT SCHEDULE: Describe the schedule and amounts of payments, including the total subjects can receive for completing the study:

- **If there are multiple visits over time, explain how payments will be prorated for partial completion**
- **If deviating from recommendations in Subject Payment Guidelines, include specific justification below**

In return for their time and effort, subjects will be compensated for taking part in this study according to the schedules described below:

Total possible compensation (in addition to receiving free IMR/PTSD Recovery Classes and/or Integrative Exercise):

Fully Remote Only: Up to $310, plus 6-month YMCA membership

Fully Remote with Optional In-Person Assessments: Up to $400, plus free breakfast post-FMD procedures, and 6-month YMCA membership

Screening/Eligibility Assessments:

- Clinician Administered Diagnostic Interview & Questionnaires = $55

Pre-treatment Assessments:

- Medical Screen/Clearance = $15
- Self-Report Questionnaires & Neurocognitive testing = $25

4 week Self-Report Questionnaires = $25

8 week Self-Report Questionnaires = $25

Post 12-week Assessments:

- Clinician Administered Diagnostic Interview & Questionnaires = $25
- Self-Report Questionnaires & Neurocognitive Testing = $25

** A 6-month free YMCA gym membership will be provided after the trial (at the completion of all Post 12-week Assessments) as an additional completion bonus for completing all assessments, questionnaires, and study procedures, and to promote exercise continuity/commitment to healthy living. If there is no YMCA available within 25 miles of participants homes, we will either send them additional payment, equivalent to the cost of a YMCA membership, so that they may choose a fitness facility nearby or use the funds to purchase the participant additional at-home exercise equipment or online fitness classes. Because of limitations imposed by the COVID-19 pandemic (e.g., YMCA closures), we may provide direct deposit payments for the amount of a 6-month YMCA membership to eligible Veterans.

6 Month Follow-Up Assessments:

- Clinician Administered Diagnostic Interview & Questionnaires/Neurocognitive Tests = $55
- Completion Bonus = $60

Optional in-person pre- and post-treatment assessments:

- Pre-treatment blood draw = $15
- Pre-treatment FMD = $30 (in addition to free breakfast on the day of your appointment)
- Post-treatment blood draw = $15
- Post-treatment FMD = $30 (in addition to free breakfast on the day of your appointment

16.4 COSTS TO SUBJECTS: Will subjects or their insurance be charged for any study activities:

○ Yes ◉ No

17.0 Other Approvals and Registrations

17.4 OTHER APPROVALS: Indicate if this study involves other regulated materials and requires approval and/or authorization from the following regulatory committees:

☑ Institutional Biological Safety Committee (IBC)

Specify BUA #:

NEYT-16-1

☐ Institutional Animal Care and Use Committee (IACUC)

Specify IACUC #:

☐ Controlled Substances

18.0 Qualifications of Key Study Personnel and Affiliated Personnel

NEW: January 2019 - Affiliated personnel who do not need access to iRIS no longer need to get a UCSF ID. Instead, add them below in the Affiliated Personnel table below.

18.1 Qualifications of Key Study Personnel:

Instructions:

For UCSF Key Study Personnel (KSP)* listed in **Section 3.0,** select the KSP from the drop down list and add a description of their study responsibilities, qualifications and training. In study responsibilities, identify every individual who will be involved in the consent process. Under qualifications, please include:

- Academic Title
- Institutional Affiliation (UCSF, SFGH, VAMC, etc.)
- Department
- Certifications

NOTE: This information is required and your application will be considered incomplete without it. If this study involves invasive or risky procedures, or procedures requiring special training or certification, please identify who will be conducting these procedures and provide details about their qualifications and training. Click the orange question mark for more information and examples.

Training Requirements:

The IRB requires that all Key Study Personnel complete Human Subjects Protection Training through **CITI** prior to approval of a new study, or a modification in which KSP are being added. More information on the CITI training requirement can be found on our **website.**

* **Definition of Key Study Personnel and CITI Training Requirements (Nov, 2015):** UCSF Key Study Personnel include the Principal Investigator, other investigators and research personnel who are directly involved in conducting research with study participants or who are directly involved in using study participants' identifiable private information during the course of the research. Key Personnel also include faculty mentors /advisors who provide direct oversight to Postdoctoral Fellows, Residents and Clinical Fellows serving as PI on the IRB application.

KSP Name	Description of Study Responsibilities - Briefly describe what will each person be doing on the study. If there are procedures requiring special expertise or certification, identify who will be carrying these out. Also identify who will be obtaining informed consent.	Qualifications, Licensure, and Training
Dr. Neylan, Thomas MD	Dr. Neylan will be the Principal Investigator for this study. He will assume overall scientific and administrative leadership for the study. He will be responsible for supervising the study team with regards to the recruitment,diagnostic assessment, and enrollment of subjects and the coordination of all study procedures.<br><br>Dr. Neylan will have overall responsibility for the standardization of data collection, data quality control, data analysis, and interpretation. He will have overall responsibility for subject safety, rights, and welfare. He will be an active participant in the preparation of abstracts and manuscripts and will assure the dissemination of study findings in the professional and scientific communities.	Thomas Neylan, M.D. has a joint appointment at UCSF and the SFVAMC. He is a Professor of Psychiatry at UCSF and Director of the Stress and Health Research Program.<br><br>Dr. Neylan has been active in the field of sleep and PTSD research for the past 15 years. During that time, he has been the Principal Investigator or Co-Principal Investigator for CHR protocols in psychopharmacology, psychobiology, and treatment of PTSD.
Chesney, Margaret A, PhD	As Co-Investigator, Dr. Chesney will provide senior level guidance, particularly regarding aspects of the project	Dr. Chesney is Professor of Medicine and former Director of the Osher Center for Integrative Medicine. She was the interim director of the National Institute for

	related to integrative medicine.	Complementary and Integrative Medicine at the NIH and involved in its research.
Cohen, Beth, MD, MA	Co-Investigator	Dr. Cohen is an Assistant Professor in the Department of Medicine at UCSF and physician at the SF VA Medical Center
Hlavin, Jennifer	Ms. Hlavin is the Lab Manager of the Stress and Health Research Program. She will be supervising the coordination of the study.	Jennifer Hlavin, MS in Counseling, has 10 years of clinical research experience at the San Francisco VA Medical Center. She has been a Study Coordinator and Lab Manager for the past 8 years, working with diverse groups of Veteran and non-Veteran research participants.
Dr. Mehling, Wolf MD, MD	As Co-Investigator, Dr. Mehling will work with Dr. Neylan in the development and implementation of the study, particularly regarding aspects of the study related to integrative medicine.	Dr. Mehling is an Assoc. Prof. of Clinical Family and Community Medicine and on faculty at the Osher Center for Integrative Medicine. His research focuses on body-oriented complementary therapies and mind-body interactions.
Metzler, Thomas J	Thomas Metzler, M.S., will be the Biostatistician for the study. He will be responsible for the overall quality and fidelity of the study data collected. After the conclusion of the study, he will conduct the statistical analyses on all data gathered and he will have an extensive role in the preparation of manuscripts, slides, and poster presentations of the data derived in the study.	Mr. Metzler has served as the primary biostatistician for the PTSD Research Program at the SFVAMC for the past 12 years and has conducted and analyzed psychophysiological assessments on multiple funded studies.
Mayzel, Olga	Data Manager	Olga Mayzel has extensive experience performing data management at VA, database design, and application development for several large-scale studies. Ms. Mayzel will be responsible for database development and management.

Goldstein, Lizabeth	Research Fellow	Lizabeth Goldstein is a MIRECC Fellow at the SFVAMC and will be involved in data analysis and dissemination of results.
Boyd, Jennifer E	As Co-Investigator, Dr. Boyd will oversee the Illness Management and Recovery Classes. She will supervise the Peer Specialist, a Recreation Therapist and psychology doctoral students who will co-facilitate the IMR classes.	Dr. Boyd is the Associate Chief for Psychosocial Recovery Services, and oversees the Psychosocial Rehabilitation and Recovery Center (PRRC), the Recreation Therapy program, and the Peer Specialist program. The PRRC will provide research participants and will host the IMR classes.
Shumaker, Erik	Will consult with the Investigator and the Research Psychologist on clinical assessment of PTSD.	Dr. Shumaker is a Clinical Psychologist at the San Francisco VA Medical Center.
Bertenthal, Daniel S	Will serve as consulting data manager and biostatistician.	Dan Bertenthal received his B.A. in Sociology of Culture and B.S. in Microbiology from the University of California, San Diego. He continued at the University of California, Berkeley School of Public Health, where he developed quantitative skills while completing an MPH in Epidemiology & Biostatistics. He has worked with health services researchers at the San Francisco VA Medical Center since 2001.
Cheng, David	Technician, Flow Mediated Dilation Measures	David Cheng currently works in the CRC located at the San Francisco VA. He has extensive experience obtaining various types of vascular measurements from patients.
		Victor Antonetti will serve as a group exercise instructor for this study. Victor has over 17 years experience as a personal trainer/group fitness

Antonetti, Victor	Senior Exercise Instructor	instructor. In addition, he is a NASM Certified Personal Trainer, a NASM Corrective Exercise Specialist, and a Certified Yoga instructor.
Williams, Chanda	Exercise Instructor	Chanda Williams will serve as a group exercise instructor for this study.
Gasper, Warren J	Other Investigator	Dr. Warren Gasper is Assistant Professor of Clinical Surgery, Division of Vascular and Endovascular Surgery at UCSF. He is Acting Chief of Vascular Surgery at the San Francisco VA Medical Center, and is the Director of the Vascular Integrated Physiology and Experimental Therapeutics (ViperX) Lab at the SFVAMC.
Phan, Jordan Dominique	Study Recruiter	
Richards, Anne	Other Investigator	Dr. Richards is Associate Clinical Professor at the University of California, San Francisco and Staff Psychiatrist at the San Francisco VA Medical Center. Expertise in the treatment of PTSD and sleep disorders gained through years of experience treating male and female veterans with PTSD with medication and psychotherapy. In 2015, transitioned from a predominantly clinical role at the SFVAMC/UCSF to a VA research Career Development Award focused on understanding how sleep disturbance contributes to the consolidation and maintenance of PTSD in male and female veterans.
Garcia Guerra, Sergio R	Study Coordinator	VA/UCSF personnel with experience conducting clinical studies of PTSD /TBI.
		UCSF/VA personnel.

Dr. Strigo, Irina PhD, PhD	Other Investigator	Research Physiologist at the SFVAMC and Associate Professor of Psychiatry at UCSF, is an expert in conduction human pain research in veteran and non-veteran populations.
Palyo, Sarah A	Other Investigator	VA personnel. Clinical Director of the Intensive Pain Rehabilitation Program.
Muratore, Laura	Study Coordinator	Laura Ann Muratore holds a bachelors degree in Psychology and a minor in Applied Psychology from UC Santa Barbara and is on track to complete her Master’s degree in Research and Experimental Psychology from San Jose State University (expected December 2019). She has four years of experience in human subjects research and has completed CITI trainings for human subjects research.
West, Anna	Dr. West will be responsible for the oversight of the diagnostic psychological and neurocognitive assessments that will be administered to subjects throughout the study. She will provide clinical supervision for the mental health clinicians who will be administering the diagnostic psychological and neuropsychological assessments.	Anna West, Ph.D. graduated from California School of Professional Psychology and has been a licensed Marriage and Family Therapist since 2004. Prior to starting her PhD, Anna successfully operated her own private practice. She completed her pre-doctoral residency at APA accredited California Pacific Medical Center, working with an inpatient population. Her post-doctoral residency was at APA accredited Kaiser Permanente, San Rafael. Anna’s clinical interests include providing evidenced-based treatments for psychosis, serious mental illness, and trauma.
		Emily Stenson has a BA in psychology from CSU Chico, and is currently enrolled in a masters program in research and

Stenson, Emily P	Study Coordinator	experimental psychology at San Jose State University. She is a current study coordinator at NCIRE.
Dr. O'Donovan, Aoife PhD, PhD	Other Investigator	Dr. Aoife O'Donovan is an Associate Professor in the department of Psychiatry & Weill Institute for Neurosciences and THRIVE Lab Director.

18.2 Affiliated Personnel:

Instructions:

This section is for personnel who are not listed in **Section 3.0: Grant Key Personnel Access to the Study** because their names were not found in the User Directory when both the iRIS Database and MyAccess directories were searched. Add any study personnel who fit ALL of the following criteria in the table below:

- They meet the definition of Key Study Personnel (see above), **and**
- They are associated with a UCSF-affiliated institution (e.g., VAMC, Gladstone, Institute on Aging, Vitalant, NCIRE, SFDPH, or ZSFG), **and**
- They do not have a UCSF ID, **and**
- They do not need access to the study application and other study materials in iRIS.

Note: Attach a **CITI Certificate** for all persons listed below in the **Other Study Documents** section of the **Initial Review Submission Packet Form** after completing the **Study Application**.

Click the orange question mark icon to the right for more information on who to include and who not to include in this section.

Do not list personnel from outside sites/non-UCSF-affiliated institutions. Contacts for those sites (i.e. other institution, community-based site, foreign country, or Sovereign Native American nation) should be listed in the **Outside Sites** section of the application.

If there are no personnel on your study that meet the above criteria, leave this section blank.

Name	Institution	Telephone	E-mail	Role
Induni Wickramasinghe	VA	415-221-4810 x26624	iwickramasinghe@paloaltou.edu	Project Staff
Adriana Costa	San Francisco VA Medical Center	415-221-4810 x26624	acosta@wi.edu	Project Staff
Nomi Kosman-Wiener	San Francisco VA Medical Center	415-221-4810 x26624	nkosman-wiener@wi.edu	Project Staff

Erika Roach	San Francisco VA Medical Center	415-221-4810 x26624	erika.ro@berkeley.edu	Project Staff
Elizabeth Roskey	San Francisco VA Medical Center	415-221-4810 x26624	eroskey@wi.edu	Project Staff
Lexie Thomas	San Francisco VA Medical Center	415-221-4810 x26624	lthomas@wi.edu	Project Staff
Parker Kelley	University of California San Francisco	415 221 4810 x24959	parker.kelley@ucsf.edu	Clinical Research Associate

Please describe the study responsibilities and qualifications of each affiliated person listed above:

Dr. Parker Kelley is a data science postdoctoral fellow in PTSD in the Department of Psychiatry & Weill Institute for Neurosciences (UC San Francisco). Dr. Kelley will be assisting with data analysis.

Adriana Costa will be administering the diagnostic psychological assessments. She is a fifth-year graduate student working towards her Doctorate in Clinical Psychology at the Wright Institute. Adriana has trained as a school-based therapist and community mental health counselor in the Bay Area. She has also supported incarcerated youth and their families in San Mateo County.

Email: **acosta@wi.edu**

Naomi Kosman-Wiener will be administering the diagnostic psychological assessments. She is a third-year graduate student working towards her Doctorate in Clinical Psychology at the Wright Institute. She has worked as a psychology trainee at school settings and community mental health clinics in San Francisco and the East Bay and is currently working at a community college in the East Bay. Prior to graduate school, Naomi worked as a counselor at a residential program in San Francisco for teenagers.

Email: **nkosman-wiener@wi.edu**

Erika Roach will be administering the diagnostic psychological assessments. She is a second-year doctoral student in Clinical Science at UC Berkeley and a trainee in the UC Berkeley Psychology Clinic. Her research interests include stress, emotion regulation, and developmental psychopathology. She received her B.A. in Psychology and Human Biology and her M.A. in Psychology from Stanford University.

Email: **erika.ro@berkeley.edu**

Elizabeth Roskey will be administering the diagnostic psychological assessments. She is a third-year graduate student working towards her Doctorate in Clinical Psychology at the Wright Institute. She was worked as a therapist at several community mental health clinics in the Bay area with specific focus on serving Spanish speakers and individuals with substance use disorders.

Email: **eroskey@wi.edu**

Lexie Thomas will be administering the diagnostic psychological assessments. She is third-year graduate student working towards her Doctorate in Clinical Psychology at the Wright Institute. She has clinical experience treating various populations that faced challenges related to complex trauma, the criminal justice system, severe mental illness/serious emotional disturbance, and chemical dependency. Currently she works in psychiatric emergency services where she performs assessments, brief therapy interventions, and individualize treatment planning to a diverse population of patients in acute crises.

Email: **lthomas@wi.edu**

Induni Wickramasinghe will be administering the diagnostic psychological assessments. She received her Master of Science in Psychology from Palo Alto University in 2021 and is currently working towards her doctorate. She has trained at the Palto Alto VA Telemental Health Clinic and with the San Francisco VA PTSD Clinical Team.

Email: **iwickramasinghe@paloaltou.edu**

19.0 End of Study Application

End of Study Application Form

To continue working on the Study Application:
Click on the section you need to edit in the left-hand menu. Remember to save through the entire Study Application after making changes.

If you are done working on the Study Application:
Important: Before proceeding, please go back to Section 4.0 Initial Screening Questions and **Save and Continue** through the form to make sure all the relevant sections and questions have been included. If you've changed any answers since you started, the branching may have changed. Your application will be incomplete and it will have to be returned for corrections.

Once you are sure the form is complete, click **Save and Continue**. If this is a new study, you will automatically enter the **Initial Review Submission Packet Form**, where you can attach **consent forms** or other **study documents**. Review the **Initial Review Submission Checklist** for a list of required attachments.

Answer all questions and attach all required documents to speed up your approval.

The UCSF IRB welcomes feedback about the IRB Study Application Form. Please click the link to answer a **survey** about the application form.

RESEARCH PLAN

Background and Significance:
Given that aerobic exercise has been found to improve brain health and neurogenesis[10], cognitive function[11], mood[12], sleep[13], and cardiovascular health[14], there is a strong rationale to determine if exercise may be an effective rehabilitative intervention for Veterans with combat related PTSD. Our group has also demonstrated that individuals with PTSD have lower rates of exercise compared to others without PTSD of the same age and sex[15], suggesting they may particularly benefit from a focus on exercise. Despite the high acceptance of exercise therapy for PTSD found in one study[16], and the considerable advantage of a treatment lacking stigma, to date there are no reported controlled trials for exercise in any population with PTSD.

Summary of Relevant Clinical Trials: At present, there is limited published data on the effects of exercise in Veterans with PTSD. There have been several pilot studies published suggesting that exercise reduces PTSD and associated mood and anxiety symptoms in children[17], adolescents[18], and civilian adults[19, 20]. These studies have been limited by lack of control conditions and small sample sizes. Nevertheless, the pilot work does indicate acceptability, feasibility, and promise of efficacy.

Exercise for Depression and Anxiety: Multiple controlled trials have demonstrated that exercise is effective for the treatment of depression both as an augmentation or stand-alone intervention[21]. The American Psychiatric Association has endorsed exercise as a primary treatment for major depression. A recent Cochrane review of exercise for depression showed that exercise had comparable efficacy to first line antidepressant treatment[22]. Similarly, meta-analyses of controlled trials of exercise for anxiety have shown that active treatment has potent effects in reducing symptoms in both clinical and non-clinical samples [23]. Although the mechanism underlying these results remains uncertain, it has been suggested that improvements in self-efficacy or mastery may be responsible for mood changes, or that long-term psychological benefits accrue from the blunting of psychological responses to stress following individual bouts of exercise, and that these effects accumulate over time independently of fitness changes[24]. A recent Cochrane review of exercise for anxiety and depression in children and young adults concluded that it makes little difference whether the exercises were of high or low intensity[25].

Exercise and Sleep: Exercise is endorsed by the American Sleep Disorders Association. Driver and Taylor published a narrative review of exercise and sleep studies and concluded from self-report studies that moderate and regular physical activity has therapeutic and sleep promoting benefits[26]. The most beneficial effect seems to come from improved fitness with aerobic endurance training and acute exercise that lasts for more than an hour, whereas exhaustive exercise of high-intensity and long duration is disruptive to sleep, decreasing REM sleep and increasing wakefulness. The authors reported that exercise increased slow-wave sleep, reduced REM sleep and delayed REM latency in already fit subjects. In clinical populations, self-report data indicates that regular exercise is a useful modality in treating disorders of initiating and maintaining sleep, as well as complaints of poor sleep quality[26].

Sleep: A Common Element in PTSD, psychomotor functioning, and brain health: Sleep disturbances are highly prevalent in PTSD: As many as 90% of people with PTSD report nightmares and insomnia, and even when nightmares are excluded, sleep disturbance is the most frequently reported symptom of PTSD[27]. The National Comorbidity Survey estimates that the lifetime prevalence of PTSD in the United States population is 7.8%[28]. Overall, insomnia is sufficiently frequent, and experienced by PTSD patients as sufficiently severe to warrant medical intervention- so much so that over 50% of PTSD patients on psychopharmacologic medications are prescribed trazodone or sedative hypnotics[29]. Further, residual sleep disturbance is highly prevalent in PTSD patients who have received PTSD specific treatment[30].

PTSD and Cardiovascular Health: Patients with PTSD are at increased risk of developing cardiovascular disease (CVD), leading to decreased quality of life, impaired function, and early mortality[31-36]. Traditional CVD risk factors, including smoking, hypertension, diabetes, dyslipidemia, and obesity do not explain the increased risk, suggesting treatment of these will not be sufficient to prevent PTSD-associated CVD[34, 37]. Several studies have now established the association between PTSD and CVD using objective cardiovascular testing. A study of Vietnam-era Veteran twin pairs found patients with PTSD had increased incidence of CVD events and decreased myocardial perfusion on cardiac Positron Emission Tomography scans[37]. Patients with PTSD also have higher levels of coronary artery calcium, a marker of atherosclerosis[38]. Dr. Beth Cohen, an internist and Co-Investigator on this proposal has demonstrated an association of PTSD with CVD using functional exercise

treadmill testing and stress echocardiography[39]. Given the convincing evidence that exercise has strong effects in protecting against the development of CVD (reviewed in[40]), the results from this trial have broad implications for the health and well-being of returning warfighters.

The Stress and Health Program is broadly focused on understanding the relationship between stress and physical health and developing and testing novel treatments to improve the health of Veterans impacted by combat trauma. Dr. Cohen leads a research program focused on the association of PTSD to Cardiovascular Disease (CVD). Dr. Christopher Owens, a vascular surgeon and Co-Investigator, is focused on mechanisms and treatment of peripheral vascular disease. We propose to measure both exercise capacity on treadmill testing and endothelial function in our subjects pre- and post-treatment to test: a) if improved exercise capacity predicts and is necessary for treatment response; and b) if endothelial function, a potent risk factor for cardiovascular disease and understudied in PTSD, is modifiable by exercise in a Veteran population with PTSD. Dysfunction of the endothelium and blood vessels cause increased thrombosis and impairments in blood flow that underlie atherosclerotic plaque formation and accelerated progression of CVD[41]. Several non-invasive physiologic measurements of vascular function are available, and these measures predict risk of future CVD events[42, 43]. Brachial artery flow mediated vasodilation (FMD) is the most widely accepted measure of endothelial function. Flow is occluded with a blood pressure cuff, and upon deflation of the cuff, in healthy endothelium, nitric oxide will be released in response to the increased blood flow and cause vasodilation. FMD is impaired early in the development of atherosclerosis, and lower FMD has predicted incident CVD events in several large cohort studies[42, 44].

Why Integrative Exercise? Aerobic exercises are a part of daily life of all military personnel during their service time. Exercise is familiar to Veterans and attractive to younger Veterans as a self-image boosting and mood stabilizing physical practice. In the past decade, while physical activity and aerobic exercise are still emphasized, mindfulness based practices have increasingly been adopted in military settings[45] and controlled trials of Mindfulness-Based Stress Reduction (MBSR) are currently underway with support from both DoD and VA. While a recently published controlled pilot study of MBSR in Veterans with PTSD found no reliable effects of MBSR on PTSD or depression on intention-to-treat analyses, completer analyses (>/= 4 classes attended) showed medium to large between-group effect sizes for the effect of MBSR on decreasing depression and increasing mental health-related quality of life[46]. Further, more Veterans randomized to MBSR had clinically meaningful reductions in PTSD symptoms at 4 month follow-up compared to treatment as usual[46]. The investigators in this important pilot study raised the question as to whether the mindfulness should be modified or tailored for Veterans, particularly with a focus on staying present with a non-judging attitude[46]. *Another recently published VA-study compared MBSR to present-centered group therapy (N = 116)[47]. At 2-month follow-up participants in the MBSR group had improved PTSD Checklist mean scores (range, 17-85; reduction of ≥10 considered minimal clinically important difference) from 63.6 to 54.4 vs 58.8 to 56.0 in the active control group, respectively (P < .001). Although participants in the mindfulness-based stress reduction group were more likely to show clinically significant improvement in self-reported PTSD symptom severity (48.9% vs 28.1% with control; (difference, 20.9%; 95% CI, 2.2%-39.5%; P = .03), they were no more likely to have loss of PTSD diagnosis (53.3% vs 47.3%, respectively. The authors concluded that MBSR, compared with present-centered group therapy, resulted in a greater decrease in PTSD symptom severity. However, the magnitude of the average improvement suggests a modest effect. A systematic review published at the same time examined the effectiveness of psychotherapies for PTSD in military and veteran populations and included 5 RCTs of Cognitive Processing Therapy (CPT) and 4 RCTs of prolonged exposure[48]. Forty-nine percent to 70% of participants receiving CPT and prolonged exposure attained clinically meaningful symptom improvement. However, approximately two-thirds of patients receiving CPT or prolonged exposure retained their PTSD diagnosis after treatment. The authors concluded that there is a need for improvement in existing PTSD treatments and for development and testing of novel treatments.* There is a large descriptive literature suggesting that breath training might have benefit for PTSD and stress-related medical conditions[49]. Might a program that integrated mindful breathing with other exercises, including both aerobics and strength training, be a good fit for Veterans? This is the approach taken in the Carl R. Darnall Army Medical Center's Warrior Combat Stress Reset Program in Fort Hood. This program includes movement exercises, self-regulation practices and yoga, with its emphasis on breathing. In the Warrior Combat Stress Reset Program, the emphasis was on helping to calm Veterans' mind, body, and spirit after returning from war.

In the current study, a program of exercise was created to take advantage of the large body of evidence demonstrating that aerobic exercise effectively improves many outcomes relevant to

Posttraumatic Stress (PTSD), the pilot studies indicating acceptability, feasibility and promise of efficacy for the effects of exercise on PTSD symptoms, while incorporating elements of mindfulness, including core tenets, such as non-judging attitude and mindful breathing.

Preliminary Studies

In our pilot study, we enrolled a total of 46 (9 women) veteran participants. Twenty-one subjects were assigned to exercise and 25 to the wait list. Note, randomization was conducted from block randomization lists from 4 strata defined by gender and age. We had a total of 9 dropouts: 5 from the Integrative Exercise (IE) group and 4 from the Wait List (WL) group. All 5 subjects from the IE group and 2 from the WL group dropped out in the first week after randomization and were lost to follow-up. One of the subjects who dropped from WL immediately following randomization indicated they elected to enroll in a fitness program outside of the research setting. We have 38 total completers (21 WL and 16 IE).

The clinical and demographic characteristics of our subjects in the pilot trial are presented in Table 1.

Table 1	*IE (n = 21)*	*WL (n =25)*	*Test statistic*	*All participants*
Age, mean (SD), years	*47.42 (15.94)*	*46.97 (14.25)*	*t(44) = .01*	*47.18 (14.88)*
Gender, no. (%)			$x^2(1) = .01$	
Male	*17 (80.95)*	*20 (80.00)*		*37 (80.43)*
Female	*4 (19.05)*	*5 (20.00)*		*9 (19.57)*
Race, no. (%)			$x^2(6) = 5.36$	
Caucasian	*12 (57.14)*	*12 (48.00)*		*24 (52.187)*
Black/African American	*4 (19.05)*	*8 (32.00)*		*12 (26.09)*
Asian	*1 (4.76)*	*2 (8.00)*		*3 (6.52)*
American Indian or Alaska Native	*0 (0.00)*	*1 (4.00)*		*1 (2.17)*
Native Hawaiian or Other Pacific Islander	*0 (0.00)*	*1 (4.00)*		*1 (2.17)*
More Than One Race	*3 (14.29)*	*1 (4.00)*		*4 (8.70)*
Other	*1 (4.76)*	*0 (0.00)*		*1 (2.17)*
Hispanic	*6 (28.57)*	*4 (16.67)*	$x^2(1) = .91$	*10 (22.22)*
Education duration, mean (SD), years	*16.10 (3.70)*	*15.40 (2.29)*	*t(44) = .78*	*15.72 (3.00)*
Baseline CAPS, mean (SD)	*64.25 (20.54)*	*59.12 (14.11)*	*t(43) = .99*	*61.40 (17.25)*

Effects of Integrative Exercise on Aerobic Capacity, PTSD symptoms, and WHOQOL Physical and Psychological Domains: *Figure 1 shows the change in World Health Organization Quality of Life (WHOQOL) Psychological and Physical Domains, mean metabolic equivalent (METS) on exercise treadmill testing, and scores on the Clinician Administered PTSD Scale (CAPS[50]) for Exercise vs. Wait List completers at baseline and end of study. Error bars represent standard errors of the means.*1) The WHOQOL-BREF[51, 52] instrument comprises 26 items, which measure the following broad domains: physical health, psychological health, social relationships, and environment. The WHOQOL-BREF is a shorter version of the original instrument and more convenient for use in large research studies or clinical trials. The Psychological Domain, our primary outcome, is derived from 6 items which index body image, negative & positive feelings, self-esteem, spirituality, and cognition. Each item has 5 response options with higher scores denoting higher psychological health. The mean score of items within each domain is used to calculate the domain score. Mean scores are then multiplied by 4 in order to make domain scores comparable with the scores used in the larger WHOQOL-100 scale[52]. *The group difference in change from baseline to 12 weeks, estimated from the mixed model fit to all time points, was 1.39 points greater improvement in Psychological Domain scores in the IE condition compared to Wait List, Cohen's d= 0.54, 95% C.I. = (.17, .91), p = .005.The relative improvement in the WHOQOL Physical Domain was 0.70 points, or d = .28, 95% C.I. = (-.21, .78), p = .25. Cohen's d is calculated*

here as the group difference in baseline-adjusted post-treatment scores divided by the standard deviation of baseline scores.

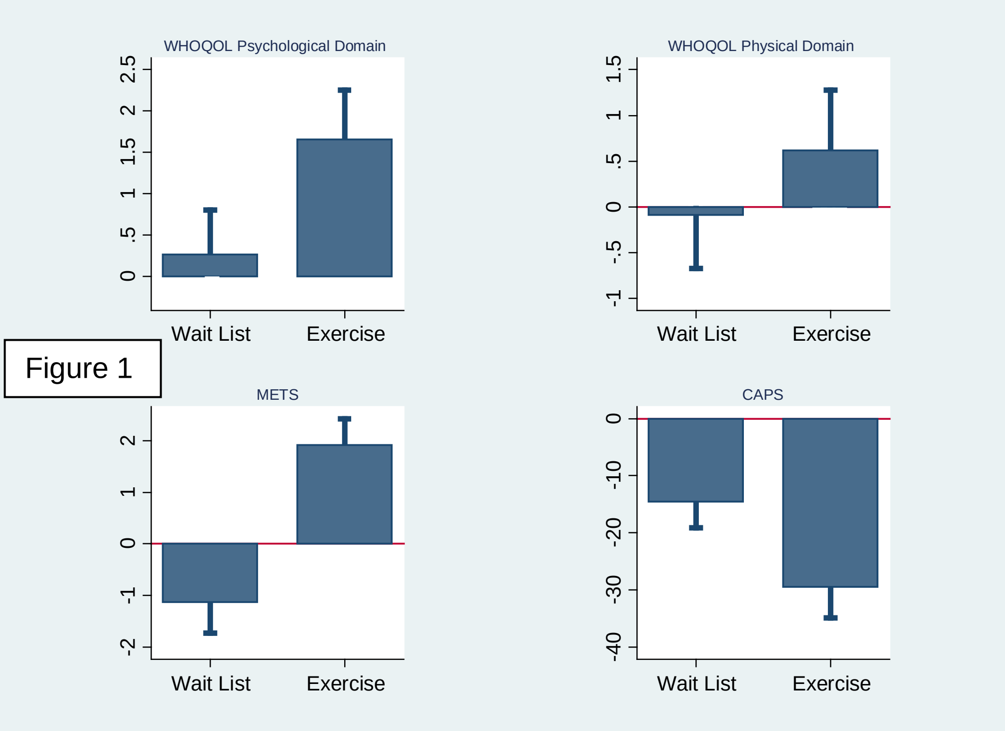

Figure 1

Aerobic fitness was assessed by measuring exercise capacity using a symptom-limited graded exercise treadmill test according to a standard Bruce protocol at baseline and end of treatment or wait list. (More details presented in Research Design and Methods.) Participants were asked to walk on a treadmill beginning at a workload of 20-30 watts and increasing by 20-30 watts every 3 minutes until reaching dyspnea, symptom-limited fatigue, chest discomfort, or electrocardiographic changes suggestive of ischemia[33, 53]. Metabolic equivalents (METS) were measured throughout and maximum exercise capacity was calculated as the total number of METS achieved. *IE subjects demonstrated that the exercise intervention produced a significant increase in exercise capacity. The WL subjects also showed a reduction in exercise capacity during the 12-week waiting period which demonstrates that subjects did not initiate an exercise program on their own. The group by time interaction from a mixed model analysis was highly significant (z= 5.2, p < 0.001), Cohen's d = 3.9.* This data also demonstrate objectively that our team was successful in engaging subjects in a program of fitness that produced a measurable impact on cardiovascular health.

PTSD symptom severity was measured with the CAPS which provides both a dimensional and categorical measure of PTSD. The CAPS measures frequency and intensity of PTSD-related symptoms. Possible scores range from 0 to 136. The CAPS interview was conducted in all subjects at baseline and after the 12-week treatment or monitor-only period. All of the instruments were conducted by an evaluator who was blind to treatment assignment. Subjects were instructed prior to each assessment to not disclose their treatment assignment to the evaluator. Figure 1 shows the mean change in CAPS total scores for Exercise vs. Wait List groups at baseline and end of study. *The differential improvement in CAPS score in the IE vs. the WL group was 15.9 points, Cohen's d = 1.13, z = 2.37, p = .018.* Overall our pilot data demonstrate favorable promise for the effects of Integrative Exercise on improving the total CAPS score. Figure 2 shows the correlation between change in mean Metabolic equivalent (METS) and CAPS score in the combined sample of IE vs WL completers. Note the predominantly negative values for METs in the wait list group.

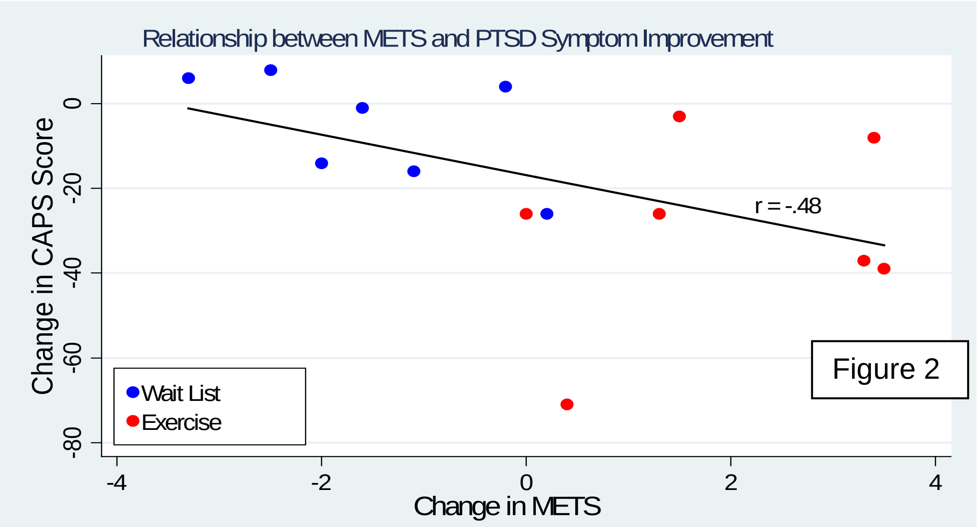

Figure 2

Preliminary data on measurement of endothelial function: Previous research has shown peripheral blood biomarkers of endothelial dysfunction increase and

flow mediated dilation (FMD) decreases after acute psychological stressors[54-56]. Studies also suggest *chronic* psychological stress from PTSD may impair vascular function, but they have been limited in their sample size and scope of vascular function measures. In a study of 28 medically healthy patients with and without PTSD, those with PTSD had significantly higher levels of peripheral biomarkers of endothelial dysfunction, and levels were correlated with severity of PTSD symptoms[57]. A separate study conducted in 100 police officers found higher levels of PTSD symptoms were associated with a nearly twofold decrease in FMD after adjustment for demographics and health behaviors.[58] *We studied FMD in 214 subjects at the San Francisco VAMC. Brachial artery FMD was evaluated after inflation of a blood pressure cuff to suprasystolic pressures for 5 minutes as described in the Measures Section. Veterans with PTSD (N= 67) relative to Veterans without PTSD (N= 147) had a significantly lower brachial artery FMD (5.8 ± 3.4% vs 7.5 ± 3.7%; p=0.003) adjusting for demographics, comorbidities, and treatment characteristics. This difference has significant predictive potential for the risk of CV events. In fact, in recent meta-analysis, it has been estimated that a 1% decrease in FMD is predictive of a 10% absolute increase in future CV events and mortality[59, 60].*

We obtained FMD data in a sub-sample of our pilot treatment study before and after IE (N= 6) or WL (N= 9). The results are presented in Table 2 below:

Group	*Baseline*	*End of Trial*	*Effect Size (d)*	*p*
Wait List (N=9)	*8.3% (s.e. = 1.1)*	*7.8 (s.e. = 1.1)*	*-.18*	*.73*
Integrative Exercise (N=6)	*9.6% (s.e. = 2.0)*	*13.7 (s.e. = 2.0)*	*.87*	*.09*

Although not statistically significant given the small sample size, these preliminary data provide additional support for the hypothesis that FMD, a potent risk factor for cardiovascular disease, is modifiable with integrative exercise in a population with PTSD.

Feasibility and Acceptability in the subjects who completed Integrative Exercise. *Note that all 5 of the dropouts assigned to IE left the study immediately after randomization. None of the subjects who initiated treatment dropped out. IE completers (N = 16) reported high satisfaction with the intervention. On the Feasibility and Acceptability Questionnaire (see Table 3 below), the most highly rated areas were perceiving benefit from this treatment, learning new skills and techniques, experiencing the treatment as adaptable to personal capabilities, and finding the instructors and intervention to be engaging. All completers said they would recommend this treatment to a friend. Though participants indicated slightly less satisfaction with the length of treatment, only 4 participants reported they thought the treatment was not "just the right length."*

Table 3. Feasibility and Acceptability Questionnaire - 16 IE completers

Item	***Mean (SD)***	***Median (Min-Max)***
Overall treatment impressions		
I feel like I benefitted from this treatment	*4.81 (0.40)*	*5 (4-5)*
This treatment taught me new skills and techniques	*4.75 (0.45)*	*5 (4-5)*
I would recommend this treatment to a friend who was dealing with similar issues	*4.69 (0.48)*	*5 (4-5)*
I was able to tolerate this treatment well	*4.44 (0.89)*	*5 (2-5)*
Content of intervention		
The instructors suggested modifications to match my fitness level/physical limitations to exercise	*4.75 (0.45)*	*5 (4-5)*
The instructors were engaging and made class fun	*4.75 (0.45)*	*5 (4-5)*
The breathing techniques covered in class were just right	*4.25 (0.58)*	*4 (3-5)*
We spent the right amount of time on each exercise	*4.31 (0.70)*	*4 (3-5)*
The exercises covered in class were just right	*4.19 (0.91)*	*4 (2-5)*
I wish other exercises had been included	*3.38 (1.15)*	*3 (1-5)*
Length of intervention		
*The 60 minute workouts were too long**	*4.13 (0.62)**	*4 (3-5)*
This treatment did not seem like too big of a burden	*4.00 (0.89)*	*4 (2-5)*
I wish the treatment had more sessions	*3.44 (1.41)*	*3.5 (1-5)*
The 36 session treatment (3 times per week for 12 weeks) was just the right length	*3.31 (1.35)*	*3 (1-5)*

Note: Response options ranged from 1 (strongly disagree) to 5 (strongly agree).
This item was reverse coded; the results indicate that most participants did **not think the 60 minute workouts were too long.*

Summary: There are strong converging lines of evidence that exercise can have positive effects on multiple functional domains relevant to PTSD: psychological and physical heath, sleep, and cardiovascular health. We have developed a novel protocol integrating the best elements of aerobics, strength training, and mindfulness-based breath training. We currently have a dedicated study team in place and with our success at recruitment and engagement, we are poised to complete a new definitive efficacy trial with an active control condition that will have sufficient power to properly evaluate whether an exercise intervention improves quality of life and broad measures of psychological and physical health in combat Veterans with PTSD. Because of the strengths of our team in PTSD-related comorbidities and clinical trials, we are in a unique position to test if exercise has positive effects on quality of life, psychological health, sleep, and cardiovascular fitness. It is our position that positive results in all of these domains would greatly enhance the attractiveness of this intervention to returning warfighters with PTSD and attract more Veterans into treatment. Further, the empiric data obtained from this project will help inform policy regarding therapeutics that serve the rehabilitation mission of RR&D.

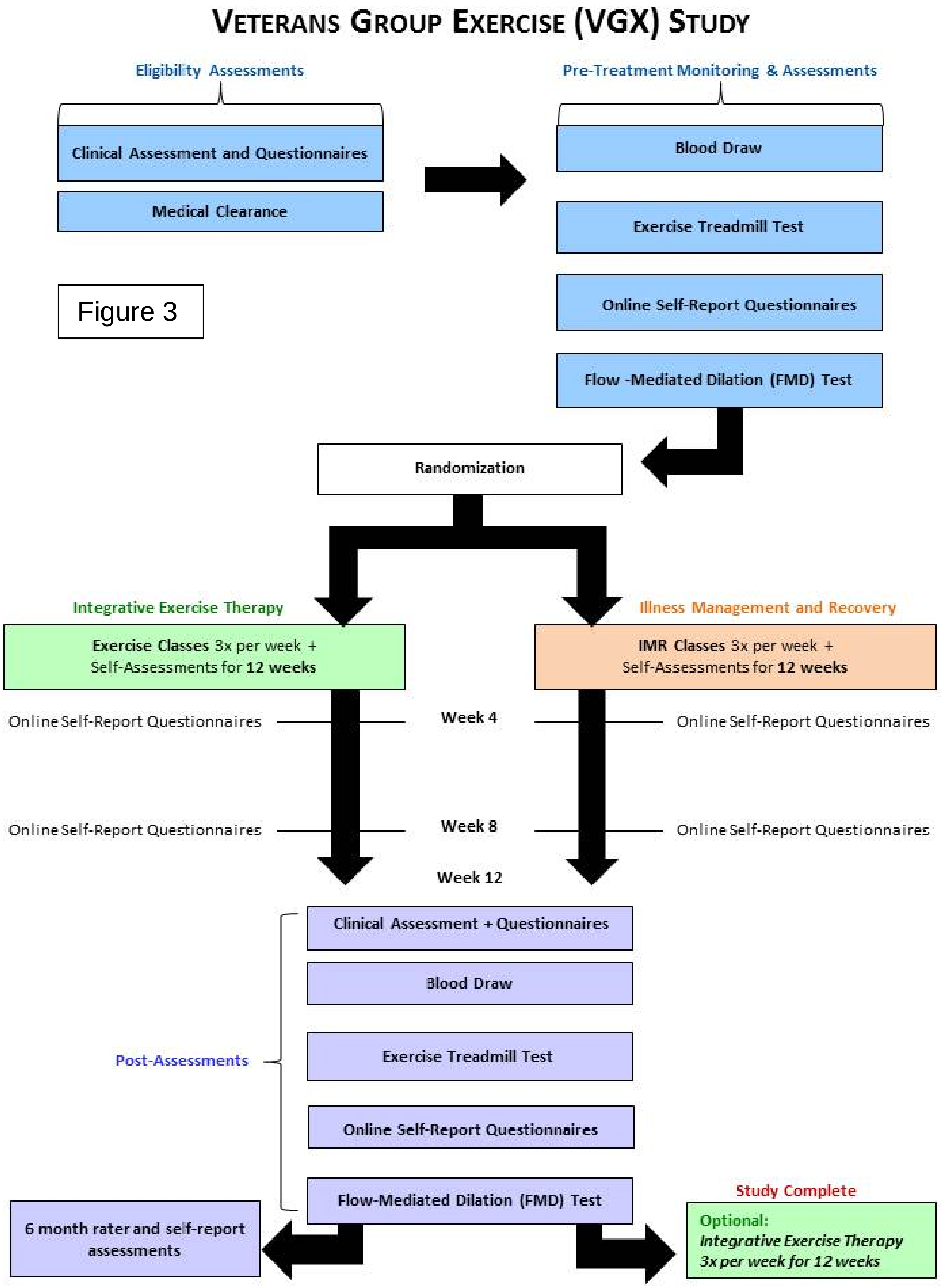

Figure 3

<u>Research Design and Methods:</u> (Figure 3)
Overview: Subjects with full PTSD will be recruited to participate in a parallel-groups trial. 104 subjects will be randomly assigned to one of two groups: one receiving treatment (12-week course of Integrative Exercise [IE]; Appendix 2), the other assigned to a 12-week Illness Management and Recovery (IMR) condition (Appendix 3). A stratified randomization strategy will be deployed to help ensure that the 2 conditions do not differ for subjects who are or are not engaged in current treatment for PTSD. Integrative Exercise will be delivered using manualized procedures described below. The therapists will review session-specific adherence checklists for

both conditions (see Appendices 2 & 3) at the conclusion of each session. Drs. Mehling and Boyd, who have extensive experience with delivering IE and IMR respectively, will rate recorded sessions and independently rate adherence. Participants will complete baseline assessments and an exercise treadmill and flow-mediated dilation (FMD) test prior to randomization. Integrative Exercise treatment involves a combination of aerobic and strength training exercises as well as concentration training based on mindful breathing techniques. Subjects will exercise 3 times weekly, with each total workout being approximately 60 minutes in length. After the 12-week intervention, participants will repeat the clinical assessment, exercise treadmill and FMD test. Measures of subjective sleep quality, PTSD symptoms, mood states – particularly depression, and quality of life (QOL), will be obtained in all subjects (Integrative Exercise and IMR) at baseline, weeks 4 & 8, and again following the end of 12 weeks. Subjects randomized to Integrative Exercise will be asked to rate their satisfaction with the intervention (i.e., acceptability and feasibility *and comparison with other PTSD treatments that participants may have experienced in the past*) at the end of 12 weeks and will have repeat clinical assessments at 6 months to examine durability of treatment gains.

Major Design Issue: The Control Condition
This remains our most challenging design decision given that our pilot study was conducted with a waitlist control condition. We have chosen IMR for several reasons. One, it is predominantly a health education oriented format and it allows us to match attention by study personnel across the two conditions. Further, it has been disseminated widely in the VA and is a mandated component of Psychosocial Rehabilitation and Recovery Center (PRRC) programs across the country. A recent meta-analysis[61] showed that dropout rates are around 20%, similar to our experience with IE. Finally, the curriculum is modular and is adaptable to different populations. (For more details see the Illness Management and Recovery Treatment section.)

Recruitment and Feasibility: Medically healthy men and women with chronic PTSD will be recruited from newspaper advertisements, web-based postings, flyers posted in San Francisco and regional community based outpatient clinics (CBOCs), and from the clinical PTSD Program at the SFVAMC which in the past 2 years treated 17,641 Veterans with PTSD. Further, we have acquired, with full IRB and SFVAMC R&D approval, a list of names and contact information of 5,200 Veterans in the San Francisco Bay Area from the Defense Manpower Data Center. Specifically, these are Veterans who are within a 50-mile radius from the SFVAMC and served in active duty between 2001 and March of 2014. This has substantially enabled our recruitment in the current pilot study and has allowed us to contact Veterans who are not currently enrolled in VA health care. The PTSD research program has successfully enrolled over 480 male and female PTSD and control subjects in the past 2 years. *Our goal will be to have at least 45% racial/ethnic minorities and at least 12% women in our sample (See Human Subjects).* Recruitment efforts will be enhanced by the Stress and Health Strategic Outreach and Recruitment/Retention (SOAR) program, which is supported in part by the Sierra Pacific MIRECC and will provide staff support and a host of essential functions and materials key to successful recruitment. Subjects who respond to recruitment solicitation will be initially screened by telephone and next invited for an assessment interview by study personnel. The screening clinician will obtain written informed consent at the time of the first diagnostic interview, prior to beginning the assessment.

Subjects: 104 subjects with PTSD will be randomized with an end goal of having 80 subjects with complete data for the entire trial. The 25% attrition rate is a conservative estimate based on our experience with this treatment and conducting other treatment studies in similar samples of PTSD subjects. There are at least 4 factors that account for this: 1) our staff and therapists are trained to be very "patient centered", 2) many patients with PTSD are pleased to have the opportunity to address the problem without relying on medications or trauma focused therapy, 3) all patients that participate in our RCTs and are randomized to attention control conditions are guaranteed access to the active treatment at study end at no cost, and 4) a bonus remuneration payment is provided to subjects that successfully complete the trial. Our current experience is that dropouts occur shortly after randomization. This is very similar to the experience of a large, controlled treatment trial for PTSD in an exclusively Veteran population led by Barbara Rothbaum at Emory University[3]. We have strong methods of retaining participants who drop out of treatment for follow-up assessments. Our research into retention in clinical trials [62] has identified several factors related to retention. Study teams that train their research associates and clinical evaluators to respond to participants with empathy, particularly in the face of participant complaints, have better retention. Monetary inducements offered at each assessment point have been shown to significantly reduce lost-to-follow-up (LTF) rates[63] and minimize the risk of differential LTF rates across treatments when there are differences in the desirability of treatments. These payments will increase compliance with assessments across the study[63].

Screening for Inclusion: Once subjects have initiated contact, they will be given more information about the study, either on the phone or through email or the Web. Prescreening interviews will be done by telephone after the subjects have given their verbal consent. They will be informed that some of the questions are about trauma and reminded that they can skip any question or stop the interview at any time. An internet-based survey will also be available if participants give electronic consent. The participants will have the option of calling the research team if they have questions or want further information. These subjects will be mailed or emailed a copy of the consent form which describes the next phase of the study. Procedures will not begin until written consent has been received.

Evaluation of PTSD Subjects:
Overview: All participants entering the study will participate in an audiotaped diagnostic interview conducted by Dr. Christiane Zenteno. Dr. Zenteno has conducted over 600 such interviews and is an expert in standardization and calibration of clinical evaluations. The Stress and Health Research Group conducts a weekly recruitment and consensus diagnostic conference, a weekly scientific administrative meeting to track the flow of subjects in the various protocols, and a weekly study design and data review conference. Discrepancies in the evaluation of subjects will be resolved by a consensus meeting.

Inclusion/Exclusion Criteria:
Inclusion Criteria: (1) Male and female Veterans between the ages of 18-69 who are physically able to participate in an exercise program; (2) Meet criteria for current full syndrome PTSD of at least 3 months duration, as indexed by the Clinician Administered PTSD Scale for DSM-5 (CAPS-5).
Exclusion Criteria: (1) Lifetime history of any psychiatric disorder with psychotic features, bipolar disorder, or mania, or meet criteria for moderate drug or alcohol use disorder within the past year as assessed by the Structured Clinical Interview for DSM-5; (2) Prominent suicidal or homicidal ideation; (3) Currently exposed to recurrent trauma or have been exposed to a traumatic event within the past 3 months; (4) Pregnant, have a clinically significant neurologic disorder, systemic illness affecting CNS function, history of seizure disorder, and/or physical disabilities making it impossible to use exercise equipment; (5) Contraindications for the exercise treadmill test which includes any acute coronary events (i.e., Myocardial Infarction) in the past 6 months; (6) Moderate to severe Traumatic Brain Injury (any history of head trauma associated with the onset of persistent cognitive complaints, neurological symptoms, or loss of consciousness > 30 minutes).
We will not exclude PTSD patients who are currently receiving individual or group therapy or patients who are currently taking antidepressant or anti-anxiety medication, but will apply the following criteria: patients must have been in treatment for at least 2 months, meet symptomatic criteria for inclusion, and not have plans to discontinue treatment during the course of the trial.

Measures:
Medical Screening: All subjects will meet with a study nurse practitioner at the CTSI Clinical Research Center at the San Francisco VA Medical Center. The nurse practitioner will review subjects' medical and surgical history as well as concurrent medications, and perform a physical exam. Vital signs, height, weight, and smoking status will be collected. The baseline lab tests for all participants will include a serum chemistry panel, liver function tests, thyroid functions, complete blood count, and differential, total cholesterol, HDL-cholesterol, LDL-cholesterol, triglycerides, glucose, hemoglobin A1c, and a urine toxicology screen. Depending on the subject's medical history, additional tests may be ordered to confirm that the subject is healthy and eligible to participate in the study. Female participants will be asked to provide a urine sample for pregnancy testing. Subjects who are pregnant or who have plans to become pregnant will be excluded from the study. The lab work results will be reviewed by Dr. Neylan. If significant abnormal findings are discovered, the subject will be referred to their primary care provider for treatment. Subjects will also be asked to complete the Physical Activity Readiness Questionnaire (American College of Sports Medicine [ACSM Guidelines]) to be cleared to exercise. If needed, each subject will perform a graded exercise test (ASCM Guidelines) to assess functional capacity. A portable screening device monitoring airflow, respiratory effort, pulse, and oximetry (ResMed ApneaLink Plus) will be used to prescreen for untreated obstructive sleep apnea (OSA) in all subjects. Cutoff criteria for apnea will be 10 apnea/hypopnea events per hour in bed.

Assessments:
The following assessments will be utilized in all subjects at baseline and after the 12-week treatment period. For subjects randomized to Integrated Exercise, a third set of assessments with the following measures will be conducted at the 6-month follow-up period. All of the instruments involving a clinical rater will be conducted by an evaluator who will not be conducting the treatment and will be kept blind to treatment assignment. Subjects

will be instructed prior to the assessment at 12 weeks not to disclose their treatment assignment to the interviewer*.*

1. The World Health Organization Quality of Life (WHOQOL-BREF)[51, 52]: The WHOQOL-BREF instrument comprises 26 items, which measure the following broad domains: physical health, psychological health, social relationships, and environment. The WHOQOL-BREF is a shorter version of the original WHOQOL-100 instrument and is more convenient for use in large research studies or clinical trials. The Psychological Domain, our primary outcome, is derived from 6 items which index body image, negative & positive feelings, self-esteem, spirituality, and cognition. Each item has 5 response options with higher scores denoting higher psychological health. The mean score of items within each domain is used to calculate the domain score. Mean scores are then multiplied by 4 in order to make transformed domain scores to a range of 4-20 comparable with the scores used in the WHOQOL-100.
2. Structured Clinical Interview for DSM-5-Research Version (SCID-5-RV,[64]). The SCID will be repeated after the 12-week treatment period.
3. Clinician Administered PTSD Scale for DSM-5 (CAPS-5,[65]). The CAPS-5 is a 30 item scale that provides both a dimensional and categorical measure of PTSD. The CAPS-5 will determine lifetime and current PTSD. The CAPS-5 items are rated with a single severity score that incorporates both frequency and intensity PTSD-related symptoms. In addition to assessing the 20 DSM-5 PTSD symptoms, questions target the onset and duration of symptoms, subjective distress, impact of symptoms on social and occupational functioning, improvement in symptoms since a previous CAPS administration, overall response validity, overall PTSD severity, and specifications for the dissociative subtype (depersonalization and derealization)[65].
4. Life Stressor Checklist-Revised (LSC-R): Structured clinical interview for lifetime exposure to stressful life events [66]. This structured clinical interview for lifetime exposure to stressful life events will be used to characterize the type of trauma exposure and age of occurrence(s) of different traumas in all subjects.
5. Addiction Severity Index Lite (ASI-Lite)[67]: The ASI-Lite Composite Score for Alcohol Use will provide a secondary measure of past month alcohol use severity, and the ASI-Lite number of years of alcohol use will provide a marker of lifetime alcohol use. The ASI-Lite Composite Score for Drug Use will provide a secondary measure of past month non-alcohol substance use severity, and the ASI-Lite number of years of drug use will provide a marker of lifetime non-alcohol substance use. The ASI-Lite is a valid and reliable standardized research interview to assess the occurrence and severity of alcohol and non-alcohol substance abuse. The ASI-Lite includes questions about the frequency, duration, and severity of problems over the subject's lifetime and in the past 30 days.
6. Smoking status[68] is a two-question categorical measure employed by the Centers for Disease Control and Prevention National Health Interview Survey, and categorizes individuals into one of three groups: (a) "Never smokers", adults 18 or over who have smoked fewer than 100 cigarettes in their lifetime; (b) "Former smokers", adults who have smoked at least 100 cigarettes in their lifetime but are not smoking at time of interview; and (c) "Current smokers", adults who have smoked at least 100 cigarettes over their lifetime and who are still smoking at time of interview.
7. Number of pack years[68] is a two-question continuous measure of smoking that utilizes the number of cigarettes per day multiplied by the number of years of smoking to calculate pack years of smoking.
8. Five Facet Mindfulness Questionnaire (FFMQ)[69, 70]: The FFMQ is a 39-item questionnaire derived from a factor analysis of other mindfulness questionnaires. It assesses five facets of mindfulness: observing, describing, acting with awareness, non-judging and non-reactivity to inner experience which represent elements of mindfulness as it is currently conceptualized. Items are rated on a Likert scale ranging from 1 (never or very rarely true) to 5 (very often or always true). The FFMQ has been shown to have good internal consistency (alpha coefficient range .72 to .92) in several samples and significant relationships in the predicted directions with domains related to mindfulness[69, 70]. The FFMQ allows a detailed assessment of changes as a function of mindfulness and therefore will be used as a secondary outcome.
9. Godin Leisure-Time Exercise Questionnaire[71, 72]: is a validated brief inventory assessing sedentary, work, recreational, and aerobic activity in a typical week. *This metric will be used to measure time spent in vigorous activity and will serve multiple purposes: a) It will be used to test if randomization effectively balances levels of baseline vigorous activity across the two groups; b) It will be used as a manipulation check to ensure that subjects randomized to IE engage in more vigorous activity after randomization than subjects randomized to IMR; c) It will be used as a secondary predictor of treatment response.*

10. The Physical Activity Self-Efficacy scale (PASE)[73]: The PASE will be used as an exploratory measure of perceived confidence to continue exercising in the face of competing day-to-day conditions.

Symptoms Scales:

The following measures will be obtained in all subjects (Integrative Exercise and IMR) at baseline, week 4, week 8, and after 12 weeks for the exploratory hypotheses in Aim 1. Subjects randomized to Integrated Exercise will also complete these measures at the 6 month follow-up period:

1. PTSD Checklist for DSM-5 (PCL-5)[74]. The PCL-5 is a validated self-report rating scale for assessing PTSD symptoms. It consists of 20 items that correspond to the DSM-5 symptoms of PTSD.
2. Symptom Check-List-90-Revised (SCL-90-R) [147]. The SCL-90-R is a standard self-report measure of general psychopathology. Scored for nine primary dimensions and three summary indices, the SCL-90-R manual reports extensive reliability and validity data.

Sleep and Alertness Assessment Methods:

1. Pittsburgh Sleep Quality Index (PSQI)[75]. This self-report measure provides a subjective assessment of sleep quality, sleep latency, sleep duration, sleep efficiency, sleep disturbances (including nightmares), use of sedative-hypnotics, and daytime energy. This index is now widely used and has been validated by polysomnography. *The PSQI will be our main measure of sleep quality.*
2. PSQI- PTSD Addendum (PSQI-A)[76]. The PSQI-A assesses disruptive nocturnal behaviors related to PTSD, such as hot flashes, nightmares, and episodes of terror during sleep. The total score ranges from 0 (normal) to 21 (severe). The PSQI-A has demonstrated good internal consistency and convergent validity. *The PSQI-A will be secondary measure of sleep quality that will be used in exploratory analyses.*
3. Insomnia Severity Index (ISI): The ISI is a valid and reliable self-report measure[77] that is a more specific index of perceived insomnia severity, as compared to the widely used PSQI measure which captures sleep disturbances of all types. The internal consistency of the ISI was found to be excellent (Cronbach's α= 0.74) and has been validated with both sleep diary and polysomnography[77]. *The ISI will be secondary measure of sleep quality that will be used in exploratory analyses.*

Exercise Treadmill Test: Aerobic fitness will be assessed by measuring exercise capacity using a symptom-limited graded exercise treadmill test according to a standard Bruce protocol. Participants will be asked to walk on a treadmill beginning at a workload of 20-30 watts and increasing by 20-30 watts every three minutes until reaching dyspnea, symptom-limited fatigue, chest discomfort, or electrocardiographic changes suggestive of ischemia[33, 53]. Heart rate, cuff blood pressure, minimum oxyhemoglobin saturation, and 12-lead electrocardiography will be recorded at rest, at each stage of exercise, and after four minutes of recovery. Continuous electrocardiographic monitoring of a 12-lead electrocardiogram will be performed throughout exercise. Metabolic equivalent (METS) will be measured throughout and maximum exercise capacity will be calculated as the total number of METS achieved. Maximum heart rate and time on the treadmill will also be measured. The main outcome measure for aerobic fitness will be exercise capacity (i.e., total number of METS achieved) during the treadmill test. This variable will be used to test if therapeutic gains associated with Integrative Exercise are associated with objective measures of exercise capacity.

Flow Mediated Dilation: Our main outcome will be flow mediated, brachial artery endothelium dependent vasodilation using a protocol endorsed by the International Brachial Artery Reactivity Task Force[78]. A trained vascular technician blinded to treatment status will perform all examinations. A blood pressure cuff will be inflated on the upper arm, blood pressure will be measured, and then the cuff will be inflated to suprasystolic pressures for 5 minutes. Following release of the cuff, there will be vasodilation and an increase in flow through the brachial artery (reactive hyperemia). FMD is calculated as the percentage of brachial artery dilation from baseline. Measures of baseline and reactive hyperemia blood velocity, blood flow, and shear stress will also be captured. Shear stress is recommended for normalization of FMD and blood flow is examined for quality control, with recommendations that blood flow increases at least 400% following release of cuff[79, 80]. These techniques have an interobserver variability of 0.05 ± 0.16% and intraobserver variability of <0.01 ± 0.15%.[78]

Randomization: After passing all screening procedures and found to be appropriate for the study, the study coordinator will randomize 104 subjects to either the 12-week treatment group (Integrative Exercise) or the IMR control group in a one-to-one ratio. A stratified randomization strategy will be deployed to help ensure that the two conditions do not differ for subjects enrolled in concurrent PTSD treatment. We will accomplish this by using two separate block randomization lists for those enrolled or not enrolled in concurrent treatment.

<u>NOTE:</u> There are a variety of factors that, when uncontrolled, may affect the study outcomes in such a way as to obscure or to create artifactual group differences. Accordingly, it is tempting to stratify on a whole host of

factors. Some examples would include age, gender, major depression, insomnia severity, or prior exposure to supervised exercise, etc. However, the attempt to control for all of these factors through randomization would be impractical and ineffective. *Moreover, post-hoc adjustments for unanticipated baseline differences risk capitalizing on chance differences and create multiple testing problems. Accordingly, the primary analyses will be the unadjusted analyses. Any baseline variables that show large group imbalances despite randomization will be examined as covariates in a secondary sensitivity analysis, but will not be included in the primary analyses. Substantial differences in inferences supported by the primary and secondary analyses would indicate a potential problem of randomization failure, and would warrant caution in interpreting results of the primary analyses , while consistency of inferences strengthen confidence in the primary analysis.*

Integrative Exercise Treatment (Overview): All participants will be medically screened before they can participate in the study. This will involve completing a physical activity readiness questionnaire (American College of Sports Medicine [ACSM] guidelines). Each subject, after being cleared to participate based on ACSM guidelines, will, if needed, perform a graded exercise test to assess functional capacity. The exercise program will be 12 weeks in duration, integrating a combination of aerobic and strength training with concentration training based on mindful breathing techniques (from yoga and mindfulness approaches recommended by Hoge[81]). Subjects will exercise 3 times weekly, with each total workout being approximately 60 minutes in length. These sessions will take place at the Embarcadero YMCA in San Francisco. All exercise will be supervised and documented by a trained professional in order to validate adherence and allow for program replications by others as part of an intervention program for PTSD. Exercises are adapted to individual participants' fitness levels and pre-existing health conditions or injuries.

The exercise program is designed with the following overall characteristics: It can be reproduced indoors as well as outdoors; it can be done in a group setting with participants at various fitness levels; it does not require a club membership or machines; it is accessible to combat Veterans of all socio-economic and educational levels; it is safe to Veterans with injuries; it is sufficiently different from usual fitness center programs to be attractive to 20-40 years old, younger Veterans; although it is not advertised as a form of psychotherapy, it will support the development of a "tool box" for coping with PTSD; the exercise instructions can be recorded on CD or MP3 and used in a private home. Overall, the goals of the exercises are twofold: 1) Increasing strength and cardio-vascular fitness, 2) Using the body that has physically returned to the homeland to bring the mind back home as well[81].

The sequence of exercises includes a variation of movements and loads for strength training and aerobic activities performed in a variety of ways, including continuous and interval training, as recommended by the American College of Sports Medicine (ACSM)[82-85] and using nonlinear periodization[84]. Props include dumbbells, stretch bands, and stepping platforms. (The full protocol is in Appendix 2.) Exercises are accompanied by appropriately paced, rhythmic and exciting, or calming music.

Following a moment of welcoming of participants with a brief review of the previous session, every session will start and end with 3-5 minutes of mindful breathing. Participants will be introduced to deep abdominal breathing, to pay attention to their breathing during the course of the session while working out and to dose the intensity of their exercising according to their ability to maintain breathing through his or her nose. Throughout, exercise movements will be closely coordinated with breathing phases, e.g. stretches with inhalation and weight pushes with exhalation. The mental focus on breathing during all exercises is a key ingredient of this integrative program to provide an embodied sense of mental centeredness and concentration. Mindful breathing is the cornerstone of mindfulness-based intervention for anxiety and depression[86-88].

The aerobic and strength program will begin with a warm-up and instructions for postural alignment supporting core engagement. Exercises will engage all major muscle groups in a systematic fashion that chains body parts together, but vary between sessions, starting with small weights and adding weights and aerobic intensity to pre-exhaust levels. Yoga moves and stretches, breathing and mindfulness instructions are woven into the program. Exercise sessions will end with a 5-minute cool-down period that returns to guided mindful breathing, fostering a physically experienced sense of body-based centeredness and relaxed concentration. For a detailed description of the intervention with its general principles, training components, class structure, mindful breathing teaching scripts, and physical exercises, we refer to the Manual in Appendix 2.

Illness Management and Recovery Treatment: The control condition will involve 36 hours of health education using the Illness and Management and Recovery (IMR) program[89] which is mandated by the

Uniform Mental Health Services directive to be a component of PRRCs at each VA medical center. The IMR control condition, sometimes referred to as Wellness Management and Recovery, is an educational curriculum focused on helping clients more effectively manage their illnesses to pursue their personal recovery goals. Originally developed for severe mental illness, it has been empirically tested in diverse patient groups including PTSD[5, 6]. IMR begins with the concept of recovery and education is predicated on a stress-vulnerability model of mental illness. The IMR workbook (See Appendix 3) has detailed adherence checklists which filled out by research staff at the end of each session. The 11 modules include the following topic areas which have been adapted for use in PTSD: recovery, practical facts about PTSD, stress-vulnerability, building social support, medications for PTSD, drug and alcohol use, reducing relapse, coping with stress, coping with persistent symptoms, getting needs met in the VA healthcare system, and living a healthy lifestyle[61, 89].

Data Quality Control

Data collection methods: The PI, Study Coordinator, data manager, and biostatistician will oversee data entry and management operations. Assessment test results will be entered into a password-protected database, which will be stored on a secure VA server. The data manager and the biostatistician will store "working" datasets as password-protected files on a SFVAMC secure server. Data will be entered into SQL Server tables using MS Access as a front-end or batch load from an external file. At a point of entry, form values are subjected to consistency edit checks (e.g. range and type verification, missing data). Scoring algorithms are applied where appropriate. Once data is entered into the database, edit checks are run for accuracy. On a regular basis, data management staff carries out quality edit checks. Data errors are corrected and previous / new values are automatically logged into a set of the database Audit tables. The reason for change, the date when the change has occurred and the person making the change are also captured by the audit trail mechanism and are stored in the database. A form tracking system is available to determine if all of the data forms have been received and entered. In addition to Windows authentication, the system also uses role-based model to access study data. It allows Data Management staff to grant users different level of access based on their active role in the study. Authorization is used to control access to browsing data as well as access to records modification and administrative functionality. Authorization rules are compliant with IRB regulations and the VA.

Data Analysis Plan

Study groups will be described in terms of baseline characteristics (including demographic and clinical measures, and baseline levels of the outcome variables) using appropriate summary statistics. Distributions of clinical measures and outcomes will be examined for presence of outliers and need for outcome transformations. The primary analyses will be *unadjusted* intent-to-treat analyses, with all subjects randomized included in the analyses. *Only the stratification factor, concurrent PTSD treatment status, will be included as a covariate in the primary analysis. Other baseline factors that remain unbalanced after randomization and are related to the outcomes will be analyzed in a separate sensitivity analysis to assess the robustness of the primary analysis*. All available time point data from any dropouts will be included in the analyses, using mixed models to accommodate the missing data. Residuals will be examined to ensure model assumptions are met. If we find violation of distributional assumptions, we will consider (1) transformations of or alternative distributions for the outcomes, and (2) bootstrapped 95% CI on the estimated intervention effect. If outliers are found, we will determine if data errors were overlooked during cleaning and check their influence via removal. The frequency and timing at which outcomes are obtained varies by measure. Some key measures, including the CAPS and the Exercise Treadmill Test, are measured at two time points, pre- and post-treatment, while self-report measures such as the WHOQOL, PCL and PSQI are measured at four time points. All of these outcomes will be analyzed with linear mixed models (LMM's). Measures with more than two measurement occasions make full use of the LMM strategy, whereas LMM's for pre-post measures reduce to ANCOVA as a special case, except for the added flexibility of modeling heterogeneous group variances. In analyses with intermediate time points, a number of modeling choices must be made, including whether to treat the time variable as continuous or categorical, the form of the within-subjects correlation matrix, and whether to allow for heterogeneity of variance across groups and/or time points. The best fitting model will be selected according to likelihood ratio tests (for nested models) or the Bayesian Information Criterion (BIC; for non-nested models) before examining any coefficients or test statistics. The LMM is an unbiased intent-to-treat analysis under assumptions of Missing At Random (MAR). *Any baseline clinical measures that correlate with dropout will be added to the models in a sensitivity analysis.* However, the possibility of informative missingness (i.e., missingness related to the missing outcome or other unmeasured variables) cannot be tested but also cannot be ruled out. Therefore, besides our primary LMM analytic strategy, we propose another sensitivity analysis for departure from MAR, based on copy reference (CR) imputation[90, 91]. In this

procedure, post-dropout data in the treatment group are multiply imputed from a model that assumes that treatment arm dropouts revert to the control group trajectory at a rate determined by the within-control group correlation structure. In the limiting case of participants who drop out of the treatment arm immediately after baseline, their imputed trajectory follows that of the control group. Dropouts from the control group are imputed from the control group only model based on baseline data and any observed time points before dropout. Multiply-imputed data sets are analyzed with the LMMs as proposed, with the estimates combined using Rubin's rules. The effect is to reduce the estimated difference between groups at end of trial, and yields a more conservative effect estimate than the LMM without imputation.

Primary Hypothesis Testing:

Hypothesis 1a: Subjects randomized to Integrative Exercise will have a greater improvement in quality of life as measured by the WHOQOL-BREF psychological domain compared to subjects randomized to IMR. For Hypothesis 1a, a linear mixed model will be estimated for the total score of the WHOQOL-BREF psychological domain using the Stata mixed procedure. Subjects will be entered as a random effect, while treatment group will be entered as a fixed effect. Treatment period (baseline, 4, 8, and 12 weeks) will be modeled both linearly and non-linearly (with a quadratic term for time), with the choice of model determined by a likelihood ratio test for the addition on the quadratic term. If the model with a linear function of time is retained, we will examine whether to include a random (subject-specific) effect for time, with the choice again made via a likelihood ratio test. (Four time points are insufficient to model both non-linear and random effects.) The hypothesis will be tested by the significance of the group (Integrative Exercise vs. IMR) by treatment period (pre- vs. post-treatment) interaction, with the baseline-adjusted group difference at Week 12 being the effect size.

Hypothesis 1b: Subjects randomized to Integrative exercise versus IMR will have greater improvements in cardiovascular health as measured by exercise capacity on the treadmill test. For Hypothesis 1b, a linear mixed model will be estimated for the total number of METS achieved on the treadmill test using the Stata mixed procedure. Subjects will be entered as a random effect, while treatment group and treatment period (randomization vs. end of trial Week 12) will be entered as fixed effects. The hypothesis will be tested by the significance of the group (Integrative Exercise vs. IMR) by treatment period (pre- vs. post-treatment) interaction, with the baseline-adjusted group difference at Week 12 being the effect size. Unlike Hypothesis 1a, the outcome is obtained at only two time points.

Hypothesis 1c: *Greater improvements in quality of life will be correlated with greater improvements in exercise capacity on treadmill, and changes in exercise capacity will partially mediate the effects of Integrative Exercise on quality of life. For hypothesis 1c, a mediation model will be specified as a linear path model using structural equation modeling software (Mplus 7.2). The model specifies a direct effect of treatment on both baseline-adjusted treadmill METS and baseline-adjusted WHOQOL Psychological Domain scores, with a path from METS to WHOQOL. The mediation path from treatment condition through change in METS to change in WHOQOL scores will be tested for significance using bootstrapped standard errors, and the mediation effect expressed as a percentage of the total effect that is mediated. We stress here that our design does not allow for an unambiguous causal interpretation of a mediation effect because the outcome and mediator are measured at the same time point. However, a finding of statistical mediation would be consistent with, and necessary for, a causal interpretation of the mediation effect.*

Exploratory Hypotheses: Subjects randomized to IE versus IMR will have greater improvements in additional health outcomes, including mood, subjective sleep quality, and brachial artery flow mediated vasodilation. These outcomes will be used to examine the internal consistency of the effects of treatment on the primary WHOQOL-BREF outcome. Scores will be computed for each of these outcome variables in the pre-randomization and post treatment (end of trial week 12) periods. Separate linear mixed models will be estimated for each outcome. Subjects will be entered as a random effect, while treatment group, time (pre- vs. post-treatment) will be entered as fixed effects. The hypothesis will be tested by the significance of the group (IE vs. IMR) by treatment period (pre- vs. post-treatment) interaction. We also will explore whether symptom improvement follows a dose-response relationship to amount of exercise in the IE group. Number of exercise sessions completed will be entered as a (possibly nonlinear) moderator variable in a mixed model prediction of symptom change over time within the IE group alone. The hypothesis will be tested by the significance of the interaction between time and number of completed sessions.

Hypothesis 2a: Participants randomized to Integrative Exercise will demonstrate greater improvements in PTSD symptoms as measured by the CAPS score compared to those in the IMR control group. For Hypothesis 2a, a linear mixed model will be estimated for the CAPS total score using the Stata mixed procedure. Subjects will be entered as a random effect, while treatment group and treatment period (randomization vs. end of trial

Week 12) will be entered as fixed effects. The hypothesis will be tested by the significance of the group (Integrative Exercise vs. IMR) by treatment period (pre- vs. post-treatment) interaction, with the adjusted group difference at Week 12 being the effect size.

Multiplicity Correction: *We plan to test primary hypotheses on primary outcomes at the nominal p < .05 significance level without adjustment. Significance tests for the secondary hypotheses will be adjusted for multiple outcomes using a resampling-based step-down procedure[92] to take advantage of the correlational structure among the secondary outcomes.*

Exploratory analysis of treatment moderators*: While we wish to avoid formal subgroup analyses because of lack of power and lack of pre-specified hypotheses, we nonetheless will explore potential treatment moderators (e.g., age, baseline level of vigorous activity) as a hypothesis-generating procedure to inform future studies. This will be achieved by examining interactions between subject characteristics and treatment effects. The emphasis here will be on estimated effect sizes rather than statistical significance (Kraemer et al. 2006[2]).*

Power Analysis: Our pilot data on WHOQOL-BREF, PTSD symptoms and METS units in response to exercise treatment are promising, but we are reluctant to base our power calculations entirely on effect sizes derived from pilot data, as these tend to be far too imprecise [93]. Rather we have powered the study to detect clinically meaningful effects on our primary variables. We conducted our power calculations for measures obtained at only pre- and post-treatment time points, because these are key outcomes and because the two time point case represents a lower bound for power when outcomes are measured more frequently. We used simulation (2000 replications each at multiple effect sizes) under the LMM to estimate power. Because our interest is in between-group differences in treatment outcomes, it is both appropriate and intuitive to express effect sizes in terms of standardized group differences at outcome. These are calculated from LMM-based marginal means and variances of post-treatment outcomes adjusted for baseline outcome measures. In the special cases where the LMM reduces to classical ANOVA-type models, these effect size estimates are identical to Cohen's d, and they have the same interpretation in more complex models. Our proposed total sample size of 80 completers yields power of 80% at alpha = .05 to detect standardized effects (model-estimated differences between the two groups at Week 12) of d = .5, assuming within-participant correlations of .7, which is a somewhat conservative correlation compared to our pilot data (r = .8) and previous experience in our lab. *Using standard deviation estimates from similar patient populations studied in our lab, we estimate that an effect size of d = .5 translates to a difference in CAPS scores of approximately 8 points, WHOQOL scores of 1.3 points, and METS scores of 2.7 units, which we believe are clinically meaningful effects and which are somewhat smaller than observed in our pilot data. There are no existing effect size estimates for calculating power for the mediation hypothesis, Hyp. 1c, and our pilot data sample is too small to provide reasonable estimates of mediation effects, as these depend on the relationship among several coefficients each of which is estimated with considerable error in a small sample. However, taking the point estimates of our pilot treatment effects on WHOQOL and CAPS at face value, we will have 80% power to detect a mediation effect of approximately 40% of the total effect of treatment on WHOQOL and 22% of the total effect on CAPS scores, using the method of Vittinghoff, Senn, and McCullough[94]. The mediation effects estimated from our pilot data are approximately 18% for both WHOQOL and CAPS, but with 95% C.I.'s ranging from -25% (i.e., an effect in the wrong direction) to more than 60%, so these estimates provide little information. Still, our proposed sample size will be able to detect a fairly substantial mediation effect if it exists, and a smaller effect is not likely to be clinically important.*

Intent-to-treat (ITT) analysis: Under assumptions of missing at random (MAR), the mixed model yields valid ITT estimates by including dropouts as missing data points. In the two-time point case, in which dropouts have baseline measures but no post-treatment measures, these observations contribute little to the analysis, and including dropouts in addition to the 80 completers has no appreciable effect on either effect estimates or power. In analyses with intermediate time point measures, power should be improved slightly by including mid-point dropouts in addition to the 80 completers (i.e., additional data, even if incomplete, can only improve power under the assumption of MAR). However, as discussed the Data Analysis Plan, even though the MAR assumption may be reasonable, it is not testable, and it is prudent to conduct a sensitivity analysis to the MAR assumption using other ITT methods. The proposed copy reference (CR) imputation method essentially assigns the treatment dropouts to a post-dropout trajectory similar to the control group, while still assigning them to the treatment group for analysis. It thus reduces estimated effect size in a principled way compared to the MAR analysis. This does not affect power directly, but it does so indirectly by reducing the effect size estimate. In our simulations, a worst case scenario has all of the dropouts occurring immediately after randomization, with no data beyond baseline. This would reduce an effect size of .5 to approximately .4, for which we would have power of approximately .64 rather than .80.
