# Supplementary material for: Group integrated exercise versus recovery class for veterans with posttraumatic stress disorder: a randomized clinical trial
Source: BMC Psychiatry. 2025 Feb 28;25:185. doi: 10.1186/s12888-025-06638-1 (PMC11871828; doi:10.1186/s12888-025-06638-1)
Supplement: Supplementary file 3 — Supplementary Material 3: Supplemental Figure 1S. Individual Responses to Feasibility and Acceptability Questionnaire- Integrated Exercise Group. Participants responded to each statement on a 6-point Likert type scale, with 0-2 scores indicating disagreement (tan colors) and 3-5 (green colors) indicating agreement. Supplemental Figure 2S. Individual Responses to Feasibility and Acceptability Questionnaire- Recovery Group. Participants responded to each statement on a 6-point Likert type scale, with 0-2 scores indicating disagreement (tan colors) and 3-5 (green colors) indicating agreement. Supplemental Figure 3S: WHOQOL Psychological Domain. Supplemental Figure 4S. Change in WHOQOL Psych in IE and REC conditions by setting and number of sessions attended. Supplemental Table 1S Demographic and Clinical Characteristics of In-Person and Remote Samples. [file 12888_2025_6638_MOESM3_ESM.docx]

Supplementary Materials

Supplemental Figure 1S. Individual Responses to Feasibility and Acceptability Questionnaire- Integrated Exercise Group


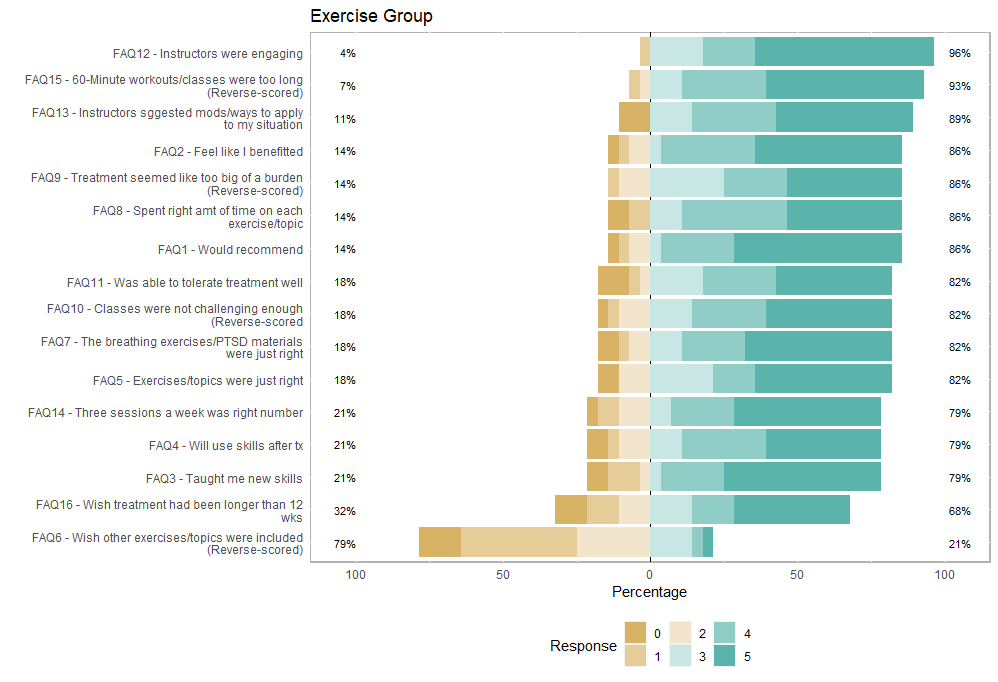


Participants responded to each statement on a 6-point Likert type scale, with 0-2 scores indicating disagreement (tan colors) and 3-5 (green colors) indicating agreement.

Supplemental Figure 2S. Individual Responses to Feasibility and Acceptability Questionnaire- Recovery Group


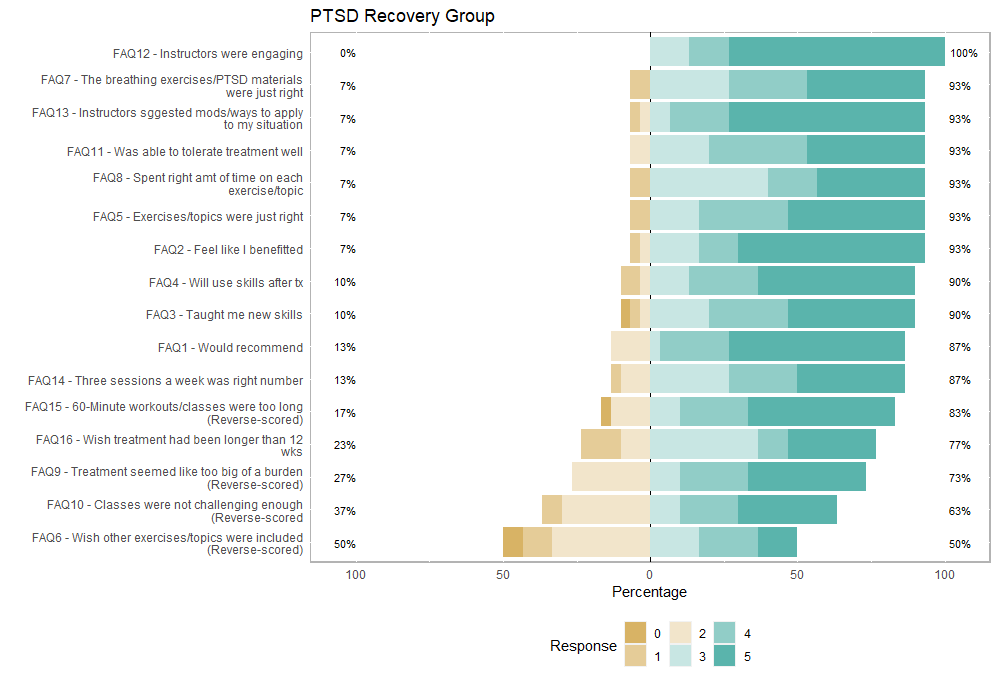


Participants responded to each statement on a 6-point Likert type scale, with 0-2 scores indicating disagreement (tan colors) and 3-5 (green colors) indicating agreement.

Supplemental Figure 3S: WHOQOL Psychological Domain


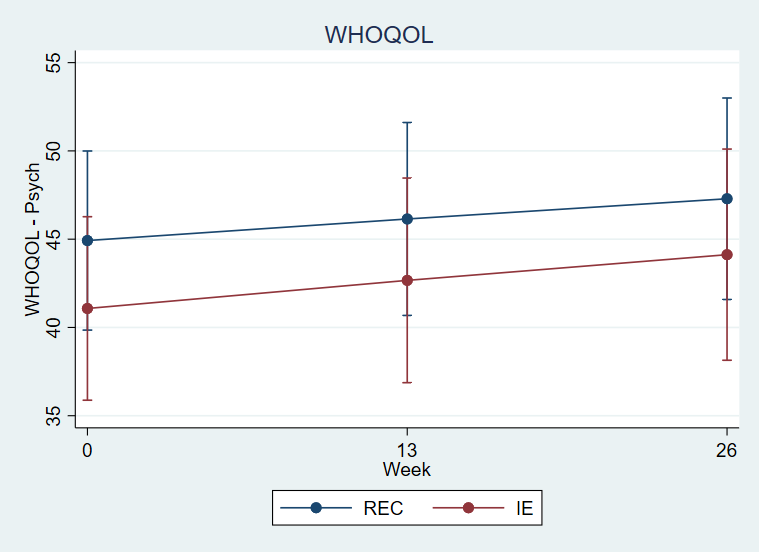


Supplemental Figure 4S. Change in WHOQOL Psych in IE and REC conditions by setting and number of sessions attended


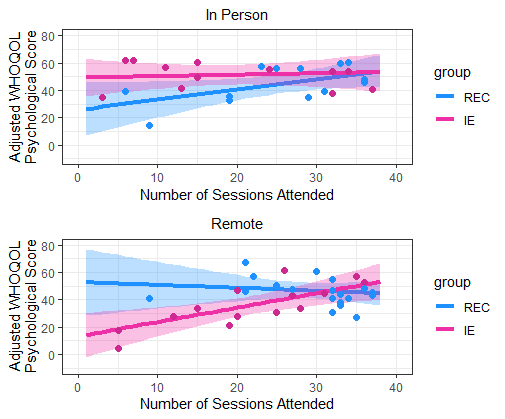


Supplemental Table 1S Demographic and Clinical Characteristics of In-Person and Remote Samples

|  | **In-Person** | **Remote** | **p-value** |
| --- | --- | --- | --- |
|  | **N=37** | **N=47** |  |
| Age | 56.3 (11.1) | 48.1 (11.1) | <0.01 |
| Sex |  |  | 0.03 |
| Male | 30 (81%) | 28 (60%) |  |
| Female | 7 (19%) | 19 (40%) |  |
| Race |  |  | 0.04 |
| American Indian/Alaska Native | 1 (3%) | 3 (6%) |  |
| Asian | 6 (16%) | 1 (2%) |  |
| Black or African American | 8 (22%) | 13 (28%) |  |
| White | 16 (43%) | 28 (60%) |  |
| Other | 6 (16%) | 2 (4%) |  |
| Ethnicity |  |  | 0.64 |
| Hispanic or Latino | 5 (14%) | 9 (19%) |  |
| Non-Hispanic or Latino | 26 (70%) | 33 (70%) |  |
| Unknown | 6 (16%) | 5 (11%) |  |
| Education |  |  | 0.11 |
| High School Graduate / GED | 0 (0%) | 4 (9%) |  |
| Some College | 14 (39%) | 15 (33%) |  |
| Associate Degree | 7 (19%) | 2 (4%) |  |
| Bachelors Degree | 6 (17%) | 8 (17%) |  |
| Some Graduate School | 3 (8%) | 2 (4%) |  |
| Masters Degree | 5 (14%) | 14 (30%) |  |
| Doctoral Degree | 1 (3%) | 1 (2%) |  |
| VA Service-Connected Disability |  |  | 0.13 |
| No | 6 (16%) | 14 (30%) |  |
| Yes | 31 (84%) | 32 (70%) |  |
| ASI Alcohol |  |  |  |
| Smoking history |  |  | 0.97 |
| Current Smoker | 6 (16%) | 8 (18%) |  |
| Former Smoker | 10 (27%) | 12 (27%) |  |
| Never Smoked | 21 (57%) | 24 (55%) |  |
| CAPS_Total_baseline | 31.2 (8.1) | 31.6 (8.4) | 0.81 |
| PCL5 Total | 42.4 (16.1) | 41.4 (12.5) | 0.75 |
| WHOQOL Physical Health | 53.2 (16.1) | 50.2 (14.0) | 0.37 |
| WHOQOL Psychological | 44.8 (17.6) | 41.2 (15.9) | 0.33 |
| WHOQOL Social Relationships | 45.0 (25.0) | 44.2 (23.1) | 0.87 |
| WHOQOL Environment | 57.4 (18.3) | 57.8 (14.2) | 0.92 |
| WHOQOL Overall Heatlth (Q. 2) | 41.9 (27.0) | 41.3 (24.8) | 0.92 |
| FFMQ Observing | 27.4 (5.9) | 22.7 (9.8) | 0.01 |
| FFMQ Describing | 25.9 (6.8) | 20.8 (8.9) | <0.01 |
| FFMQ Acting with Awareness | 25.7 (5.9) | 21.5 (9.2) | 0.02 |
| FFMQ Nonjudging of Inner Experience | 25.3 (7.0) | 22.4 (10.7) | 0.15 |
| FFMQ Nonreactivity to Inner Experience | 20.9 (4.1) | 17.6 (7.2) | 0.02 |
| GLTEQ Activity Score | 38.7 (37.5) | 25.2 (21.2) | 0.04 |
| Physical Activity Self-Efficacy | 2.8 (1.0) | 2.7 (0.8) | 0.70 |
| DERS Total Score | 87.9 (19.7) | 87.7 (22.6) | 0.96 |
| Emotion Regulation - Cognitive Reappraisal | 4.8 (1.2) | 4.7 (1.1) | 0.70 |
| Emotion Regulation - Expressive Suppression | 4.6 (1.3) | 3.9 (1.3) | 0.02 |
| MAIA Noticing | 3.2 (1.2) | 3.2 (0.9) | 0.99 |
| MAIA Not-Distracting | 1.8 (0.8) | 1.9 (0.8) | 0.61 |
| MAIA Not-Worrying | 2.8 (0.9) | 2.7 (0.7) | 0.35 |
| MAIA Attention Regulation | 2.9 (1.1) | 2.8 (0.8) | 0.64 |
| MAIA Emotional Awareness | 3.3 (1.2) | 3.3 (0.8) | 0.94 |
| MAIA Self-Regulation | 2.9 (1.2) | 2.8 (0.9) | 0.53 |
| MAIA Body Listening | 2.5 (1.3) | 2.3 (1.0) | 0.54 |
| MAIA Trusting | 3.1 (1.1) | 2.9 (1.1) | 0.40 |
| Positive States of Mind | 10.6 (3.5) | 9.5 (4.5) | 0.24 |
| SCL90 Global Severity Index | 1.2 (0.7) | 1.2 (0.5) | 0.77 |
| PSQI | 10.8 (3.9) | 12.3 (3.6) | 0.08 |
| PSQI PTSD Addendum Global Score | 7.8 (4.5) | 8.3 (3.7) | 0.56 |
| Insomnia Severity Index | 15.2 (6.8) | 16.1 (5.4) | 0.48 |
| Physical Activity Self-Efficacy | 2.8 (1.0) | 2.7 (0.8) | 0.70 |

Data are presented as mean (SD) for continuous measures, and n (%) for categorical measures. Acronyms: REC- Recovery Class, IE- Integrated Exercise, ASI- Addiction Severity Index, WHOQOL- World Health Organization Quality of Life, CAPS- Clinician Administered PTSD Scale, PCL-5- PTSD Checklist for DSM5, SCL-90- Symptom Check-List-90-Revised, FFMQ- Five Facet Mindfulness Questionnaire, GLTEQ- Godin Leisure-Time Exercise Questionnaire, PASE - Physical Activity Self-Efficacy scale, DERS- Difficulties in Emotion Regulation Scale, ERQ - Emotion Regulation Questionnaire, MAIA- Multidimensional Assessment of Interoceptive Awareness, PSOM - Positive States of Mind, PSQI- Pittsburgh Sleep Quality
